# Selenium supplementation for preventing cancer therapy-related toxicities: a systematic review and meta-analysis of clinical trials

**DOI:** 10.3389/fnut.2026.1940806

**Published:** 2026-10-07

**Authors:** Chuyue Chang, Juncheng Wei, Mengfei Li, Tingyi Chang, Yakun Guo, Miao Jiang

**Affiliations:** 1 Dongzhimen Hospital, Beijing University of Chinese Medicine, Beijing, China; 2 College of Life Sciences, Beijing University of Chinese Medicine, Beijing, China

**Keywords:** cancer therapy, chemotherapy, meta-analysis, oral mucositis, radiotherapy, selenium, systematic review, treatment-related toxicity

## Abstract

**Background:**

Trials of selenium for cancer supportive care vary in treatment context, formulation, dose, toxicity domain, and outcome definition. We evaluated the efficacy and safety of selenium supplementation for preventing or reducing cancer therapy-related toxicities.

**Methods:**

PubMed, Web of Science Core Collection, and the Cochrane Library were searched through June 30, 2026. Comparative clinical trials conducted during cancer therapy compared selenium with placebo, no selenium, observation, or selenium-free standard care. Two reviewers independently completed study selection, data extraction, outcome evaluation, and risk-of-bias assessment, with third-reviewer adjudication when required. Severe oral mucositis during head-and-neck radiotherapy or chemoradiotherapy was the primary outcome; broader pools were exploratory. Fixed- or random-effects analyses were supplemented by restricted maximum-likelihood models with Hartung-Knapp confidence intervals. Conference-abstract and derived-odds-ratio analyses were restricted to supplementary sensitivity analyses. Certainty was assessed using the Grading of Recommendations Assessment, Development and Evaluation (GRADE) framework. The protocol was registered in the International Prospective Register of Systematic Reviews (PROSPERO; CRD420261448497).

**Results:**

Of 444 records, 333 unique records were screened. Forty-one of 49 sought reports were assessed in full text, and 31 reports representing 30 study groups were included; 17 groups contributed to the main analysis and 13 to supplementary evidence. Overall risk of bias was judged to be Some concerns for nine studies and High risk for eight. The four-study primary analysis (188 participants) was inconclusive (risk ratio [RR] 0.89, 95% confidence interval [CI] 0.50 to 1.56; *I*^2^ 44.9%; very low certainty), as was the Hartung-Knapp analysis (RR 0.88, 95% CI 0.35 to 2.23). Five conventional exploratory confidence intervals crossed the null under Hartung-Knapp analyses. Across domains, the pooled estimate remained below the null (baseline RR 0.60, 95% CI 0.42 to 0.83; Hartung–Knapp RR 0.60, 95% CI 0.40 to 0.89; 11 studies), but heterogeneity was substantial and the certainty of evidence was very low.

**Conclusion:**

Current evidence does not establish prevention of severe oral mucositis, but the consistent cross-domain direction and several domain-specific exploratory estimates suggest that selenium may attenuate selected treatment-related toxicities, particularly gastrointestinal and hematologic events, in appropriately selected settings. Because the majority of estimates are based on few, heterogeneous, and high-risk studies, these signals should inform targeted trials rather than routine universal supplementation.

**Systematic review registration:**

This systematic review was registered with PROSPERO. Unique Identifier: CRD420261448497.

## 1 Introduction

Cancer therapy-related toxicities can interrupt treatment, impair quality of life, and increase supportive-care needs. These complications can reduce oral intake, worsen nutritional status, prompt treatment delay or dose reduction, and compromise treatment continuity. Their prevention is particularly important during intensive systemic therapy and when radiotherapy exposes anatomically sensitive structures such as the oral cavity, salivary glands, pelvis, kidneys, or peripheral nerves (1–3).

Clinical nutrition in oncology extends beyond energy and protein replacement to early identification and individualized management of nutrition-impacting symptoms during systemic therapy and radiotherapy (3). Within this framework, a micronutrient strategy is clinically relevant only when it has a plausible biological target, adequately characterized exposure and baseline status, and evidence that a potential supportive benefit does not compromise cancer treatment (4, 5).

Selenium is an essential trace element incorporated into selenoproteins, including glutathione peroxidases and thioredoxin reductases, that participate in redox homeostasis, antioxidant defense, and immune regulation (6–9).

Because radiation and cytotoxic therapy can injure normal tissues through oxidative and inflammatory pathways, sufficient selenium availability could help protect non-malignant tissues and attenuate treatment-associated injury. This rationale remains context-dependent because selenium compounds can exert antioxidant or pro-oxidant effects according to chemical form, dose, tissue, and baseline selenium status (4, 6).

Recent systematic reviews describe heterogeneous effects across toxicity outcomes and no consistent survival or quality-of-life benefit in unselected patients, while suggesting that selenium-deficient patients may be a more promising subgroup (4, 5). The resulting evidence is nevertheless difficult to interpret as a single body of efficacy data. Trials differ in selenium compound, route, dose, timing, baseline selenium status, comparator design, cancer treatment, and toxicity definition. Outcomes range from oral mucositis to gastrointestinal, hematologic, renal, neurologic, salivary, pain, quality-of-life, and safety measures.

Pooling all available outcomes would sacrifice clinical meaning, whereas restricting synthesis to one narrow outcome would omit much of the trial evidence. We therefore organized the review from narrow to broad: a clinically coherent primary analysis, domain-specific exploratory analyses, deliberately broad cross-domain consistency checks, and separate displays for incompatible effect measures or single-study evidence.

The objective was to evaluate the efficacy and safety of selenium supplementation for preventing or reducing cancer therapy-related toxicities. Severe oral mucositis during head-and-neck radiotherapy or chemoradiotherapy was selected as the primary quantitative outcome because it provided the most coherent set of compatible comparative event data. All wider syntheses were interpreted as exploratory.

## 2 Methods

### 2.1 Protocol and registration

The review protocol was registered in the International Prospective Register of Systematic Reviews (PROSPERO; CRD420261448497).

### 2.2 Design and eligibility

This systematic review and meta-analysis was reported in accordance with the Preferred Reporting Items for Systematic Reviews and Meta-Analyses (PRISMA) 2020 statement (10, 11). The protocol specified a clinical-trials synthesis rather than a randomized controlled trial (RCT)-only synthesis because the evidence base included randomized trials, older comparative clinical studies, and supplementary safety or contextual reports. Protocol amendments clarified full-text accounting, metadata, model-robustness analyses, and the handling of supplemental data verified against the source reports. These amendments did not change the review question, eligibility criteria, primary outcome, effect direction, or synthesis hierarchy.

Main-analysis studies enrolled patients with cancer receiving cancer-directed treatment and compared selenium supplementation with placebo, no selenium, observation, or standard care without selenium. Eligible treatments included chemotherapy, radiotherapy, concurrent chemoradiotherapy, high-dose chemotherapy with hematopoietic stem-cell transplantation, and radioiodine therapy. Selenium-specific attribution had to be possible. Duplicate or companion reports were linked and were not counted as independent studies.

Eligible efficacy outcomes were treatment-related toxicities, their severity or duration, and validated toxicity scores. Eligible safety outcomes included selenium-attributed adverse events, serious adverse events, dose-limiting toxicity, and treatment interruption or completion. Quality-of-life, nutritional, performance, selenium-status, tumor-response, and survival measures were retained as contextual outcomes when reported but were not combined with toxicity outcomes. Phase I or dose-escalation reports, single-arm safety studies, conference reports, established lymphedema-treatment studies, multicomponent interventions without selenium-specific attribution, and reports without retrievable full text were mapped as supplementary, background, excluded, or unavailable evidence as appropriate rather than being automatically entered into comparative effect pools.

### 2.3 Information sources and search strategy

PubMed, Web of Science Core Collection, and the Cochrane Library, including the Cochrane Central Register of Controlled Trials (CENTRAL) and Cochrane Reviews, were searched through 30 June 2026. The search combined selenium terms with cancer and cancer-treatment toxicity concepts. Full database-specific strategies, execution dates, and export counts are reported in Supplementary Table S1—supplemental searching identified three additional reports. Raw database exports were preserved and deduplicated before screening.

### 2.4 Study selection

CC and JW independently screened all titles and abstracts and assessed full-text reports against predefined eligibility criteria. Uncertain title or abstract records were retained for full-text assessment. Full-text reports were assigned a scientific decision and an evidence role. Disagreements were resolved through discussion; if consensus could not be reached, ML served as the third reviewer. Unretrieved reports were recorded as access limitations and were not treated as scientific full-text exclusions. The full-text exclusion list is provided in Supplementary Table S2.

### 2.5 Data collection and outcome preparation

CC and JW independently extracted all study data and evaluated outcome eligibility using standardized forms. The forms captured study design, country, setting, cancer type, therapy context, selenium compound, route, dose, timing, duration, comparator, outcome definition, toxicity grade or threshold, time point, event counts, denominators, means, standard deviations, reported effect measures, original comparison direction, and source location. Extraction or evaluation discrepancies were resolved through discussion and, when necessary, by ML as the third reviewer. Analysis-specific denominators were retained because some studies used per-protocol, intention-to-treat, crossover, cycle-level, or outcome-specific populations. Derived effect sizes were used only when the calculation method and source data were recorded. Outcome definitions and poolability decisions are reported in Supplementary Table S4, and effect extraction, processing, and direction harmonization are reported in Supplementary Table S6.

For harmful dichotomous outcomes, risk ratios (RRs) were expressed as selenium vs. control so that RR values below 1 favored selenium. Reported odds ratios (ORs) were not converted to or combined with event-count RRs when arm-level data were unavailable. Continuous outcomes were synthesized as mean differences (MDs) only when scales and time units were compatible. Laali 2020 reported oral mucositis onset in weeks; the means and standard deviations for both arms were multiplied by seven before combination with Wang 2020, which reported onset in days.

### 2.6 Risk of bias

CC and JW independently assessed risk of bias for the 17 main-analysis studies using Risk of Bias 2 (RoB 2)-style domains covering the randomization process, deviations from intended interventions, missing outcome data, outcome measurement, selection of reported results, and overall risk (12). Disagreements were resolved by discussion and, when required, adjudication by ML. Domain-level support and final judgments are presented in Supplementary Table S5 and Supplementary Figures S12 and S13.

### 2.7 Synthesis and statistical analysis

The primary meta-analysis assessed severe oral mucositis, defined as grade 3 or higher or grade 3–4 oral mucositis/stomatitis during head-and-neck radiotherapy or chemoradiotherapy. Exploratory analyses examined expanded severe oral mucositis, any oral mucositis, oral mucositis duration and onset, hematologic toxicity, diarrhea, broader gastrointestinal toxicity, renal toxicity, pain, severe broad toxicity, and a broad toxicity-prevention pool. The hierarchy progressed from outcome- and setting-specific pools to wider toxicity-domain pools and finally to cross-domain consistency checks. At least two independent clinically compatible studies were required for pooling. OR-only, model-based, median/range, and otherwise incompatible outcomes were displayed separately.

The primary analyses used DerSimonian-Laird random-effects models for pools with at least three compatible studies and fixed-effect inverse-variance models for protocol-compatible two-study pools (13). Heterogeneity was summarized with Cochran’s Q, τ^2^ where estimable, and I^2^ (14). Sensitivity analyses excluded studies judged to be High risk of bias and restricted comparisons to placebo-controlled trials when at least two studies remained.

Because conventional random-effects intervals can be unstable when few studies are available, every pooled RR and MD outcome was re-estimated with a restricted maximum-likelihood (REML) random-effects model and a Hartung-Knapp confidence interval (CI) (15, 16). Prediction intervals were reported descriptively for analyses with at least three studies. A result was considered model-sensitive when the primary-model confidence interval excluded the null, but the Hartung-Knapp interval did not. Fixed-effect or DerSimonian-Laird estimates were reported as the primary numerical analyses, with REML/Hartung-Knapp results used to evaluate robustness. Analyses were performed in R 4.3.2 with the metafor package version 5.0-1 (16).

Orvain 2025 was available only as a conference abstract and was excluded from full-report pools (17). Its arm-level severe oral mucositis, diarrhea, and all-adverse-event counts were added only in conference-augmented supplementary sensitivity analyses. A separate derived exploratory OR sensitivity combined Bahador 2022 with Muecke 2010 for grade 2–3 vs. grade 0 diarrhea. The standard error of the Bahador log OR was approximated from its rounded two-sided *p* value, and the Muecke OR was reconstructed from exact week-4 category counts. This analysis was oriented as no selenium vs. selenium and was never combined with RR estimates.

### 2.8 Reporting bias and certainty of evidence

Funnel plots and regression-based small-study-effect tests were not performed for the primary or outcome-specific exploratory pools because each contained fewer than 10 studies. The broad toxicity-prevention pool contained 11 studies, but its deliberate combination of clinically distinct toxicity domains made a formal asymmetry test difficult to interpret. Reporting bias was therefore considered narratively and within the Grading of Recommendations Assessment, Development and Evaluation (GRADE) publication-bias domain.

Certainty was assessed for all 13 pooled outcomes using GRADE across risk of bias, inconsistency, indirectness, imprecision, and publication bias (18). Conference-augmented and derived-OR sensitivities were not included among the pooled outcomes assessed with GRADE. The outcome-level evidence profile is presented in Supplementary Table S11.

## 3 Results

### 3.1 Study selection

The database searches identified 444 records: 113 from PubMed, 229 from Web of Science, 99 from CENTRAL, and 3 from Cochrane Reviews. After removal of 111 duplicates, 333 unique records were screened, and 287 were excluded by title and abstract. Forty-six database reports and three reports identified by supplemental methods were sought for retrieval. Eight reports could not be retrieved, leaving 41 reports for full-text assessment. Ten full-text reports were excluded. Thirty-one reports representing 30 independent study groups were included in the systematic review; two reports described one study group. Seventeen study groups contributed to the main analysis and 13 to supplementary exploratory evidence (Figure 1). Unretrieved reports are retained in the accounting as access limitations.

**Figure 1:**
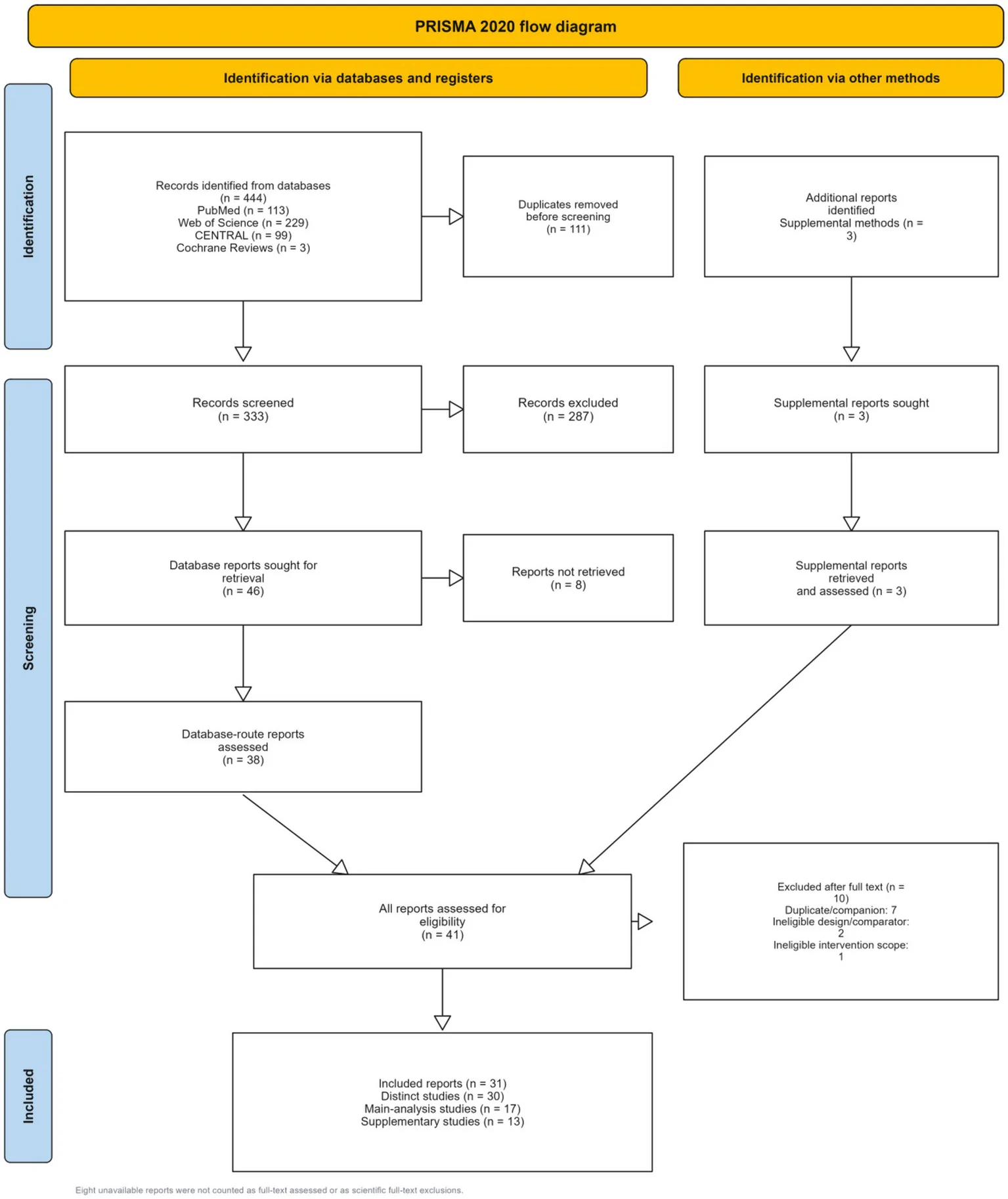
Study identification and selection. PRISMA 2020 flow diagram showing database and supplemental identification routes, deduplication, title and abstract screening, retrieval, full-text assessment, exclusions, included reports, distinct study groups, and main vs. supplementary evidence roles. Reports that could not be retrieved are shown as access limitations rather than scientific full-text exclusions.

### 3.2 Study and intervention characteristics

The 17 main-analysis studies were published from 1997 to 2026 and together reported 1,063 randomized or enrolled participants; study-level enrollment ranged from 16 to 133 (19–35). Designs included double-blind placebo-controlled randomized trials, randomized trials without placebo controls, crossover or cycle-based comparisons, and small phase II or pilot trials. Treatment settings included head-and-neck or pelvic radiotherapy and chemoradiotherapy, platinum-containing chemotherapy, paclitaxel-carboplatin-bevacizumab chemotherapy, high-dose chemotherapy followed by allogeneic transplantation, radioiodine therapy, pediatric chemotherapy, and cyclophosphamide, doxorubicin, vincristine, and prednisone (CHOP) chemotherapy.

Selenium exposure varied markedly. Regimens included intravenous sodium selenite pentahydrate, oral sodium selenite, selenium yeast, L-selenomethionine, selenium-glycine, seleno-kappacarrageenan, and other oral formulations. Doses, schedules, treatment durations, and comparator structures also differed. Table 1 summarizes the study populations, interventions, and comparators; Supplementary Table S3 provides participant and baseline characteristics.

**Table 1:** Included study characteristics.

Panel A. Design, setting, population, and sample.								
Study	Year	Country or region	Setting	Study design	Recruitment period	Population	Sample size	Age
Yim, 2026	2026	Republic of Korea	Single center; Dongguk/Seoul National University affiliated centers reported	Phase 3 double-blind parallel-group randomized placebo-controlled pilot trial	Enrollment period not reported	Platinum-sensitive recurrent ovarian/fallopian/peritoneal cancer	Randomized/enrolled: 68; analyzed: 60 PP for efficacy; 68 ITT for adverse events	PP Table 2: ≥60 years 18/30 selenium vs. 15/30 placebo
Yang, 2023	2023	China	Central Hospital of Enshi Tujia and Miao Autonomous Prefecture and collaborating Chinese centers	Randomized double-blind placebo-controlled parallel phase II clinical trial	July 2018 to May 2021	FIGO IIB cervical cancer receiving first-line CCRT	Randomized/enrolled: 124; analyzed: 104	Baseline characteristics were reported as similar
Laali, 2020	2020	Iran	Imam Khomeini Hospital Complex, Tehran	Double-blind placebo-controlled randomized clinical trial	February 2015 to March 2017	Head and neck cancer patients receiving RT to at least two-thirds of the oral cavity	Randomized/enrolled: 84 recruited; analyzed: 71	Mean age 52.14 selenium vs. 54.74 placebo; median 51 vs. 57.5
Mix, 2015	2015	United States/New Zealand	Roswell Park Cancer Institute and New Zealand site	International randomized double-blind phase II placebo-controlled trial	Recruitment stopped after a planned interim review	Stage III-IV HNSCC treated with concurrent CRT	Randomized/enrolled: 18; analyzed: 18	Median age 59.5 SLM vs. 55.5 placebo
Vieira, 2015	2015	Brazil	Pediatric oncology setting; institution not clearly reported	Randomized double-blind crossover study	September 2010 to January 2012	Children/adolescents with leukemia/lymphoma or solid tumors receiving chemotherapy	Randomized/enrolled: 39 included after 74 invited; analyzed: 39	Average age 8.2 ± 4.4 leukemia/lymphoma; 7.4 ± 4.3 solid tumors
Jahangard-Rafsanjani, 2013	2013	Iran	Shariati Hospital, Tehran University of Medical Sciences	Double-blind randomized placebo-controlled study	June 2011 to July 2012	Adults with AML or ALL undergoing allogeneic HSCT	Randomized/enrolled: 77; analyzed: 74 completed; tables report 37 per group	Mean age 33.3 selenium vs. 34.0 control; median 32 vs. 32
Ghorbani, 2013	2013	Iran	Ahvaz Jundishapur University of Medical Sciences affiliated setting	Double-blind randomized controlled clinical trial	Not reported	Cancer patients receiving cisplatin-containing chemotherapy	Randomized/enrolled: 133 enrolled; 11 lost to follow-up; analyzed: 122	44.77 years selenium vs. 46.37 years placebo
Muecke, 2010	2010	Germany	Ten German radiotherapy departments	Multicenter randomized phase 3 trial	January 2000 to June 2006	Selenium-deficient cervical/corpus uteri cancer patients after curative surgery receiving pelvic RT	Randomized/enrolled: 81; analyzed: 81	Median age 64.8 selenium vs. 63.8 control
Buentzel, 2010	2010	Germany	Six German centers	Small randomized phase II supportive-care study	2001 to 2007	Selenium-deficient head and neck SCC patients receiving RT	Randomized/enrolled: 40 randomized; analyzed: 39	Median 62.8 selenium vs. 63.5 control
Asfour, 2006	2006	Egypt	Cairo University Hospital	Randomized clinical trial with chemotherapy control; mechanistic toxicity-support endpoints	Not reported	Newly diagnosed intermediate/high-grade non-Hodgkin lymphoma	Randomized/enrolled: 30; analyzed: 30	Not reported
Hu, 1997	1997	China	Chinese oncology setting; hospital not clearly reported	Randomized crossover comparative clinical study	Not reported	Mixed cancer patients receiving cisplatin-containing chemotherapy	Randomized/enrolled: 41; analyzed: 41 patients/cycles per Se and control condition	Age 31–72
Ameri, 2016	2016	Iran	Kerman/Tehran affiliated Iranian setting	Pilot double-blind randomized placebo-controlled trial	Recruitment period not reported	Head and neck cancer patients receiving radiotherapy	Randomized/enrolled: 35 enrolled; analyzed: 30 (15 per arm)	Not clearly reported
Chen, 2023	2023	China	Anhui No. 2 Provincial People’s Hospital	Randomized clinical trial	Not reported	Patients with malignant tumors receiving platinum-based combination therapy	Randomized/enrolled: 86; analyzed: 86	Observation 60.89 ± 11.72 years; control 61.28 ± 9.34 years
Wang, 2020	2020	China	Second Affiliated Hospital of Hunan Normal University	Prospective randomized clinical study; random-number table allocation	October 2017 to October 2018	Initial treatment of nasopharyngeal carcinoma patients undergoing concurrent chemoradiotherapy	Randomized/enrolled: 60; analyzed: 60	Selenium 55.3 ± 10.4 years; routine 52.3 ± 10.5 years
Son, 2017	2017	Republic of Korea	Catholic Kwandong University International St. Mary’s Hospital	Single-blind randomized prospective study	April 2015 to May 2016	Differentiated thyroid cancer after total thyroidectomy before first high-dose I-131 therapy	Randomized/enrolled: 16; analyzed: 16	Median (range): selenium 40 (20–67), control 50 (36–65) years
Bahador, 2022	2022	Iran	Kerman University of Medical Sciences radiation oncology setting	Parallel interventional randomized study; block randomization	September 2017 to September 2018	Pelvic cancer patients undergoing chemoradiotherapy	Randomized/enrolled: 80; analyzed: 80	Mean ± SD: Se 59.5 ± 12.8; control 63.8 ± 11.2 years
Pavlidis, 2026	2026	Greece/Germany	General Hospital Papanikolaou and General Hospital Veria; University Medical Center Mainz affiliations	Randomized controlled comparative study with three arms	Not reported	Head and neck cancer patients receiving radiation involving at least two-thirds of the oral cavity	Randomized/enrolled: 51; analyzed: 51	Control 57.93 ± 3.7; Se 55.56 ± 3.42 years; Zn group also present

Panel B. Intervention, comparator, outcomes, follow-up, and provenance							
Study	Sex	Intervention or exposure	Comparator	Outcome domains	Follow-up	Adjustment or matching factors	Funding or conflict notes
Yim 2026	Female cancer population	Sodium selenite pentahydrate; intravenous; 2,000 μg/40 mL; 2 h before chemotherapy at each cycle; six chemotherapy cycles	40 mL intravenous normal saline placebo over the same schedule	CIPN	Total follow-up 29.8 ± 13.3 months selenium vs. 31.5 ± 11.3 months placebo	Unadjusted arm-level comparison; adjusted estimates were retained separately from RR pools.	Korean public research grants; investigational products supported by Boryung; no competing interests declared.
Yang 2023	Female cervical cancer population	Selenium yeast tablets; Oral; 100 μg selenium twice daily; During concurrent chemoradiotherapy; 5 weeks total	Matching placebo twice daily	Hematologic toxicity	During concurrent chemoradiotherapy; longer survival follow-up planned	Unadjusted arm-level comparison; adjusted estimates were retained separately from RR pools.	Chinese national, provincial, and local public grants; no commercial or financial conflict declared.
Laali 2020	Male 25/37 selenium vs. 25/34 placebo	Selenium tablets (Webber Naturals); Oral; 200 μg twice daily (400 μg/day); From first day of radiation including non-radiation days; until end of radiotherapy	Placebo tablets	Oral mucositis	Weekly during radiotherapy and one month after radiotherapy	Unadjusted arm-level comparison; adjusted estimates were retained separately from RR pools.	No specific public, commercial, or not-for-profit grant reported.
Mix 2015	Male 10/10 SLM vs. 7/8 placebo	L-selenomethionine; Oral capsules or tube-feed administration if needed; Closest capsule equivalent to 3,600 μg/m^2^; twice daily for 7 days before CRT, once daily during CRT; Started 7 days before CRT; continued during CRT; Until 3 weeks after completion of CRT	Matching placebo capsules	Oral mucositis	During CRT and 1-year follow-up for QoL/survival; trial stopped early	Unadjusted arm-level comparison; adjusted estimates were retained separately from RR pools.	Recruitment stopped because of funding constraints; authors reported no conflicts of interest.
Vieira 2015	Mixed male/female population; exact totals not reported	Selenium-glycine; Oral capsules; Age-adjusted daily dose reported as 27, 36, 54, 72, or 100 μg; Crossover periods during chemotherapy follow-up; 30-day selenium/placebo crossover periods; 1-year follow-up supplementation	Glycine-starch placebo capsules	Supportive symptoms/QoL	30-day selenium/placebo periods plus 1-year supplementation follow-up	Unadjusted arm-level comparison; adjusted estimates were retained separately from RR pools.	Volunteer-association support acknowledged; no competing financial interests declared.
Jahangard-Rafsanjani 2013	Male 21/37 in both groups	Selenium tablet (Webber Naturals); Oral; 200 μg twice daily (400 μg/day); from the starting day of high-dose chemotherapy; to 14 days after transplantation	Placebo tablet twice daily	Oral mucositis	Daily OM assessment to day 21 post-transplant or resolution; 3-month mortality	Unadjusted arm-level comparison; adjusted estimates were retained separately from RR pools.	No conflict of interest declared; funding source not identified in available report.
Ghorbani 2013	Female 18/61 selenium vs. 19/61 placebo	Selenium tablet; oral; single 400 μg tablet; the day before chemotherapy; single dose	Placebo tablet identical in color, weight, shape, and taste	Nephrotoxicity	Creatinine/BUN repeated on day 5 after cisplatin	Unadjusted arm-level comparison; adjusted estimates were retained separately from RR pools.	Ahvaz Jundishapur University of Medical Sciences grant U88342; no competing interests declared.
Muecke 2010	Female gynecologic cancer population	Sodium selenite (selenase); Oral; 500 μg on RT days and 300 μg on non-RT days; during adjuvant pelvic radiotherapy; until the last day of RT	Observation/no selenium supplementation	Radiation-induced diarrhea	During RT; 6 weeks after RT; median 49 months DFS and 51 months OS	Unadjusted arm-level comparison; adjusted estimates were retained separately from RR pools.	Supported by biosyn Arzneimittel GmbH; no conflict of interest declared.
Buentzel 2010	Not reported	Sodium selenite oral fluid; oral fluid; 500 μg two days before RT and on RT days; 300 μg on weekends/holidays; 1 h before RT on treatment days; during radiotherapy	Standardized RT without selenium or other cytoprotector	Head-and-neck radiation toxicity	Weekly during RT and 6 weeks after RT	Unadjusted arm-level comparison; adjusted estimates were retained separately from RR pools.	Funding and conflict-of-interest information not reported.
Asfour 2006	14 males and 16 females overall	Sodium selenite; oral; 0.2 mg Se/kg/day; Days 3–7 of chemotherapy cycle after doxorubicin clearance; 5 days	CHOP chemotherapy alone	Infection/cardiac/immune toxicity	Early post-chemotherapy apoptosis/infection/cardiac function assessment	Unadjusted arm-level comparison; adjusted estimates were retained separately from RR pools.	Funding and conflict-of-interest information not reported.
Hu 1997	Male: female 27:14	Seleno-kappa carrageenan; oral; 4,000 μg/day pure selenium equivalent divided four times daily; from 4 days before chemotherapy to 4 days after chemotherapy; 8 days around chemotherapy in selenium cycle	Same patient/cycle without selenium	Cisplatin toxicity	Urine enzymes through 72 h and blood counts through day 20 after chemotherapy	Unadjusted arm-level comparison; adjusted estimates were retained separately from RR pools.	Chinese Ministry of Public Health young-scientist grant; selenium product supplied by Beijing Tian Ci Fu Medical Biology Company.
Ameri 2016	Mixed male and female population; exact totals not clearly reported	Selenium; oral; 400 μg/day; started 1 day before RT; continued for 3 weeks after the end of RT	Placebo	Oral mucositis	Weekly during RT; intervention through 3 weeks after RT	Unadjusted arm-level comparison; adjusted estimates were retained separately from RR pools.	Funding and conflict-of-interest information not reported.
Chen 2023	Observation: 26 male/17 female; control: 23 male/20 female	Selenium yeast tablets; oral; 200 μg once daily; during platinum-containing combined regimen; 1 month	Platinum-containing combined regimen without selenium yeast	Broad adverse reactions; Gastrointestinal toxicity; Hematologic toxicity; Pain	1 month	Unadjusted arm-level comparison; adjusted estimates were retained separately from RR pools.	No specific grant; no conflict of interest declared.
Wang 2020	Selenium 16 male/14 female; routine 17 male/13 female	Selenium yeast tablets; oral; 200 μg once daily; from start of concurrent chemoradiotherapy; until end of concurrent chemoradiotherapy	Routine treatment without selenium yeast; both arms received Kangfuxin solution	Oral mucositis; Pain	Through end of concurrent chemoradiotherapy and short-term response assessment	Unadjusted arm-level comparison; adjusted estimates were retained separately from RR pools.	Funding and conflict-of-interest information not reported.
Son 2017	Selenium 3 male/5 female; control 3 male/5 female	Inorganic sodium selenite (selenase); oral; 300 μg/day; from 3 days before to 6 days after I-131 treatment; 10 days	Oral placebo for the same duration	Salivary gland injury	6 months	Unadjusted arm-level comparison; adjusted estimates were retained separately from RR pools.	Catholic Kwandong University research fund CKURF-201406580001; no conflicts declared.
Bahador 2022	Se 20 male/20 female; control 28 male/12 female	Oral selenium solution (salt not clearly specified); 200 μg/day; 1 h before radiotherapy; continued throughout treatment	Distilled water placebo without selenium intake	Gastrointestinal toxicity; Other toxicity	During treatment and weeks after radiotherapy	Unadjusted arm-level comparison; adjusted estimates were retained separately from RR pools.	No financial interest or author conflict declared; funding source not identified in available report.
Pavlidis 2026	Control 14 male/3 female; Se paragraph reports 13 male/3 female despite 17 per group in abstract	Selenium tablets; oral; 200 μg twice daily (400 μg/day); from the first day of radiochemotherapy until the end of treatment; During radiation/radiochemotherapy	No supplement control; zinc arm also present but not the target comparator	Oral mucosa/taste function	Through 1 month after the second chemotherapy cycle	Unadjusted arm-level comparison; adjusted estimates were retained separately from RR pools.	Open-access funding through Projekt DEAL; authors reported no financial support.

RR pools used arm-level comparisons; adjusted estimates were retained separately. RT, radiotherapy; CRT, chemoradiotherapy; NR, not reported; ALL, acute lymphoblastic leukemia; AML, acute myeloid leukemia; BUN, blood urea nitrogen; CCRT, concurrent chemoradiotherapy; CHOP, cyclophosphamide, doxorubicin, vincristine, and prednisone; CIPN, chemotherapy-induced peripheral neuropathy; DFS, disease-free survival; FIGO, International Federation of Gynecology and Obstetrics; HNSCC, head and neck squamous cell carcinoma; HSCT, hematopoietic stem cell transplantation; ITT, intention-to-treat; OM, oral mucositis; OS, overall survival; PP, per-protocol; QoL, quality of life; RR, risk ratio; SCC, squamous cell carcinoma; SD, standard deviation; Se, selenium; SLM, L-selenomethionine; Zn, zinc.

### 3.3 Risk of bias and certainty

Overall risk of bias was judged to be Some concerns for 9 studies and High risk of bias for 8; no main-analysis study was judged Low risk overall. Recurrent concerns involved allocation concealment, non-placebo or open-label comparators, attrition or per-protocol analysis, early stopping, crossover or cycle-level designs, translation-sensitive reporting, and unclear prespecification (Supplementary Table S5; Supplementary Figures S12 and S13).

Across the 13 pooled outcomes, GRADE certainty was Low for 3 and Very low for 10. The primary severe oral mucositis outcome was rated Very low because of risk-of-bias concerns, inconsistency, imprecision, and sensitivity to comparator restrictions. No outcome was rated Moderate or High certainty (Supplementary Table S11).

### 3.4 Primary severe oral mucositis analysis

Four head-and-neck radiotherapy or chemoradiotherapy studies reported compatible severe oral mucositis or stomatitis outcomes: Laali 2020, Mix 2015, Buentzel 2010, and Wang 2020 (22, 23, 28, 31). Across 188 participants, the primary random-effects analysis was inconclusive (RR 0.89, 95% CI 0.50 to 1.56; *p* = 0.679; I^2^ 44.9%; Figure 2; Table 2). The REML/Hartung-Knapp result was similarly inconclusive (RR 0.88, 95% CI 0.35 to 2.23), with a prediction interval of 0.18 to 4.30.

**Figure 2:**
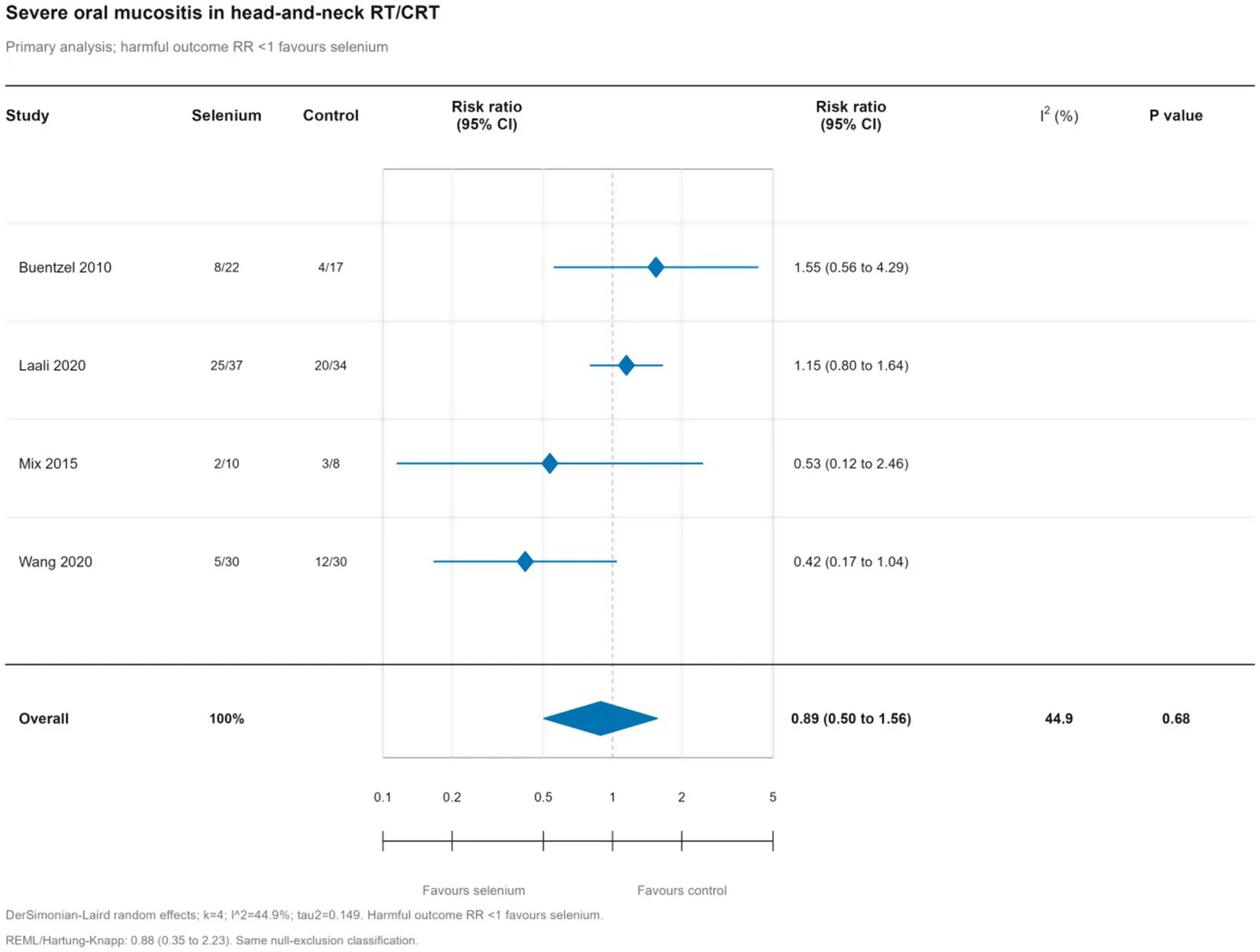
Selenium supplementation and severe oral mucositis during head-and-neck radiotherapy or chemoradiotherapy. Study-level and pooled RRs are oriented as selenium vs. control; values below 1 favor selenium for this harmful outcome. The diamond shows the primary DerSimonian-Laird random-effects estimate. The REML/Hartung-Knapp robustness estimate and final risk-of-bias judgments are reported in the figure.

**Table 2:** Primary and exploratory meta-analysis results.

Panel A. Primary-model estimates and heterogeneity.									
Outcome	Analysis role	Studies	Participants or events	Effect measure	Model	Pooled estimate	95% CI or CrI	*p* value	Heterogeneity statistic
Severe oral mucositis, head-and-neck RT/CRT	Primary	4	N = 188; events 40/99 (selenium) and 39/89 (control)	RR	DerSimonian-Laird random effects	0.89	0.50 to 1.56	0.679	I^2^ = 44.9%; Q(3) = 5.45, *p* = 0.142; τ^2^ = 0.149
Severe oral mucositis, expanded contexts	Exploratory	5	N = 262; events 44/136 (selenium) and 52/126 (control)	RR	DerSimonian-Laird random effects	0.71	0.38 to 1.33	0.287	I^2^ = 60.9%; Q(4) = 10.24, *p* = 0.037; τ^2^ = 0.287
Any oral mucositis	Exploratory	2	N = 145; events 67/74 (selenium) and 64/71 (control)	RR	Fixed-effect inverse variance (two-study protocol-compatible)	0.98	0.91 to 1.05	0.599	I^2^ = 0.0%; Q(1) = 0.26, *p* = 0.607
Grade 3 hematologic toxicity	Exploratory	2	N = 138; events 26/68 (selenium) and 40/70 (control)	RR	Fixed-effect inverse variance (two-study protocol-compatible)	0.70	0.50 to 0.97	0.033	I^2^ = 0.0%; Q(1) = 0.04, *p* = 0.846
Expanded hematologic toxicity	Exploratory	4	N = 242; events 30/121 (selenium) and 46/121 (control)	RR	DerSimonian-Laird random effects	0.70	0.50 to 0.96	0.028	I^2^ = 0.0%; Q(3) = 1.40, *p* = 0.706; τ^2^ = 0.000
Diarrhea event	Exploratory	2	N = 185; events 17/89 (selenium) and 29/96 (control)	RR	Fixed-effect inverse variance (two-study protocol-compatible)	0.60	0.37 to 0.98	0.040	I^2^ = 47.3%; Q(1) = 1.90, *p* = 0.168
Gastrointestinal toxicity or diarrhea	Exploratory	3	N = 271; events 30/132 (selenium) and 50/139 (control)	RR	DerSimonian-Laird random effects	0.61	0.42 to 0.88	0.007	I^2^ = 0.0%; Q(2) = 1.91, *p* = 0.385; τ^2^ = 0.000
Renal toxicity event	Exploratory	2	N = 196; events 3/98 (selenium) and 14/98 (control)	RR	Fixed-effect inverse-variance (two-study protocol-compatible)	0.31	0.10 to 1.00	0.051	I^2^ = 27.1%; Q(1) = 1.37, *p* = 0.241
Pain grade ≥ 2	Exploratory	2	N = 146; events 41/73 (selenium) and 56/73 (control)	RR	Fixed-effect inverse variance (two-study protocol-compatible)	0.78	0.64 to 0.95	0.015	I^2^ = 31.6%; Q(1) = 1.46, *p* = 0.227
Severe broad toxicity	Exploratory	7	N = 452; events 68/229 (selenium) and 92/223 (control)	RR	DerSimonian-Laird random effects	0.71	0.46 to 1.09	0.116	I^2^ = 53.8%; Q(6) = 12.98, *p* = 0.043; τ^2^ = 0.138
Broad toxicity-prevention pool	Exploratory	11	N = 745; events 99/374 (selenium) and 163/371 (control)	RR	DerSimonian-Laird random effects	0.60	0.42 to 0.83	0.003	I^2^ = 55.5%; Q(10) = 22.48, *p* = 0.013; τ^2^ = 0.140
Oral mucositis duration in days	Scope-sensitive exploratory	2	N = 145	MD	Fixed-effect inverse variance (two-study protocol-compatible)	-1.72	-2.64 to −0.80	<0.001	I^2^ = 0.0%; Q(1) = 0.15, *p* = 0.695
Oral mucositis onset in days	Exploratory	2	N = 131	MD	Fixed-effect inverse variance (two-study protocol-compatible)	0.98	-0.44 to 2.39	0.178	I^2^ = 23.2%; Q(1) = 1.30, *p* = 0.254

Panel B. Prediction intervals, certainty, robustness, and figure links			
Outcome	Prediction interval if used	Certainty or caveat	Figure or supplement link
Severe oral mucositis, head-and-neck RT/CRT	REML prediction interval 0.18 to 4.30; interpret cautiously with small k	GRADE Very low; REML/Hartung-Knapp RR 0.88 (0.35 to 2.23). Primary estimate remains inconclusive.	Figure 2
Severe oral mucositis, expanded contexts	REML prediction interval 0.13 to 3.96; interpret cautiously with small k	GRADE Very low; REML/Hartung-Knapp RR 0.71 (0.30 to 1.67). Exploratory result; not a confirmatory efficacy conclusion.	Supplementary Figure S1
Any oral mucositis	Not calculated for the fixed-effect primary analysis	GRADE Low; REML/Hartung-Knapp RR 0.98 (0.77 to 1.24). Exploratory result; not a confirmatory efficacy conclusion.	Supplementary Figure S2
Grade 3 hematologic toxicity	Not calculated for the fixed-effect primary analysis	GRADE Low; REML/Hartung-Knapp RR 0.70 (0.46 to 1.06). The primary-model confidence interval excluded the null, whereas the Hartung-Knapp interval did not; model-sensitive.	Supplementary Figure S3
Expanded hematologic toxicity	REML prediction interval 0.49 to 1.00; interpret cautiously with small k	GRADE Very low; REML/Hartung-Knapp RR 0.70 (0.49 to 1.00). Exploratory result; not a confirmatory efficacy conclusion.	Supplementary Figure S4
Diarrhea event	Not calculated for the fixed-effect primary analysis	GRADE Very low; REML/Hartung-Knapp RR 0.70 (0.00 to 204.19). The primary-model confidence interval excluded the null, whereas the Hartung-Knapp interval did not; model-sensitive.	Supplementary Figure S5
Gastrointestinal toxicity or diarrhea	REML prediction interval 0.28 to 1.33; interpret cautiously with small k	GRADE Very low; REML/Hartung-Knapp RR 0.61 (0.28 to 1.33). The primary-model confidence interval excluded the null, whereas the Hartung-Knapp interval did not; model-sensitive.	Supplementary Figure S6
Renal toxicity event	Not calculated for the fixed-effect primary analysis	GRADE Very low; REML/Hartung-Knapp RR 0.27 (8.59e-06 to 8202.54). Exploratory result; not a confirmatory efficacy conclusion.	Supplementary Figure S7
Pain grade ≥ 2	Not calculated for the fixed-effect primary analysis	GRADE Low; REML/Hartung-Knapp RR 0.74 (0.09 to 5.98). The primary-model confidence interval excluded the null, whereas the Hartung-Knapp interval did not; model-sensitive.	Supplementary Figure S8
Severe broad toxicity	REML prediction interval 0.22 to 2.21; interpret cautiously with small k	GRADE Very low; REML/Hartung-Knapp RR 0.70 (0.40 to 1.23). Exploratory result; not a confirmatory efficacy conclusion.	Supplementary Figure S9
Broad toxicity-prevention pool	REML prediction interval 0.24 to 1.50; interpret cautiously with small k	GRADE Very low; REML/Hartung-Knapp RR 0.60 (0.40 to 0.89). Exploratory result; not a confirmatory efficacy conclusion.	Supplementary Figure S10
Oral mucositis duration in days	Not calculated for the fixed-effect primary analysis	GRADE Very low; REML/Hartung-Knapp MD -1.72 (−4.06 to 0.62). The primary-model confidence interval excluded the null, whereas the Hartung-Knapp interval did not; model-sensitive.	Supplementary Figure S11
Oral mucositis onset in days	Not calculated for the fixed-effect primary analysis	GRADE Very low; REML/Hartung-Knapp MD 0.84 (−10.70 to 12.38). Exploratory result; not a confirmatory efficacy conclusion.	Supplementary Figure S14

The plotted estimates correspond to the primary fixed-effect or DerSimonian-Laird models. REML/Hartung-Knapp results are robustness analyses. RR below 1 and MD below 0 favor selenium for harmful toxicity outcomes. I^2^, inconsistency statistic; CI, confidence interval; CrI, credible interval; CRT, chemoradiotherapy; GRADE, Grading of Recommendations Assessment, Development and Evaluation; k, number of studies; MD, mean difference; N, number of participants; Q, Cochran’s Q statistic; REML, restricted maximum likelihood; RR, risk ratio; RT, radiotherapy; τ^2^, between-study variance.

Excluding the study judged to be High risk of bias yielded RR 0.73 (95% CI 0.34 to 1.56; k = 3). Restriction to the two placebo-controlled trials yielded RR 1.10 (95% CI 0.78 to 1.57). Hartung-Knapp analyses of these subsets also crossed the null. Wang 2020 remained in the primary pool because both groups received Kangfuxin solution and the randomized contrast isolated add-on selenium, but the absence of a selenium-matched placebo was addressed in the placebo-controlled sensitivity analysis (31).

### 3.5 Oral mucositis exploratory analyses

Adding the transplantation setting from Jahangard-Rafsanjani 2013 produced an expanded severe oral mucositis pool of 5 studies (RR 0.71, 95% CI 0.38 to 1.33; I^2^ 60.9%) (25). Any oral mucositis showed no clear difference in a two-study analysis (RR 0.98, 95% CI 0.91 to 1.05; I^2^ 0%). These estimates remained inconclusive under REML/Hartung-Knapp analyses.

Oral mucositis duration was shorter in the primary two-study fixed-effect analysis (MD −1.72 days, 95% CI −2.64 to −0.80; I^2^ 0%), but the Hartung-Knapp interval crossed the null (MD −1.72 days, 95% CI −4.06 to 0.62). This scope-sensitive outcome was therefore considered model-sensitive rather than evidence of a robust duration benefit (Supplementary Figure S11).

Oral mucositis onset was available from Laali 2020 and Wang 2020 after conversion of the Laali estimates from weeks to days. The fixed-effect estimate was inconclusive (MD 0.98 days, 95% CI −0.44 to 2.39; I^2^ 23.2%). The REML/Hartung-Knapp estimate was also inconclusive (MD 0.84 days, 95% CI −10.70 to 12.38). Positive values indicated later onset with selenium (Supplementary Figure S14).

### 3.6 Other exploratory toxicity domains

The primary analysis of grade 3 hematologic toxicity suggested a lower risk with selenium (RR 0.70, 95% CI 0.50 to 0.97; k = 2), but the Hartung-Knapp interval included the null (RR 0.70, 95% CI 0.46 to 1.06). The expanded four-study hematologic analysis also favored selenium (RR 0.70, 95% CI 0.50 to 0.96; I^2^ 0%); its REML/Hartung-Knapp interval was 0.49 to 1.00. The expanded analysis incorporated different hematologic definitions and data from Chen 2023, whose reported event counts were usable but whose Table V contained an inconsistent percentage (20).

Two primary analyses of gastrointestinal outcomes excluded the null: diarrhea (RR 0.60, 95% CI 0.37 to 0.98; k = 2; I^2^ 47.3%) and broader gastrointestinal toxicity or diarrhea (RR 0.61, 95% CI 0.42 to 0.88; k = 3; I^2^ 0%). Their Hartung-Knapp intervals were wide and crossed the null: RR 0.70 (95% CI 0.002 to 204.19) and RR 0.61 (95% CI 0.28 to 1.33), respectively. Differences in toxicity definitions and treatment settings further limited interpretation.

The renal toxicity analysis was inconclusive (RR 0.31, 95% CI 0.10 to 1.00; k = 2; I^2^ 27.1%). Pain grade 2 or higher favored selenium in the primary two-study analysis (RR 0.78, 95% CI 0.64 to 0.95; I^2^ 31.6%) but not under the Hartung-Knapp analysis (RR 0.74, 95% CI 0.09 to 5.98). The severe broad-toxicity analysis was also inconclusive (RR 0.71, 95% CI 0.46 to 1.09; k = 7; I^2^ 53.8%).

The broad toxicity-prevention pool included 11 study-domain contributions and excluded the null under both the primary model (RR 0.60, 95% CI 0.42 to 0.83; I^2^ 55.5%) and REML/Hartung-Knapp analysis (RR 0.60, 95% CI 0.40 to 0.89). Its prediction interval crossed the null (0.24 to 1.50). Because this pool deliberately combined oral, hematologic, gastrointestinal, renal, infectious or immune, neuropathic, and other outcomes, it was interpreted as a cross-domain consistency check rather than evidence that selenium prevents a specific toxicity.

Conference-augmented sensitivities did not change the primary inference. Adding Orvain 2025 to the expanded severe oral mucositis analysis produced RR 0.83 (95% CI 0.56 to 1.23; k = 6), with a REML/Hartung-Knapp RR of 0.80 (95% CI 0.44 to 1.48) (17). The threshold-mixed diarrhea sensitivity gave RR 0.60 (95% CI 0.39 to 0.94; k = 3), but its Hartung-Knapp interval crossed the null (0.23 to 1.56). The conference-augmented broad toxicity estimate was RR 0.66 (95% CI 0.48 to 0.90; k = 12; I^2^ 73.2%). These analyses were reported as supplementary sensitivity analyses and were not included in the primary efficacy estimates (Supplementary Table S9; Supplementary Figures S15–S17).

### 3.7 OR-only, single-study, and narrative evidence

Bahador 2022 reported generalized estimating equation ORs for diarrhea and pain without arm-level event counts and was not combined with RR pools (34). The publication reported ORs of 3.6 for grade II/III diarrhea vs. grade 0, 2.3 for grade II/III diarrhea vs. grade I, 1.7 for grade I diarrhea vs. grade 0, and 0.56 for pain. A derived sensitivity combined the first contrast with a Muecke 2010 week-4 OR reconstructed from grade 2–3 and grade 0 counts. The fixed-effect pooled OR was 3.76 for no selenium vs. selenium (95% CI 1.78 to 7.94; k = 2). The result remained separate from all RR pools because the Bahador variance was approximated from a rounded *p* value and the Muecke estimate was unadjusted (Supplementary Table S9; Supplementary Figure S18).

Yim 2026 was the only direct selenium trial reporting chemotherapy-induced peripheral neuropathy (CIPN) and remained a single-study display (19). At 3 months after chemotherapy, grade 1 or higher neuropathy or paresthesia occurred in 19 of 26 selenium participants and 24 of 30 placebo participants. The CIPN20 supplement yielded a sensory-score MD of 5.78 (95% CI −5.54 to 17.10; n = 30 per arm). Ameri 2016 analyzed 15 participants per arm and reported lower mean mucositis scores with selenium at weeks 1–3, but no second trial used the same 14-site score. Son 2017 evaluated salivary-gland injury using median/range and within-arm scintigraphy summaries (32). Pavlidis 2026 reported model-based electrogustometry contrasts, including control-minus-selenium differences of 1.365 at location A and 0.918 at location B, but no compatible second trial was available (35). These outcomes remained single-study or narrative evidence.

### 3.8 Safety and supplementary evidence

The 13 supplementary exploratory study groups covered early-phase safety, pharmacodynamic, lymphedema-treatment, single-arm, multicomponent, and other contextual clinical evidence. They were not combined with comparative prevention estimates when selenium-specific attribution or a compatible control was absent. No pooled selenium-attributed adverse-event estimate was possible.

Within the main-analysis evidence, Yim 2026 reported any-grade adverse events in 7 of 34 selenium participants and 8 of 34 placebo participants and stated that no adverse event was attributed to selenium (19). This single trial cannot establish safety across selenium formulations or cancer-treatment settings. Supplementary Table S12 summarizes the wider safety and contextual evidence.

The synthesis routes and evidence density across toxicity domains are summarized in Figure 3.

**Figure 3:**
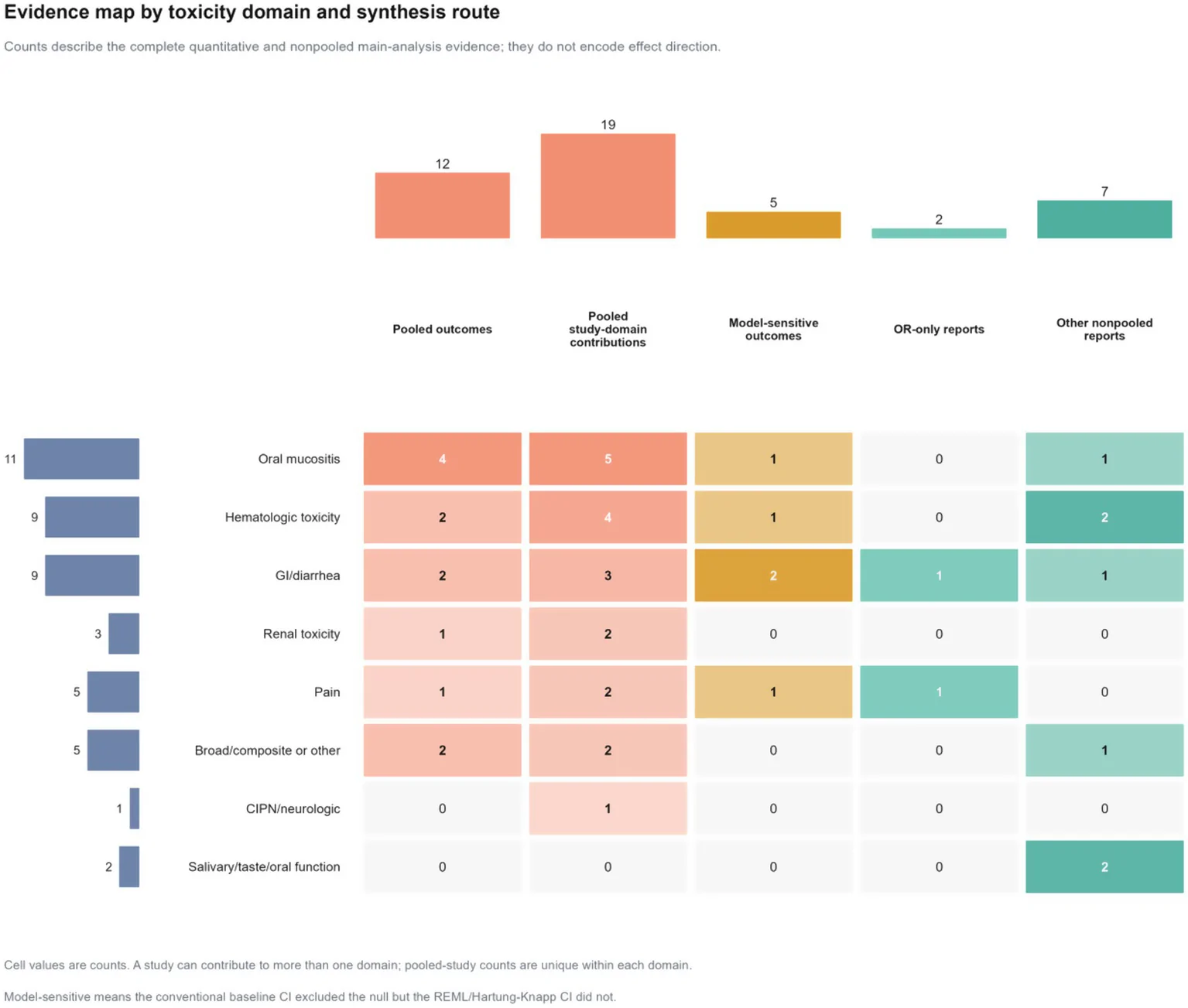
Evidence map by toxicity domain and synthesis route. Matrix showing the number of pooled outcomes, pooled study-domain contributions, model-sensitive outcomes, OR-only reports, and other non-pooled reports across toxicity domains. Counts describe evidence density and synthesis route and do not encode treatment benefit or harm.

## 4 Discussion

This review does not establish that selenium prevents severe oral mucositis during head-and-neck radiotherapy or chemoradiotherapy. The primary estimate was imprecise under both the primary and REML/Hartung-Knapp models, and neither exclusion of studies judged High risk nor restriction to placebo-controlled trials produced a clear benefit. With very low-certainty evidence, the estimate is compatible with meaningful benefit, no effect, and potential harm. At the same time, the direction of several domain-specific estimates and the stable cross-domain result indicate a toxicity-mitigation signal that warrants further targeted clinical evaluation.

The oral mucositis evidence illustrates why a layered synthesis was necessary. Severe mucositis in head-and-neck radiotherapy, severe mucositis after transplantation, any-grade mucositis, onset, and duration are related but not interchangeable. Combining them would increase data density at the cost of clinical interpretability. The narrow primary analysis therefore remains the clinical anchor, while expanded, onset, and duration analyses provide supporting context.

Several conventional exploratory estimates favored selenium in clinically relevant directions. The primary-model estimates suggested lower grade 3 hematologic toxicity (RR 0.70), diarrhea (RR 0.60), broader gastrointestinal toxicity (RR 0.61), and grade 2 or higher pain (RR 0.78), together with shorter oral mucositis duration (MD -1.72 days). These domains matter to clinical nutrition because gastrointestinal symptoms and mucosal injury can impair intake, while hematologic toxicity and pain can interrupt treatment and functional recovery (1, 2, 20, 22, 25, 27, 31). Five estimates, however, no longer excluded the null when uncertainty was recalculated with Hartung-Knapp intervals. The majority of pools contained only two or three studies, so the findings should be described as supportive toxicity-mitigation signals rather than confirmatory effects. The expanded hematologic estimate remained just below the null under Hartung-Knapp analysis, but its mixed definitions, the reported percentage inconsistency in the Chen data, and its very low-certainty rating still limit confidence.

The broad toxicity-prevention estimate was the most stable evidence of a potential mitigation effect: both the primary and REML/Hartung-Knapp models produced an RR of 0.60, and both confidence intervals excluded the null. This reproducible direction across analytic frameworks supports a genuine cross-domain hypothesis, especially when considered alongside the gastrointestinal, hematologic, pain, and mucositis-duration estimates. It is not proof that one selenium regimen prevents every toxicity, because the pool combines outcomes with different baseline risks, severity thresholds, mechanisms, and treatment contexts. The prediction interval crossing the null and the GRADE rating of Very low therefore limit clinical generalization and temper the interpretation of the observed consistency.

The observed direction is biologically plausible. Selenium-dependent glutathione peroxidases and thioredoxin reductases help regulate reactive oxygen species, redox signaling, and antioxidant defense, while other selenoproteins contribute to immune and inflammatory control (7–9). Maintaining these systems during radiotherapy or chemotherapy could reduce collateral injury in normal mucosa, gastrointestinal tissue, bone marrow, kidney, and peripheral nerves.

Formulation is a clinically meaningful source of heterogeneity rather than a dispensable intervention detail. The included trials evaluated inorganic sodium selenite, including oral fluid and intravenous sodium selenite pentahydrate; organic selenium yeast and L-selenomethionine; and less common oral selenium-glycine or seleno-kappacarrageenan preparations. These interventions should not be regarded as biologically equivalent merely because their labels report elemental selenium: organic and inorganic selenium species can differ in absorption, metabolic retention, and selenium-species distribution, while intravenous selenite bypasses intestinal absorption. Likewise, a selenium solution should not be equated with a defined selenium species when the selenium salt is not reported. However, the available evidence contains no direct head-to-head formulation comparisons and too few clinically compatible studies within any individual formulation to identify a superior selenium species, route, or dosage form (6, 36, 37).

Recent systematic reviews likewise regard the clinical evidence as heterogeneous: toxicity reductions may occur in selected settings, but survival or quality-of-life benefit is not consistently demonstrated in unselected populations (4, 5). This pattern gives a biologically and clinically rational basis for testing selenium as a normal-tissue protective adjunct in patients with documented selenium deficiency or a clearly prespecified, high-risk toxicity profile, provided baseline status and exposure are monitored. It does not establish benefit for every patient, formulation, or anticancer regimen.

Additional analyses clarified which data could be synthesized quantitatively. Harmonization of time units permitted pooling of oral mucositis onset, whereas conference-only data were included only in supplementary sensitivity analyses. The Bahador-Muecke OR could be calculated but relied on an approximate variance and a reconstructed contrast. Neuropathy, salivary-gland injury, oral mucosal function, and taste still lacked a compatible second study. Future reports should prioritize arm-level counts, means, standard deviations, prespecified thresholds, and analysis populations.

The effect of selenium on treatment-related toxicity is likely concentration- and formulation-dependent. In selenium-inadequate patients, repletion may restore selenium-dependent redox defenses; once nutritional adequacy is reached, further increases may not add cytoprotection, whereas pharmacological exposure, particularly to inorganic selenite, may become pro-oxidant (6, 38). This non-linear pattern was not directly tested in the included trials. In the only trial with serial whole-blood measurements, 200 μg/day selenium yeast increased selenium from 58.34 ± 17.63 to 76.16 ± 24.47 μg/L, compared with 57.48 ± 14.92 μg/L with placebo, and was associated with less grade 3 myelosuppression (48% vs. 63%) (21). However, this fixed-dose, single-formulation study cannot define 76.16 μg/L as a therapeutic target or show that higher concentrations yield greater protection. Therefore, no universal selenium target or dose can be recommended; future trials should stratify by baseline status, specify form and dose, monitor serial blood selenium and toxicity, and report selenium-attributable adverse events (39, 40).

The main strengths of this review are the explicit narrow-to-broad synthesis hierarchy, separation of RR, OR, MD, and non-pooled evidence, consolidation of companion reports, extraction linked to reported source locations, final risk-of-bias judgments, model-robustness analyses, and outcome-level GRADE assessment. These methods enabled presentation of the wider evidence base while preserving the clinically coherent primary question.

Several limitations remain. All 17 main-analysis studies received overall ratings of Some concerns or High risk, and many were small or had non-placebo, open-label, crossover, or translation-sensitive designs; some were stopped early. The search did not include Embase or regional bibliographic databases, and eight reports could not be retrieved. Chen 2023 contained a percentage inconsistency despite usable event counts, and Asfour 2006 contained a discrepancy between infection counts and its significance statement. Bahador 2022 lacked arm-level event counts and an exact variance, while the Orvain evidence was conference-only. The Muecke OR reconstruction excluded grade 1 diarrhea to match the available Bahador contrast. Expanded and cross-domain pools remained clinically heterogeneous. Statistical robustness cannot correct limitations in design, measurement, or reporting.

The most defensible clinical-nutrition implication is targeted evaluation rather than blanket supplementation. The most plausible population for such trials comprises patients with documented selenium deficiency or a high, prespecified risk of a treatment-related toxicity, because the current synthesis suggests potential toxicity reduction in selected settings but not a universal treatment effect (4, 5, 40). Future trials should use placebo-controlled designs where feasible and report the selenium compound, route, dose, timing, adherence, and selenium-attributed adverse events. Arm-level event counts and denominators should be reported for every relevant toxicity grade, and continuous outcomes should include arm-level means, standard deviations, time points, and analysis populations. These practices would determine whether the cross-domain signal can be converted into reliable, domain-specific supportive-care recommendations.

## 5 Conclusion

Current clinical-trial evidence does not establish that selenium supplementation prevents severe oral mucositis during head-and-neck radiotherapy or chemoradiotherapy. Nevertheless, the stable cross-domain RR, together with supportive estimates for gastrointestinal and hematologic toxicity, pain, and oral mucositis duration, suggests that selenium may exert clinically meaningful toxicity-mitigation effects in selected settings. The most defensible implication is not routine universal supplementation but targeted evaluation in selenium-deficient or high-risk patients using standardized compounds, doses, and domain-specific outcomes. Well-powered placebo-controlled trials are needed before practice recommendations can be made.

## Data Availability

The datasets presented in this study can be found in online repositories. The names of the repository/repositories and accession number(s) can be found in the article/Supplementary material.
