# Supplementary material for: Selenium supplementation for preventing cancer therapy-related toxicities: a systematic review and meta-analysis of clinical trials
Source: Front Nutr. 2026 Oct 7;13:1940806. doi: 10.3389/fnut.2026.1940806 (PMC13683797; doi:10.3389/fnut.2026.1940806)
Supplement: Supplementary file 1 [file Table_1.DOCX]

Supplementary Material

# 1 Supplementary Tables

**Supplementary Table S1. Complete database search strategies**

**Panel A. Search execution and exports**

| **Database** | **Platform** | **Date searched** | **Coverage dates** | **Limits** | **Records retrieved** | **Export file** | **Notes** |
| --- | --- | --- | --- | --- | --- | --- | --- |
| PubMed | NCBI E-utilities / PubMed | 2026-06-30 | All years to 2026-06-30 | NOT animals[mh] NOT humans[mh]; no date/language limits | 113 | PubMed XML and NBIB exports | Formal database export retained in full; no screening restrictions were applied at export. |
| Web of Science | Web of Science Core Collection | 2026-06-30 | All years to 2026-06-30 | All years; no language or document-type restrictions | 229 | Web of Science XLS and CSV exports | The complete Web of Science export was retained for reproducibility. |
| Cochrane Library | Cochrane Library / CENTRAL | 2026-06-30 | All years to 2026-06-30 | CENTRAL/trials category; all years; no language restrictions | 99 | Cochrane CENTRAL text export | Full CENTRAL export retained for reproducibility. |
| Cochrane Library | Cochrane Library / Cochrane Reviews | 2026-06-30 | All years to 2026-06-30 | Cochrane Reviews category; all years; no language restrictions | 3 | Cochrane Reviews CSV export | All review-category records were reconciled during deduplication against the other database exports. |

**Panel B. Exact search strings**

**PubMed**

**One-line query**

[1/3] (("Selenium"[Mesh] OR selenium[tiab] OR selenite[tiab] OR selenomethionine[tiab] OR "sodium selenite"[tiab] OR "selenium yeast"[tiab] OR selenious[tiab]) AND ("Neoplasms"[Mesh] OR cancer*[tiab] OR tumor*[tiab] OR tumour*[tiab] OR neoplasm*[tiab] OR carcinoma[tiab] OR lymphoma[tiab] OR leukemia[tiab] OR leukaemia[tiab] OR malignan*[tiab]) AND ("Radiotherapy"[Mesh] OR "Drug Therapy"[Mesh] OR "Antineoplastic Combined Chemotherapy Protocols"[Mesh] OR chemotherap*[tiab] OR radiotherap*[tiab] OR radiation[tiab] OR irradiat*[tiab] OR chemoradiotherap*[tiab] OR "radioactive iodine"[tiab] OR radioiodine[tiab] OR surgery[tiab] OR surgical[tiab] OR

[2/3] endocrine therap*[tiab] OR hormone therap*[tiab] OR platinum[tiab] OR paclitaxel[tiab] OR carboplatin[tiab] OR cisplatin[tiab]) AND ("Drug-Related Side Effects and Adverse Reactions"[Mesh] OR "Mucositis"[Mesh] OR "Diarrhea"[Mesh] OR "Lymphedema"[Mesh] OR toxicit*[tiab] OR adverse[tiab] OR "side effect"[tiab] OR "side effects"[tiab] OR mucositis[tiab] OR diarrhea[tiab] OR diarrhoea[tiab] OR myelosuppression[tiab] OR hematologic*[tiab] OR haematologic*[tiab] OR neutropenia[tiab] OR leukopenia[tiab] OR thrombocytopenia[tiab] OR nephrotox*[tiab] OR neuropath*[tiab] OR lymphedema[tiab] OR lymphoedema[tiab] OR dermatitis[tiab] OR

[3/3] radiodermatitis[tiab] OR xerostomia[tiab] OR dysphagia[tiab] OR erysipelas[tiab] OR infection*[tiab]) AND (clinical trial[pt] OR randomized controlled trial[pt] OR controlled clinical trial[pt] OR clinical trial[tiab] OR trial[tiab] OR randomized[tiab] OR randomised[tiab] OR placebo[tiab] OR double-blind[tiab] OR controlled[tiab])) NOT (animals[mh] NOT humans[mh])

**Structured search blocks**

[1/3] ("Selenium"[Mesh] OR selenium[tiab] OR selenite[tiab] OR selenomethionine[tiab] OR "sodium selenite"[tiab] OR "selenium yeast"[tiab] OR selenious[tiab]) AND ("Neoplasms"[Mesh] OR cancer*[tiab] OR tumor*[tiab] OR tumour*[tiab] OR neoplasm*[tiab] OR carcinoma*[tiab] OR lymphoma[tiab] OR leukemia[tiab] OR leukaemia[tiab] OR malignan*[tiab]) AND ("Radiotherapy"[Mesh] OR "Drug Therapy"[Mesh] OR "Antineoplastic Combined Chemotherapy Protocols"[Mesh] OR chemotherap*[tiab] OR radiotherap*[tiab] OR radiation[tiab] OR irradiat*[tiab] OR chemoradiotherap*[tiab] OR "radioactive iodine"[tiab] OR radioiodine[tiab] OR surgery[tiab] OR surgical[tiab] OR

[2/3] endocrine therap*[tiab] OR hormone therap*[tiab] OR platinum[tiab] OR paclitaxel[tiab] OR carboplatin[tiab] OR cisplatin[tiab]) AND ("Drug-Related Side Effects and Adverse Reactions"[Mesh] OR "Mucositis"[Mesh] OR "Diarrhea"[Mesh] OR "Lymphedema"[Mesh] OR toxicit*[tiab] OR adverse[tiab] OR "side effect"[tiab] OR "side effects"[tiab] OR mucositis[tiab] OR diarrhea[tiab] OR diarrhoea[tiab] OR myelosuppression[tiab] OR hematologic*[tiab] OR haematologic*[tiab] OR neutropenia[tiab] OR leukopenia[tiab] OR thrombocytopenia[tiab] OR nephrotox*[tiab] OR neuropath*[tiab] OR lymphedema[tiab] OR lymphoedema[tiab] OR dermatitis[tiab] OR

[3/3] radiodermatitis[tiab] OR xerostomia[tiab] OR dysphagia[tiab] OR erysipelas[tiab] OR infection*[tiab]) AND (clinical trial[pt] OR randomized controlled trial[pt] OR controlled clinical trial[pt] OR clinical trial[tiab] OR trial[tiab] OR randomized[tiab] OR randomised[tiab] OR placebo[tiab] OR double-blind[tiab] OR controlled[tiab]) NOT (animals[mh] NOT humans[mh])

**Web of Science**

**One-line query**

[1/2] TS=( (selenium OR selenite OR selenomethionine OR "sodium selenite" OR "selenium yeast" OR selenious) AND (cancer* OR tumor* OR tumour* OR neoplasm* OR carcinoma* OR lymphoma OR leukemia OR leukaemia OR malignan*) AND (chemotherap* OR radiotherap* OR radiation OR irradiat* OR chemoradiotherap* OR "radioactive iodine" OR radioiodine OR surgery OR surgical OR "endocrine therap*" OR "hormone therap*" OR platinum OR paclitaxel OR carboplatin OR cisplatin) AND (toxicit* OR adverse OR "side effect*" OR mucositis OR diarrhea OR diarrhoea OR myelosuppression OR hematologic* OR haematologic* OR neutropenia OR leukopenia OR thrombocytopenia OR

[2/2] nephrotox* OR neuropath* OR lymphedema OR lymphoedema OR dermatitis OR radiodermatitis OR xerostomia OR dysphagia OR erysipelas OR infection*) AND (trial OR randomized OR randomised OR placebo OR "double blind" OR controlled) )

**Structured search blocks**

[1/2] TS=( (selenium OR selenite OR selenomethionine OR "sodium selenite" OR "selenium yeast" OR selenious) AND (cancer* OR tumor* OR tumour* OR neoplasm* OR carcinoma* OR lymphoma OR leukemia OR leukaemia OR malignan*) AND (chemotherap* OR radiotherap* OR radiation OR irradiat* OR chemoradiotherap* OR "radioactive iodine" OR radioiodine OR surgery OR surgical OR "endocrine therap*" OR "hormone therap*" OR platinum OR paclitaxel OR carboplatin OR cisplatin) AND (toxicit* OR adverse OR "side effect*" OR mucositis OR diarrhea OR diarrhoea OR myelosuppression OR hematologic* OR haematologic* OR neutropenia OR leukopenia OR thrombocytopenia OR

[2/2] nephrotox* OR neuropath* OR lymphedema OR lymphoedema OR dermatitis OR radiodermatitis OR xerostomia OR dysphagia OR erysipelas OR infection*) AND (trial OR randomized OR randomised OR placebo OR "double blind" OR controlled) )

**Cochrane Library**

**One-line query**

[1/2] (selenium OR selenite OR selenomethionine OR "sodium selenite" OR "selenium yeast" OR selenious):ti,ab,kw AND (cancer* OR tumor* OR tumour* OR neoplasm* OR carcinoma* OR lymphoma OR leukemia OR leukaemia OR malignan*):ti,ab,kw AND (chemotherap* OR radiotherap* OR radiation OR irradiat* OR chemoradiotherap* OR "radioactive iodine" OR radioiodine OR surgery OR surgical OR "endocrine therap*" OR "hormone therap*" OR platinum OR paclitaxel OR carboplatin OR cisplatin):ti,ab,kw AND (toxicit* OR adverse OR "side effect*" OR mucositis OR diarrhea OR diarrhoea OR myelosuppression OR hematologic* OR haematologic* OR neutropenia OR leukopenia OR

[2/2] thrombocytopenia OR nephrotox* OR neuropath* OR lymphedema OR lymphoedema OR dermatitis OR radiodermatitis OR xerostomia OR dysphagia OR erysipelas OR infection*):ti,ab,kw

**Structured search blocks**

[1/2] (selenium OR selenite OR selenomethionine OR "sodium selenite" OR "selenium yeast" OR selenious):ti,ab,kw AND (cancer* OR tumor* OR tumour* OR neoplasm* OR carcinoma* OR lymphoma OR leukemia OR leukaemia OR malignan*):ti,ab,kw AND (chemotherap* OR radiotherap* OR radiation OR irradiat* OR chemoradiotherap* OR "radioactive iodine" OR radioiodine OR surgery OR surgical OR "endocrine therap*" OR "hormone therap*" OR platinum OR paclitaxel OR carboplatin OR cisplatin):ti,ab,kw AND (toxicit* OR adverse OR "side effect*" OR mucositis OR diarrhea OR diarrhoea OR myelosuppression OR hematologic* OR haematologic* OR neutropenia OR leukopenia OR

[2/2] thrombocytopenia OR nephrotox* OR neuropath* OR lymphedema OR lymphoedema OR dermatitis OR radiodermatitis OR xerostomia OR dysphagia OR erysipelas OR infection*):ti,ab,kw

**Cochrane Library**

**One-line query**

[1/2] (selenium OR selenite OR selenomethionine OR "sodium selenite" OR "selenium yeast" OR selenious):ti,ab,kw AND (cancer* OR tumor* OR tumour* OR neoplasm* OR carcinoma* OR lymphoma OR leukemia OR leukaemia OR malignan*):ti,ab,kw AND (chemotherap* OR radiotherap* OR radiation OR irradiat* OR chemoradiotherap* OR "radioactive iodine" OR radioiodine OR surgery OR surgical OR "endocrine therap*" OR "hormone therap*" OR platinum OR paclitaxel OR carboplatin OR cisplatin):ti,ab,kw AND (toxicit* OR adverse OR "side effect*" OR mucositis OR diarrhea OR diarrhoea OR myelosuppression OR hematologic* OR haematologic* OR neutropenia OR leukopenia OR

[2/2] thrombocytopenia OR nephrotox* OR neuropath* OR lymphedema OR lymphoedema OR dermatitis OR radiodermatitis OR xerostomia OR dysphagia OR erysipelas OR infection*):ti,ab,kw

**Structured search blocks**

[1/2] (selenium OR selenite OR selenomethionine OR "sodium selenite" OR "selenium yeast" OR selenious):ti,ab,kw AND (cancer* OR tumor* OR tumour* OR neoplasm* OR carcinoma* OR lymphoma OR leukemia OR leukaemia OR malignan*):ti,ab,kw AND (chemotherap* OR radiotherap* OR radiation OR irradiat* OR chemoradiotherap* OR "radioactive iodine" OR radioiodine OR surgery OR surgical OR "endocrine therap*" OR "hormone therap*" OR platinum OR paclitaxel OR carboplatin OR cisplatin):ti,ab,kw AND (toxicit* OR adverse OR "side effect*" OR mucositis OR diarrhea OR diarrhoea OR myelosuppression OR hematologic* OR haematologic* OR neutropenia OR leukopenia OR

[2/2] thrombocytopenia OR nephrotox* OR neuropath* OR lymphedema OR lymphoedema OR dermatitis OR radiodermatitis OR xerostomia OR dysphagia OR erysipelas OR infection*):ti,ab,kw

Note. Searches were run without date or language restrictions. Cochrane CENTRAL and Cochrane Reviews counts were recorded separately. Consecutive Word-display segments reconstruct each exact query in token order. Counts reconcile to the PRISMA flow diagram.

**Supplementary Table S2. Reports excluded after full-text assessment**

| **Citation** | **DOI or PMID** | **Full text assessed** | **Exclusion category** | **Specific exclusion reason** | **Decision date** | **Review status** | **Notes** |
| --- | --- | --- | --- | --- | --- | --- | --- |
| Lee Seoha (2021). Effects of Sodium Selenite Injection on Serum Metabolic Profiles in Women Diagnosed with Breast Cancer-Related Lymphedema-Secondary Analysis of a Randomized Placebo-Controlled Trial Using Global Metabolomics. Nutrients. | DOI: 10.3390/nu13093253; PMID: 34579131 | Yes | Duplicate cohort | Secondary metabolomics analysis of the same breast-cancer-related lymphedema trial represented by the corresponding primary trial report. | 2026-07-10 | Final consensus decision after independent full-text review | Not applicable |
| Muecke Ralph (2014). Multicenter, phase III trial comparing selenium supplementation with observation in gynecologic radiation oncology: follow-up analysis of the survival data 6 years after cessation of randomization. Integrative cancer therapies. | DOI: 10.1177/1534735414541963; PMID: 25015649 | Yes | Duplicate cohort | Survival follow-up companion to the gynecologic radiation oncology selenium trial represented by the corresponding primary trial report. | 2026-07-10 | Final consensus decision after independent full-text review | Not applicable |
| Muecke Ralph (2013). Impact of treatment planning target volumen (PTV) size on radiation induced diarrhoea following selenium supplementation in gynecologic radiation oncology--a subgroup analysis of a multicenter, phase III trial. Radiation oncology (London, England). | DOI: 10.1186/1748-717X-8-72; PMID: 23531280 | Yes | Duplicate cohort | Treatment-planning/PTV secondary analysis linked to the gynecologic RT selenium trial represented by the corresponding primary trial report. | 2026-07-10 | Final consensus decision after independent full-text review | Not applicable |
| Büntzel Jens (2010). Selenium substitution during radiotherapy of solid tumours - laboratory data from two observation studies in gynaecological and head and neck cancer patients. Anticancer research. | PMID: 20592379 | Yes | Duplicate cohort | Laboratory/context report from two radiotherapy studies likely overlapping SG07 and SG10. | 2026-07-10 | Final consensus decision after independent full-text review | Not applicable |
| Büntzel J (1999). [Experiences with sodium selenite in treatment of acute and late adverse effects of radiochemotherapy of head-neck carcinomas. Cytoprotection Working Group in AK Supportive Measures in Oncology Within the scope of MASCC and DKG]. Medizinische Klinik (Munich, Germany : 1983). | DOI: 10.1007/BF03042192; PMID: 10554530 | Yes | Wrong exposure or intervention | The controlled phase-II comparison in this report evaluated amifostine, whereas the selenium data were uncontrolled treatment observations for established extravasation or lymphatic edema. This does not test selenium prevention during cancer therapy. | 2026-07-10 | Final consensus decision after independent full-text review | Not applicable |
| Hehr T (1997). [Role of sodium selenite as an adjuvant in radiotherapy of rectal carcinoma]. Medizinische Klinik (Munich, Germany : 1983). | DOI: 10.1007/BF03041966; PMID: 9417501 | Yes | Wrong comparator | Preliminary single-group selenium experience during rectal radiochemotherapy did not provide an eligible concurrent selenium-free comparator or extractable comparative toxicity effect; the article states that statistically valid efficacy conclusions were not possible. | 2026-07-10 | Final consensus decision after independent full-text review | Not applicable |
| Daeian, Nesa (2014). Selenium supplementation in patients undergoing hematopoietic stem cell transplantation: effects on pro-inflammatory cytokines levels DARU-JOURNAL OF PHARMACEUTICAL SCIENCES. | DOI: 10.1186/2008-2231-22-51; PMID: 24942646 | Yes | Duplicate cohort | Cytokine/secondary companion report linked to the HSCT mucositis RCT represented by the corresponding primary trial report. | 2026-07-10 | Final consensus decision after independent full-text review | Not applicable |
| Breuer, Olof (2023). Intravenous Infusion of High Dose Selenite in End-Stage Cancer Patients: Analysis of Systemic Exposure to Selenite and Seleno-Metabolites BIOMEDICINES. | DOI: 10.3390/biomedicines11020295; PMID: 36830832 | Yes | Duplicate cohort | Metabolite/systemic exposure companion to phase I high-dose selenite safety evidence represented by the corresponding primary trial report. | 2026-07-10 | Final consensus decision after independent full-text review | Not applicable |
| Muecke R (2012). Long-term survival following supplementation of sodium selenite during adjuvant radiotherapy (RT) in patients with gynecological cancer Supportive care in cancer. | DOI: 10.1007/s00520-012-1479-7 | Yes | Duplicate cohort | Conference/supplement abstract reporting survival follow-up of the gynecologic RT selenium cohort. | 2026-07-10 | Final consensus decision after independent full-text review | Not applicable |
| Alhazmi W (2025). Efficacy and safety of intravenous administration of high-dose selenium for preventing chemotherapy-induced peripheral neuropathy in gastric cancer patients receiving adjuvant oxaliplatin and capecitabine after gastrectomy: a retrospective pilot study Korean Journal of Clinical Oncology. | DOI: 10.14216/kjco.25349 | Yes | Wrong study design | Retrospective pilot study; the observational design did not meet the prespecified eligibility criteria. | 2026-07-10 | Final consensus decision after independent full-text review | Not applicable |

*Note. Ten retrieved reports were excluded after full-text assessment. Eight additional sought reports were not retrieved and are shown as retrieval failures in the PRISMA flow, not as scientific exclusions.*

**Supplementary Table S3. Participant and baseline characteristics**

**Panel A. Demographic and clinical baseline characteristics**

| **Study** | **Group or exposure level** | **N** | **Age** | **Sex** | **Disease stage or severity** | **Key comorbidity** |
| --- | --- | --- | --- | --- | --- | --- |
| Yim 2026 | Selenium | 34 randomized; 30 PP | PP Table 2: >=60 years 18/30 selenium vs 15/30 placebo | Female cancer population | Initial FIGO stage 3-4 majority; Table 2 | ECOG 0-2; normal hematologic, renal, hepatic organ function required |
| Yim 2026 | Normal saline placebo | 34 randomized; 30 PP | PP Table 2: >=60 years 18/30 selenium vs 15/30 placebo | Female cancer population | Initial FIGO stage 3-4 majority; Table 2 | ECOG 0-2; normal hematologic, renal, hepatic organ function required |
| Yang 2023 | Experimental group | 50 | Baseline characteristics were reported as similar | Female cervical cancer population | FIGO IIB | Treatment-naive, PS 0-2, adequate organ function |
| Yang 2023 | Control group | 54 | Baseline characteristics were reported as similar | Female cervical cancer population | FIGO IIB | Treatment-naive, PS 0-2, adequate organ function |
| Laali 2020 | Selenium group | 37 | Mean age 52.14 selenium vs 54.74 placebo; median 51 vs 57.5 | Male 25/37 selenium vs 25/34 placebo | Not reported; RT dose <=6000/>6000 Gy categories reported | Karnofsky >70; normal renal function required |
| Laali 2020 | Placebo group | 34 | Mean age 52.14 selenium vs 54.74 placebo; median 51 vs 57.5 | Male 25/37 selenium vs 25/34 placebo | Not reported; RT dose <=6000/>6000 Gy categories reported | Karnofsky >70; normal renal function required |
| Mix 2015 | SLM | 10 | Median age 59.5 SLM vs 55.5 placebo | Male 10/10 SLM vs 7/8 placebo | All stage IVA or IVB except one unknown in SLM; Table 1 | Stage III/IV; one SLM patient took one dose and withdrew |
| Mix 2015 | Placebo | 8 | Median age 59.5 SLM vs 55.5 placebo | Male 10/10 SLM vs 7/8 placebo | All stage IVA or IVB except one unknown in SLM; Table 1 | Stage III/IV; one SLM patient took one dose and withdrew |
| Vieira 2015 | Crossover selenium period | Not parallel | Average age 8.2 +/- 4.4 leukemia/lymphoma; 7.4 +/- 4.3 solid tumors | Mixed male/female population; exact totals not reported | Not applicable/mixed pediatric diagnoses | On chemotherapy; crossover supportive-care study |
| Vieira 2015 | Crossover placebo period | Not parallel | Average age 8.2 +/- 4.4 leukemia/lymphoma; 7.4 +/- 4.3 solid tumors | Mixed male/female population; exact totals not reported | Not applicable/mixed pediatric diagnoses | On chemotherapy; crossover supportive-care study |
| Jahangard-Rafsanjani 2013 | Selenium group | 37 | Mean age 33.3 selenium vs 34.0 control; median 32 vs 32 | Male 21/37 in both groups | CR1/CR2/CR3 distributions reported in Table 1 | Adequate cardiac, pulmonary, renal, hepatic function; KPS >=70 |
| Jahangard-Rafsanjani 2013 | Control group | 37 | Mean age 33.3 selenium vs 34.0 control; median 32 vs 32 | Male 21/37 in both groups | CR1/CR2/CR3 distributions reported in Table 1 | Adequate cardiac, pulmonary, renal, hepatic function; KPS >=70 |
| Ghorbani 2013 | Selenium | 61 | 44.77 years selenium vs 46.37 years placebo | Female 18/61 selenium vs 19/61 placebo | Not reported | Baseline creatinine 0.80 mg/dL selenium vs 0.83 mg/dL placebo |
| Ghorbani 2013 | Placebo | 61 | 44.77 years selenium vs 46.37 years placebo | Female 18/61 selenium vs 19/61 placebo | Not reported | Baseline creatinine 0.80 mg/dL selenium vs 0.83 mg/dL placebo |
| Muecke 2010 | With selenium | 39 | Median age 64.8 selenium vs 63.8 control | Female gynecologic cancer population | FIGO I-IV reported in Table 1 | Whole-blood selenium <84 µg/L required |
| Muecke 2010 | Without selenium | 42 | Median age 64.8 selenium vs 63.8 control | Female gynecologic cancer population | FIGO I-IV reported in Table 1 | Whole-blood selenium <84 µg/L required |
| Buentzel 2010 | Selenium group | 22 | Median 62.8 selenium vs 63.5 control | Not reported | Table I reports localization/stage but not fully reported in the available article | Selenium deficiency and RT field including 75% of major salivary glands |
| Buentzel 2010 | Control group | 17 | Median 62.8 selenium vs 63.5 control | Not reported | Table I reports localization/stage but not fully reported in the available article | Selenium deficiency and RT field including 75% of major salivary glands |
| Asfour 2006 | Chemotherapy plus sodium selenite | 15 | Not reported | 14 males and 16 females overall | Intermediate/high-grade NHL | Newly diagnosed NHL |
| Asfour 2006 | Conventional chemotherapy | 15 | Not reported | 14 males and 16 females overall | Intermediate/high-grade NHL | Newly diagnosed NHL |
| Hu 1997 | Seleno-Kappacarrageenan cycle | 41 cycles | Age 31-72 | Male:female 27:14 | KPS >60; stage not reported | At least two cycles of same cisplatin-containing regimen |
| Hu 1997 | No selenium cycle | 41 cycles | Age 31-72 | Male:female 27:14 | KPS >60; stage not reported | At least two cycles of same cisplatin-containing regimen |
| Ameri 2016 | Oral selenium | 15 | Age values remain translation-sensitive | Mixed male and female population; exact totals remain translation-sensitive | Not clearly reported | At least two oral sites examined |
| Ameri 2016 | Placebo | 15 | Age values remain translation-sensitive | Mixed male and female population; exact totals remain translation-sensitive | Not clearly reported | At least two oral sites examined |
| Chen 2023 | Observation group | 43 | Observation 60.89 +/- 11.72 years; control 61.28 +/- 9.34 years | Observation 26 male/17 female; control 23 male/20 female | Not reported | No prior surgery, radiotherapy, or chemotherapy |
| Chen 2023 | Control group | 43 | Observation 60.89 +/- 11.72 years; control 61.28 +/- 9.34 years | Observation 26 male/17 female; control 23 male/20 female | Not reported | No prior surgery, radiotherapy, or chemotherapy |
| Wang 2020 | Selenium yeast group | 30 | Selenium 55.3 +/- 10.4 years; routine 52.3 +/- 10.5 years | Selenium 16 male/14 female; routine 17 male/13 female | Selenium: stage II 8, III 16, IVA 6; routine: stage II 8, III 15, IVA 7 | Intact oral mucosa; no baseline xerostomia, oral pain, or oral ulceration |
| Wang 2020 | Routine treatment group | 30 | Selenium 55.3 +/- 10.4 years; routine 52.3 +/- 10.5 years | Selenium 16 male/14 female; routine 17 male/13 female | Selenium: stage II 8, III 16, IVA 6; routine: stage II 8, III 15, IVA 7 | Intact oral mucosa; no baseline xerostomia, oral pain, or oral ulceration |
| Son 2017 | Selenium supplement group | 8 | Median (range): selenium 40 (20-67), control 50 (36-65) years | Selenium 3 male/5 female; control 3 male/5 female | Selenium T2N1 1, T3N0 1, T3N1 6; control T1N1 1, T3N1 7 | No prior I-131 treatment or neck radiotherapy; baseline salivary parameters comparable |
| Son 2017 | Placebo control group | 8 | Median (range): selenium 40 (20-67), control 50 (36-65) years | Selenium 3 male/5 female; control 3 male/5 female | Selenium T2N1 1, T3N0 1, T3N1 6; control T1N1 1, T3N1 7 | No prior I-131 treatment or neck radiotherapy; baseline salivary parameters comparable |
| Bahador 2022 | Se-group | 40 | Mean +/- SD: Se 59.5 +/- 12.8; control 63.8 +/- 11.2 years | Se 20 male/20 female; control 28 male/12 female | Not reported | Baseline GI and urinary diseases excluded |
| Bahador 2022 | Control/placebo group | 40 | Mean +/- SD: Se 59.5 +/- 12.8; control 63.8 +/- 11.2 years | Se 20 male/20 female; control 28 male/12 female | Not reported | Baseline GI and urinary diseases excluded |
| Pavlidis 2026 | Se group | 17 | Control 57.93 +/- 3.7; Se 55.56 +/- 3.42 years; Zn group also present | Control 14 male/3 female; Se paragraph reports 13 male/3 female despite 17 per group in abstract | Matched for illness stage I/II vs III/IV; detailed distribution not extracted | Severe OM or oral bacterial infections before therapy excluded |
| Pavlidis 2026 | Control group | 17 | Control 57.93 +/- 3.7; Se 55.56 +/- 3.42 years; Zn group also present | Control 14 male/3 female; Se paragraph reports 13 male/3 female despite 17 per group in abstract | Matched for illness stage I/II vs III/IV; detailed distribution not extracted | Severe OM or oral bacterial infections before therapy excluded |

**Panel B. Baseline outcome, treatment, follow-up, and reporting completeness**

| **Study** | **Group or exposure level** | **Baseline outcome value** | **Treatment background** | **Follow-up** | **Missing baseline data** |
| --- | --- | --- | --- | --- | --- |
| Yim 2026 | Selenium | Not reported | Prior treatment and surgery balanced in Table 2 | Total follow-up 29.8 +/- 13.3 months selenium vs 31.5 +/- 11.3 months placebo | PP and ITT denominators differ by outcome. |
| Yim 2026 | Normal saline placebo | Not reported | Prior treatment and surgery balanced in Table 2 | Total follow-up 29.8 +/- 13.3 months selenium vs 31.5 +/- 11.3 months placebo | PP and ITT denominators differ by outcome. |
| Yang 2023 | Experimental group | Baseline whole blood Se 58.34 +/- 17.63 ug/L selenium vs 60.21 +/- 18.41 ug/L control | All received pelvic RT plus weekly cisplatin and brachytherapy | During concurrent chemoradiotherapy; longer survival follow-up planned | No additional missing baseline item recorded. |
| Yang 2023 | Control group | Baseline whole blood Se 58.34 +/- 17.63 ug/L selenium vs 60.21 +/- 18.41 ug/L control | All received pelvic RT plus weekly cisplatin and brachytherapy | During concurrent chemoradiotherapy; longer survival follow-up planned | No additional missing baseline item recorded. |
| Laali 2020 | Selenium group | Before radiation 79.49 +/- 25.63 mcg/L selenium vs 85.38 +/- 30.63 placebo | Concurrent chemotherapy 18/37 selenium vs 16/34 placebo | Weekly during radiotherapy and one month after radiotherapy | No additional missing baseline item recorded. |
| Laali 2020 | Placebo group | Before radiation 79.49 +/- 25.63 mcg/L selenium vs 85.38 +/- 30.63 placebo | Concurrent chemotherapy 18/37 selenium vs 16/34 placebo | Weekly during radiotherapy and one month after radiotherapy | No additional missing baseline item recorded. |
| Mix 2015 | SLM | NZ subset baseline mean plasma Se 80.2 ng/mL SLM vs 105.1 ng/mL placebo | RT 70 Gy plus cisplatin; chemotherapy cycles varied | During CRT and 1-year follow-up for QOL/survival; trial stopped early | Underpowered stopped trial. |
| Mix 2015 | Placebo | NZ subset baseline mean plasma Se 80.2 ng/mL SLM vs 105.1 ng/mL placebo | RT 70 Gy plus cisplatin; chemotherapy cycles varied | During CRT and 1-year follow-up for QOL/survival; trial stopped early | Underpowered stopped trial. |
| Vieira 2015 | Crossover selenium period | Not reported | Mixed chemotherapy protocols | 30-day selenium/placebo periods plus 1-year supplementation follow-up | Not pooled as a parallel-group RCT because crossover-specific paired estimates were unavailable. |
| Vieira 2015 | Crossover placebo period | Not reported | Mixed chemotherapy protocols | 30-day selenium/placebo periods plus 1-year supplementation follow-up | Not pooled as a parallel-group RCT because crossover-specific paired estimates were unavailable. |
| Jahangard-Rafsanjani 2013 | Selenium group | Serum Se 6.47 +/- 1.53 mcg/dL selenium vs 7.13 +/- 1.70 control | Same conditioning and mucositis prophylaxis regimen | Daily OM assessment to day 21 post-transplant or resolution; 3-month mortality | No additional missing baseline item recorded. |
| Jahangard-Rafsanjani 2013 | Control group | Serum Se 6.47 +/- 1.53 mcg/dL selenium vs 7.13 +/- 1.70 control | Same conditioning and mucositis prophylaxis regimen | Daily OM assessment to day 21 post-transplant or resolution; 3-month mortality | No additional missing baseline item recorded. |
| Ghorbani 2013 | Selenium | Not reported | Cisplatin cumulative dose distribution similar; p=0.54 | Creatinine/BUN repeated on day 5 after cisplatin | No additional missing baseline item recorded. |
| Ghorbani 2013 | Placebo | Not reported | Cisplatin cumulative dose distribution similar; p=0.54 | Creatinine/BUN repeated on day 5 after cisplatin | No additional missing baseline item recorded. |
| Muecke 2010 | With selenium | Before RT 65.3 µg/L selenium vs 63.2 µg/L control | Brachytherapy 35/39 vs 39/42; external RT dose similar | During RT; 6 weeks after RT; median 49 months DFS and 51 months OS | Observation/no-supplement control. |
| Muecke 2010 | Without selenium | Before RT 65.3 µg/L selenium vs 63.2 µg/L control | Brachytherapy 35/39 vs 39/42; external RT dose similar | During RT; 6 weeks after RT; median 49 months DFS and 51 months OS | Observation/no-supplement control. |
| Buentzel 2010 | Selenium group | Deficiency required; detailed selenium values in separate paper | Definitive/adjuvant RT; adjuvant 20/22 vs 17/17 | Weekly during RT and 6 weeks after RT | No additional missing baseline item recorded. |
| Buentzel 2010 | Control group | Deficiency required; detailed selenium values in separate paper | Definitive/adjuvant RT; adjuvant 20/22 vs 17/17 | Weekly during RT and 6 weeks after RT | No additional missing baseline item recorded. |
| Asfour 2006 | Chemotherapy plus sodium selenite | Not reported | All received CHOP; selenium group received sodium selenite on days 3-7 | Early post-chemotherapy apoptosis/infection/cardiac function assessment | Small mechanistic trial. |
| Asfour 2006 | Conventional chemotherapy | Not reported | All received CHOP; selenium group received sodium selenite on days 3-7 | Early post-chemotherapy apoptosis/infection/cardiac function assessment | Small mechanistic trial. |
| Hu 1997 | Seleno-Kappacarrageenan cycle | Serum Se increased from 70.40 to 157.04 ng/mL after Se | Crossover group A/B with Se in first or second cycle | Urine enzymes through 72 hours and blood counts through day 20 after chemotherapy | Not a simple parallel-group comparison. |
| Hu 1997 | No selenium cycle | Serum Se increased from 70.40 to 157.04 ng/mL after Se | Crossover group A/B with Se in first or second cycle | Urine enzymes through 72 hours and blood counts through day 20 after chemotherapy | Not a simple parallel-group comparison. |
| Ameri 2016 | Oral selenium | Not reported | Radiotherapy; details not clearly reported | Weekly during RT; intervention through 3 weeks after RT | Exact analyzed denominators were verified as 15 per arm; untranslated baseline details are not used in effect calculations. |
| Ameri 2016 | Placebo | Not reported | Radiotherapy; details not clearly reported | Weekly during RT; intervention through 3 weeks after RT | Exact analyzed denominators were verified as 15 per arm; untranslated baseline details are not used in effect calculations. |
| Chen 2023 | Observation group | Not reported | Cisplatin 19/18, carboplatin 11/11, oxaliplatin 8/11, nedaplatin 5/3 for observation/control | 1 month | The mixed malignancy distribution limits disease-specific subgroup interpretation. |
| Chen 2023 | Control group | Not reported | Cisplatin 19/18, carboplatin 11/11, oxaliplatin 8/11, nedaplatin 5/3 for observation/control | 1 month | The mixed malignancy distribution limits disease-specific subgroup interpretation. |
| Wang 2020 | Selenium yeast group | Not reported | Both groups received IMRT plus concurrent cisplatin and Kangfuxin solution | Through end of concurrent chemoradiotherapy and short-term response assessment | Chinese-language variables translated literally. |
| Wang 2020 | Routine treatment group | Not reported | Both groups received IMRT plus concurrent cisplatin and Kangfuxin solution | Through end of concurrent chemoradiotherapy and short-term response assessment | Chinese-language variables translated literally. |
| Son 2017 | Selenium supplement group | Median whole blood selenium 10.3 vs 10.2 ug/dL | I-131 dose 3.7 GBq in 4/8 and 5.6 GBq in 4/8 in each arm | 6 months | All continuous values are medians with ranges, not means/SDs. |
| Son 2017 | Placebo control group | Median whole blood selenium 10.3 vs 10.2 ug/dL | I-131 dose 3.7 GBq in 4/8 and 5.6 GBq in 4/8 in each arm | 6 months | All continuous values are medians with ranges, not means/SDs. |
| Bahador 2022 | Se-group | Not reported | Four-field RT 50-70 Gy; capecitabine for rectal and cisplatin for cervix/bladder cancers | During treatment and weeks after radiotherapy | Article text has limited disease-regimen stratification; raw toxicity counts unavailable. |
| Bahador 2022 | Control/placebo group | Not reported | Four-field RT 50-70 Gy; capecitabine for rectal and cisplatin for cervix/bladder cancers | During treatment and weeks after radiotherapy | Article text has limited disease-regimen stratification; raw toxicity counts unavailable. |
| Pavlidis 2026 | Se group | Not reported | Three arms: no supplement control, zinc, selenium; analysis here records control vs selenium context only | Through 1 month after second chemotherapy cycle | Group counts and concurrent chemotherapy details were not consistently reported. |
| Pavlidis 2026 | Control group | Not reported | Three arms: no supplement control, zinc, selenium; analysis here records control vs selenium context only | Through 1 month after second chemotherapy cycle | Group counts and concurrent chemotherapy details were not consistently reported. |

*Note. Group-specific values are shown when available; study-level summaries are repeated across arms where the source did not provide separable arm values. PP, per protocol; ITT, intention to treat.*

**Supplementary Table S4. Outcome definitions, cutoffs, time points, and harmonization**

**Panel A. Original and harmonized outcome definitions**

| **Study** | **Outcome domain** | **Original outcome definition** | **Harmonized outcome label** | **Cutoff or threshold** | **Time point** |
| --- | --- | --- | --- | --- | --- |
| Yim 2026 | CIPN | Proportion of patients exhibiting grade 1 or more neuropathy manifested as paresthesia, pain, or motor dysfunction | Grade 1 or more CIPN | Grade >=1 | 3 months after six cycles of chemotherapy |
| Yim 2026 | CIPN | Motor dysfunction requiring intervention/medication or limiting ADL | Grade 2 or more motor dysfunction | Grade >=2 | Before chemotherapy cycles 3 and 4 |
| Yim 2026 | CIPN | Patient-reported sensory neuropathy score from the EORTC CIPN20 instrument | CIPN20 sensory score | Continuous score | 3 months after chemotherapy |
| Yang 2023 | Hematologic toxicity | Concurrent chemoradiotherapy-related myelosuppression by grade | Myelosuppression | Grade 3 and grade 4 separately | During concurrent chemoradiotherapy |
| Laali 2020 | Oral mucositis | Oral mucositis grade 0-4 with severe OM defined as grades 3-4 | Incidence and severity of OM | Any grade 1-4; severe grade 3-4 | Weekly during RT and one month after RT |
| Laali 2020 | Oral mucositis | Time from radiotherapy initiation to oral mucositis onset | Oral mucositis onset time | Continuous weeks; higher is later onset/favorable | During radiotherapy |
| Mix 2015 | Oral mucositis | Mucositis grades 0-3 in Table 2; grade 3 considered severe; no grade 4 mucosal toxicity reported | Mucositis grade distribution | Grade 3-4 | During 7 weeks concurrent CRT |
| Vieira 2015 | Supportive symptoms/QoL | EORTC QLQ-C30-derived symptom/function scores reported as medians and quartiles | Fatigue, nausea, appetite loss, physical function | Continuous score; higher symptom score worse and higher physical function score better | Initial, placebo period, selenium period, and 1-year follow-up |
| Jahangard-Rafsanjani 2013 | Oral mucositis | WHO OM grade 0-4; severe OM grades 3-4; objective OM grades 2-4 duration | OM incidence, severe OM, duration | Any grade 1-4; severe grade 3-4; grade 2-4 duration | Starting day of HDC to 21 days after transplantation or OM resolution |
| Ghorbani 2013 | Nephrotoxicity | Plasma creatinine above 1.5 mg/dL in men or 1.4 mg/dL in women, or >50% increase from baseline, or urine flow <0.5 mL/kg/h after cisplatin infusion | Acute kidney injury after cisplatin | AKI event yes/no | Day 5 after cisplatin therapy |
| Muecke 2010 | Radiation-induced diarrhea | Radiation-induced diarrhea by Common Toxicity Criteria | CTC diarrhea during pelvic RT | Grade 1-3 and grade >=2 | Week 4 of RT and actuarial time to grade >=2 diarrhea |
| Buentzel 2010 | Head-and-neck radiation toxicity | Maximal dysphagia, loss of taste, dry mouth, and stomatitis grades 0-4 | Maximal RTOG toxicities | Grade >=3 and grade >=2 derivable from grade distribution | During RT and 6 weeks after RT; maximal toxicity |
| Asfour 2006 | Infection/cardiac/immune toxicity | Post-chemotherapy infection rate; peripheral neutrophil apoptosis by flow cytometry; cardiac ejection fraction | Infection rate, neutrophil apoptosis, cardiac ejection fraction | Infection yes/no; continuous apoptosis and EF | Day 8 after chemotherapy and early post-cycle period |
| Hu 1997 | Cisplatin toxicity | WBC counts, GCSF use, blood transfusion, and urine enzymes NAG/GGT/AAP/LAP/ALP after cisplatin | Bone marrow suppression and renal tubular enzymes | Continuous laboratory values | Day 14 WBC; 2, 24, 48, 72 hours urine enzymes after chemotherapy |
| Ameri 2016 | Oral mucositis | Oral cavity divided into 14 anatomical sites; each site scored using 4-point scale; mean mucositis score calculated | Mean mucositis score | Continuous mucositis score | Weeks 1, 2, and 3 during radiotherapy |
| Chen 2023 | Broad adverse reactions | Any treatment-related adverse reaction after platinum-containing combined regimen, grades I-IV versus grade 0 | Any adverse reaction grade I-IV | Any grade I-IV | After 1 month/treatment course |
| Chen 2023 | Broad adverse reactions | Grade III plus grade IV adverse reactions from same grade table | Grade III-IV adverse reactions | Grade III-IV | After 1 month/treatment course |
| Chen 2023 | Gastrointestinal toxicity | Gastrointestinal reactions summed across platinum drug subgroups | Gastrointestinal reactions | Any grade | After 1 month/treatment course |
| Chen 2023 | Hematologic toxicity | Bone marrow suppression summed across platinum drug subgroups | Bone marrow suppression | Any grade | After 1 month/treatment course |
| Chen 2023 | Pain | Moderate or severe pain from descriptive pain grade table | Pain grade >=2 | Grade >=2 | During treatment |
| Wang 2020 | Oral mucositis | RTOG acute radiation injury oral mucositis grade 3 or 4 | Severe oral mucositis | Grade >=3 | During concurrent chemoradiotherapy |
| Wang 2020 | Oral mucositis | Days to oral mucositis onset | Oral mucositis onset time | Continuous days; higher is later onset/favorable | During concurrent chemoradiotherapy |
| Wang 2020 | Pain | Moderate or severe pain caused by oral mucosal injury | Pain grade >=2 | Grade >=2 | During concurrent chemoradiotherapy |
| Wang 2020 | Pain | Days from maximum pain degree to reduction by one grade | Pain relief time | Continuous days; lower is favorable | During concurrent chemoradiotherapy |
| Son 2017 | Salivary gland injury | Serum amylase level 2 days after radioiodine therapy | Serum amylase 2 days after I-131 | Continuous; higher indicates salivary injury | 2 days after I-131 |
| Son 2017 | Salivary gland injury | Symptom questionnaire total score for sialadenitis symptoms | Questionnaire score 6 months after I-131 | Continuous; higher is worse | 6 months after I-131 |
| Son 2017 | Salivary gland injury | MUR, MSP, and EF of parotid and submandibular glands at baseline and 6 months | Salivary gland scintigraphy parameters | Continuous; direction depends on parameter | 6 months after I-131 |
| Bahador 2022 | Gastrointestinal toxicity | GEE ordinal comparisons for chemo/radiation-induced diarrhea grade categories | Diarrhea severity odds | Grade II/III vs lower grades | During and after chemoradiotherapy |
| Bahador 2022 | Other toxicity | GEE odds ratios for other complications | Pain and abdominal cramp odds | Not clearly specified | During and after chemoradiotherapy |
| Pavlidis 2026 | Oral mucosa/taste function | EGM threshold values at six oral-cavity locations modeled with linear mixed effects | Electrogustometry thresholds | Continuous dB; lower threshold is favorable | Baseline, end of cycle 1, end of cycle 2, 2 weeks post-cycle 2, 1 month post-cycle 2 |
| Pavlidis 2026 | Oral mucosa/taste function | Fungiform papillae morphology and tongue-apex vessel morphology types correlated with EGM thresholds | fPap and blood-vessel morphology | Ordinal morphology damage | Serial study time points |

**Panel B. Measurement, direction, compatibility, and reporting considerations**

| **Study** | **Harmonized outcome label** | **Measurement instrument** | **Unit** | **Direction of beneficial effect** | **Compatibility decision** |
| --- | --- | --- | --- | --- | --- |
| Yim 2026 | Grade 1 or more CIPN | Combined WHO-CIPN and NCI-CTCAE version 5.0 | Events/participants | For harmful toxicity outcomes, lower event risk favors selenium after aligning to selenium vs control. | M2 CIPN |
| Yim 2026 | Grade 2 or more motor dysfunction | Combined WHO-CIPN and NCI-CTCAE version 5.0 | Arm-level events/totals | For harmful toxicity outcomes, lower event risk favors selenium after aligning to selenium vs control. | M2 CIPN |
| Yim 2026 | CIPN20 sensory score | EORTC QLQ-CIPN20 | Arm-level mean/SD; single-study MD unless a compatible trial becomes available | For harmful symptom scores, a lower mean after alignment favors selenium. | M2 CIPN single-study display |
| Yang 2023 | Myelosuppression | NCI Common Toxicity Criteria version 3.0 | Arm-level events/totals | For harmful toxicity outcomes, lower event risk favors selenium after aligning to selenium vs control. | M2 hematologic/composite toxicity |
| Laali 2020 | Incidence and severity of OM | WHO oral toxicity scale | Arm-level events/totals and mean duration | For harmful toxicity outcomes, lower event risk favors selenium after aligning to selenium vs control.; For continuous toxicity scores/biomarkers, direction depends on outcome definition; preserve original before any MD/SMD. | M2 oral mucositis; M3 head-and-neck RT |
| Laali 2020 | Oral mucositis onset time | WHO oral toxicity scale | MD after transparent weeks-to-days conversion of both means and SDs | For oral-mucositis onset, a positive MD after alignment indicates later onset and favors selenium. | M2 oral mucositis onset; compatible with Wang 2020 after unit conversion |
| Mix 2015 | Mucositis grade distribution | Not explicitly named in extracted table; protocol targeted grade 3-4 mucositis | Arm-level events/totals | For harmful toxicity outcomes, lower event risk favors selenium after aligning to selenium vs control. | M2 oral mucositis; M3 head-and-neck CCRT |
| Vieira 2015 | Fatigue, nausea, appetite loss, physical function | EORTC QLQ-C30 questionnaire | Crossover medians/quartiles; narrative unless special extraction plan | For continuous toxicity scores/biomarkers, direction depends on outcome definition; preserve original before any MD/SMD. | Narrative |
| Jahangard-Rafsanjani 2013 | OM incidence, severe OM, duration | WHO oral toxicity scale | Arm-level events/totals and mean/SD days | For harmful toxicity outcomes, lower event risk favors selenium after aligning to selenium vs control.; For continuous toxicity scores/biomarkers, direction depends on outcome definition; preserve original before any MD/SMD. | M2 oral mucositis; M3 HDC/HSCT |
| Ghorbani 2013 | Acute kidney injury after cisplatin | Trial-defined acute kidney injury criteria | Arm-level events/totals | For harmful toxicity outcomes, lower event risk favors selenium after aligning to selenium vs control. | M2 nephrotoxicity |
| Muecke 2010 | CTC diarrhea during pelvic RT | Common Toxicity Criteria | Arm-level events/totals for week 4; reported actuarial incidence for grade >=2 | For harmful toxicity outcomes, lower event risk favors selenium after aligning to selenium vs control. | M2 diarrhea; M3 gynecologic RT |
| Buentzel 2010 | Maximal RTOG toxicities | RTOG scale | Arm-level grade distribution | For harmful toxicity outcomes, lower event risk favors selenium after aligning to selenium vs control. | M2 oral mucositis/radiation toxicity |
| Asfour 2006 | Infection rate, neutrophil apoptosis, cardiac ejection fraction | Trial-defined; no CTCAE grade extracted | Arm-level events/totals for infection; mean/SD for mechanistic outcomes | For harmful toxicity outcomes, lower event risk favors selenium after aligning to selenium vs control.; For continuous toxicity scores/biomarkers, direction depends on outcome definition; preserve original before any MD/SMD. | Narrative |
| Hu 1997 | Bone marrow suppression and renal tubular enzymes | Laboratory continuous markers; no CTCAE grade | Mean/SD values in crossover cycles | For continuous toxicity scores/biomarkers, direction depends on outcome definition; preserve original before any MD/SMD. | Narrative/sensitivity due crossover |
| Ameri 2016 | Mean mucositis score | NCI-CTC version 2 and Epstein scoring system | Mean/SD with verified n=15 per arm | For continuous toxicity scores/biomarkers, direction depends on outcome definition; preserve original before any MD/SMD. | M2 oral mucositis; single-study continuous display |
| Chen 2023 | Any adverse reaction grade I-IV | Article grade table; exact grading system not named | Events/participants | For harmful toxicity events, lower RR/event proportion favors selenium when group 1 is selenium. | Broad toxicity and platinum-toxicity pool |
| Chen 2023 | Grade III-IV adverse reactions | Article grade table; exact grading system not named | Events/participants | For harmful toxicity events, lower RR/event proportion favors selenium when group 1 is selenium. | Severe broad toxicity pool |
| Chen 2023 | Gastrointestinal reactions | Not reported | Events/participants | For harmful toxicity events, lower RR/event proportion favors selenium when group 1 is selenium. | GI toxicity exploratory pool |
| Chen 2023 | Bone marrow suppression | Not reported | Events/participants | For harmful toxicity events, lower RR/event proportion favors selenium when group 1 is selenium. | Hematologic toxicity exploratory pool |
| Chen 2023 | Pain grade >=2 | Article descriptive pain grading | Events/participants | For harmful toxicity events, lower RR/event proportion favors selenium when group 1 is selenium. | Pain exploratory display only |
| Wang 2020 | Severe oral mucositis | RTOG acute radiation injury criteria | Events/participants | For harmful toxicity events, lower RR/event proportion favors selenium when group 1 is selenium. | Main severe OM pool; HNC/NPC CCRT subgroup |
| Wang 2020 | Oral mucositis onset time | RTOG acute radiation injury criteria | MD/continuous mean SD | Higher mean indicates later onset and favors selenium; do not interpret as harmful-event direction. | OM onset narrative or continuous pool if compatible |
| Wang 2020 | Pain grade >=2 | Descriptive pain grading | Events/participants | For harmful toxicity events, lower RR/event proportion favors selenium when group 1 is selenium. | OM-related pain exploratory pool |
| Wang 2020 | Pain relief time | Descriptive pain grading | MD/continuous mean SD | Lower mean indicates shorter pain relief time and favors selenium. | Pain duration/relief narrative or continuous pool if compatible |
| Son 2017 | Serum amylase 2 days after I-131 | Laboratory biomarker | Median/range; not directly poolable with mean SD | Narrative/model/correlation result only; no harmonized pooled effect. | Narrative/single-study display |
| Son 2017 | Questionnaire score 6 months after I-131 | 14-item questionnaire, score 14-56 | Median/range; not directly poolable with mean SD | Narrative/model/correlation result only; no harmonized pooled effect. | Narrative/single-study display |
| Son 2017 | Salivary gland scintigraphy parameters | Salivary gland scintigraphy | Median/range; within-arm P values | Narrative/model/correlation result only; no harmonized pooled effect. | Narrative/single-study display |
| Bahador 2022 | Diarrhea severity odds | National Cancer Institute scale | Events/participants | The reported OR direction was inferred as control/no selenium versus selenium; OR >1 therefore favors selenium for harmful toxicity, but the direction remains uncertain. | GI diarrhea OR-only; not mixed with RR counts |
| Bahador 2022 | Pain and abdominal cramp odds | Not clearly specified | Events/participants | The reported OR direction was inferred as control/no selenium versus selenium; OR >1 therefore favors selenium for harmful toxicity, but the direction remains uncertain. | Narrative/OR-only display |
| Pavlidis 2026 | Electrogustometry thresholds | Electrogustometry; LMM estimated marginal means | LMM estimated marginal means/pairwise comparisons | Narrative/model/correlation result only; no harmonized pooled effect. | Model-based single-study display; not OM RR or unadjusted MD pool |
| Pavlidis 2026 | fPap and blood-vessel morphology | Ordinal morphology types | Kendall tau-b correlations | Narrative/model/correlation result only; no harmonized pooled effect. | Narrative/single-study display |

*Note. Original outcome definitions and time points were preserved. Pooling required compatible effect measures and clinically defensible definitions; broader combinations were explicitly exploratory.*

**Supplementary Table S5. Final risk-of-bias judgments and support**

**Panel A. Domain and overall judgments**

| **Study** | **Tool** | **Domain** | **Judgment** | **Overall judgment** | **Figure link** |
| --- | --- | --- | --- | --- | --- |
| Yim 2026 | Final RoB 2 assessment for a parallel RCT | D1 randomization process | Low risk | Some concerns | Supplementary Figures S12-S13 |
| Yim 2026 | Final RoB 2 assessment for a parallel RCT | D2 deviations from intended interventions | Low risk | Some concerns | Supplementary Figures S12-S13 |
| Yim 2026 | Final RoB 2 assessment for a parallel RCT | D3 missing outcome data | Some concerns | Some concerns | Supplementary Figures S12-S13 |
| Yim 2026 | Final RoB 2 assessment for a parallel RCT | D4 measurement of outcome | Some concerns | Some concerns | Supplementary Figures S12-S13 |
| Yim 2026 | Final RoB 2 assessment for a parallel RCT | D5 selection of reported result | Some concerns | Some concerns | Supplementary Figures S12-S13 |
| Yim 2026 | Final RoB 2 assessment for a parallel RCT | Overall | Some concerns | Some concerns | Supplementary Figures S12-S13 |
| Yang 2023 | Final RoB 2 assessment for a parallel RCT | D1 randomization process | Low risk | Some concerns | Supplementary Figures S12-S13 |
| Yang 2023 | Final RoB 2 assessment for a parallel RCT | D2 deviations from intended interventions | Low risk | Some concerns | Supplementary Figures S12-S13 |
| Yang 2023 | Final RoB 2 assessment for a parallel RCT | D3 missing outcome data | Some concerns | Some concerns | Supplementary Figures S12-S13 |
| Yang 2023 | Final RoB 2 assessment for a parallel RCT | D4 measurement of outcome | Low risk | Some concerns | Supplementary Figures S12-S13 |
| Yang 2023 | Final RoB 2 assessment for a parallel RCT | D5 selection of reported result | Some concerns | Some concerns | Supplementary Figures S12-S13 |
| Yang 2023 | Final RoB 2 assessment for a parallel RCT | Overall | Some concerns | Some concerns | Supplementary Figures S12-S13 |
| Laali 2020 | Final RoB 2 assessment for a parallel RCT | D1 randomization process | Some concerns | Some concerns | Supplementary Figures S12-S13 |
| Laali 2020 | Final RoB 2 assessment for a parallel RCT | D2 deviations from intended interventions | Low risk | Some concerns | Supplementary Figures S12-S13 |
| Laali 2020 | Final RoB 2 assessment for a parallel RCT | D3 missing outcome data | Some concerns | Some concerns | Supplementary Figures S12-S13 |
| Laali 2020 | Final RoB 2 assessment for a parallel RCT | D4 measurement of outcome | Low risk | Some concerns | Supplementary Figures S12-S13 |
| Laali 2020 | Final RoB 2 assessment for a parallel RCT | D5 selection of reported result | Some concerns | Some concerns | Supplementary Figures S12-S13 |
| Laali 2020 | Final RoB 2 assessment for a parallel RCT | Overall | Some concerns | Some concerns | Supplementary Figures S12-S13 |
| Mix 2015 | Final RoB 2 assessment for a parallel RCT | D1 randomization process | Low risk | Some concerns | Supplementary Figures S12-S13 |
| Mix 2015 | Final RoB 2 assessment for a parallel RCT | D2 deviations from intended interventions | Some concerns | Some concerns | Supplementary Figures S12-S13 |
| Mix 2015 | Final RoB 2 assessment for a parallel RCT | D3 missing outcome data | Some concerns | Some concerns | Supplementary Figures S12-S13 |
| Mix 2015 | Final RoB 2 assessment for a parallel RCT | D4 measurement of outcome | Low risk | Some concerns | Supplementary Figures S12-S13 |
| Mix 2015 | Final RoB 2 assessment for a parallel RCT | D5 selection of reported result | Some concerns | Some concerns | Supplementary Figures S12-S13 |
| Mix 2015 | Final RoB 2 assessment for a parallel RCT | Overall | Some concerns | Some concerns | Supplementary Figures S12-S13 |
| Vieira 2015 | Final adapted RoB 2 assessment for a crossover trial | D1 randomization process | Some concerns | High risk | Supplementary Figures S12-S13 |
| Vieira 2015 | Final adapted RoB 2 assessment for a crossover trial | D2 deviations, period effects, and carryover | High risk | High risk | Supplementary Figures S12-S13 |
| Vieira 2015 | Final adapted RoB 2 assessment for a crossover trial | D3 missing outcome data | Some concerns | High risk | Supplementary Figures S12-S13 |
| Vieira 2015 | Final adapted RoB 2 assessment for a crossover trial | D4 measurement of outcome | Some concerns | High risk | Supplementary Figures S12-S13 |
| Vieira 2015 | Final adapted RoB 2 assessment for a crossover trial | D5 selection of reported result | Some concerns | High risk | Supplementary Figures S12-S13 |
| Vieira 2015 | Final adapted RoB 2 assessment for a crossover trial | Overall | High risk | High risk | Supplementary Figures S12-S13 |
| Jahangard-Rafsanjani 2013 | Final RoB 2 assessment for a parallel RCT | D1 randomization process | Some concerns | Some concerns | Supplementary Figures S12-S13 |
| Jahangard-Rafsanjani 2013 | Final RoB 2 assessment for a parallel RCT | D2 deviations from intended interventions | Low risk | Some concerns | Supplementary Figures S12-S13 |
| Jahangard-Rafsanjani 2013 | Final RoB 2 assessment for a parallel RCT | D3 missing outcome data | Some concerns | Some concerns | Supplementary Figures S12-S13 |
| Jahangard-Rafsanjani 2013 | Final RoB 2 assessment for a parallel RCT | D4 measurement of outcome | Low risk | Some concerns | Supplementary Figures S12-S13 |
| Jahangard-Rafsanjani 2013 | Final RoB 2 assessment for a parallel RCT | D5 selection of reported result | Some concerns | Some concerns | Supplementary Figures S12-S13 |
| Jahangard-Rafsanjani 2013 | Final RoB 2 assessment for a parallel RCT | Overall | Some concerns | Some concerns | Supplementary Figures S12-S13 |
| Ghorbani 2013 | Final RoB 2 assessment for a parallel RCT | D1 randomization process | Low risk | Some concerns | Supplementary Figures S12-S13 |
| Ghorbani 2013 | Final RoB 2 assessment for a parallel RCT | D2 deviations from intended interventions | Low risk | Some concerns | Supplementary Figures S12-S13 |
| Ghorbani 2013 | Final RoB 2 assessment for a parallel RCT | D3 missing outcome data | Some concerns | Some concerns | Supplementary Figures S12-S13 |
| Ghorbani 2013 | Final RoB 2 assessment for a parallel RCT | D4 measurement of outcome | Low risk | Some concerns | Supplementary Figures S12-S13 |
| Ghorbani 2013 | Final RoB 2 assessment for a parallel RCT | D5 selection of reported result | Some concerns | Some concerns | Supplementary Figures S12-S13 |
| Ghorbani 2013 | Final RoB 2 assessment for a parallel RCT | Overall | Some concerns | Some concerns | Supplementary Figures S12-S13 |
| Muecke 2010 | Final RoB 2 assessment for a parallel RCT | D1 randomization process | Some concerns | High risk | Supplementary Figures S12-S13 |
| Muecke 2010 | Final RoB 2 assessment for a parallel RCT | D2 deviations from intended interventions | Some concerns | High risk | Supplementary Figures S12-S13 |
| Muecke 2010 | Final RoB 2 assessment for a parallel RCT | D3 missing outcome data | Low risk | High risk | Supplementary Figures S12-S13 |
| Muecke 2010 | Final RoB 2 assessment for a parallel RCT | D4 measurement of outcome | High risk | High risk | Supplementary Figures S12-S13 |
| Muecke 2010 | Final RoB 2 assessment for a parallel RCT | D5 selection of reported result | Some concerns | High risk | Supplementary Figures S12-S13 |
| Muecke 2010 | Final RoB 2 assessment for a parallel RCT | Overall | High risk | High risk | Supplementary Figures S12-S13 |
| Buentzel 2010 | Final RoB 2 assessment for a parallel RCT | D1 randomization process | Some concerns | High risk | Supplementary Figures S12-S13 |
| Buentzel 2010 | Final RoB 2 assessment for a parallel RCT | D2 deviations from intended interventions | High risk | High risk | Supplementary Figures S12-S13 |
| Buentzel 2010 | Final RoB 2 assessment for a parallel RCT | D3 missing outcome data | Some concerns | High risk | Supplementary Figures S12-S13 |
| Buentzel 2010 | Final RoB 2 assessment for a parallel RCT | D4 measurement of outcome | High risk | High risk | Supplementary Figures S12-S13 |
| Buentzel 2010 | Final RoB 2 assessment for a parallel RCT | D5 selection of reported result | Some concerns | High risk | Supplementary Figures S12-S13 |
| Buentzel 2010 | Final RoB 2 assessment for a parallel RCT | Overall | High risk | High risk | Supplementary Figures S12-S13 |
| Asfour 2006 | Final RoB 2 assessment for a parallel RCT | D1 randomization process | Some concerns | High risk | Supplementary Figures S12-S13 |
| Asfour 2006 | Final RoB 2 assessment for a parallel RCT | D2 deviations from intended interventions | High risk | High risk | Supplementary Figures S12-S13 |
| Asfour 2006 | Final RoB 2 assessment for a parallel RCT | D3 missing outcome data | Low risk | High risk | Supplementary Figures S12-S13 |
| Asfour 2006 | Final RoB 2 assessment for a parallel RCT | D4 measurement of outcome | High risk | High risk | Supplementary Figures S12-S13 |
| Asfour 2006 | Final RoB 2 assessment for a parallel RCT | D5 selection of reported result | Some concerns | High risk | Supplementary Figures S12-S13 |
| Asfour 2006 | Final RoB 2 assessment for a parallel RCT | Overall | High risk | High risk | Supplementary Figures S12-S13 |
| Hu 1997 | Final adapted RoB 2 assessment for a crossover trial | D1 randomization process | Some concerns | High risk | Supplementary Figures S12-S13 |
| Hu 1997 | Final adapted RoB 2 assessment for a crossover trial | D2 deviations, period effects, and carryover | High risk | High risk | Supplementary Figures S12-S13 |
| Hu 1997 | Final adapted RoB 2 assessment for a crossover trial | D3 missing outcome data | Some concerns | High risk | Supplementary Figures S12-S13 |
| Hu 1997 | Final adapted RoB 2 assessment for a crossover trial | D4 measurement of outcome | Some concerns | High risk | Supplementary Figures S12-S13 |
| Hu 1997 | Final adapted RoB 2 assessment for a crossover trial | D5 selection of reported result | Some concerns | High risk | Supplementary Figures S12-S13 |
| Hu 1997 | Final adapted RoB 2 assessment for a crossover trial | Overall | High risk | High risk | Supplementary Figures S12-S13 |
| Ameri 2016 | Final RoB 2 assessment for a parallel RCT | D1 randomization process | Some concerns | High risk | Supplementary Figures S12-S13 |
| Ameri 2016 | Final RoB 2 assessment for a parallel RCT | D2 deviations from intended interventions | Some concerns | High risk | Supplementary Figures S12-S13 |
| Ameri 2016 | Final RoB 2 assessment for a parallel RCT | D3 missing outcome data | High risk | High risk | Supplementary Figures S12-S13 |
| Ameri 2016 | Final RoB 2 assessment for a parallel RCT | D4 measurement of outcome | Some concerns | High risk | Supplementary Figures S12-S13 |
| Ameri 2016 | Final RoB 2 assessment for a parallel RCT | D5 selection of reported result | Some concerns | High risk | Supplementary Figures S12-S13 |
| Ameri 2016 | Final RoB 2 assessment for a parallel RCT | Overall | High risk | High risk | Supplementary Figures S12-S13 |
| Chen 2023 | Final RoB 2 assessment for a parallel clinical trial | D1 randomization process | Some concerns | Some concerns | Supplementary Figures S12-S13 |
| Chen 2023 | Final RoB 2 assessment for a parallel clinical trial | D2 deviations from intended interventions | Some concerns | Some concerns | Supplementary Figures S12-S13 |
| Chen 2023 | Final RoB 2 assessment for a parallel clinical trial | D3 missing outcome data | Low risk | Some concerns | Supplementary Figures S12-S13 |
| Chen 2023 | Final RoB 2 assessment for a parallel clinical trial | D4 measurement of outcome | Some concerns | Some concerns | Supplementary Figures S12-S13 |
| Chen 2023 | Final RoB 2 assessment for a parallel clinical trial | D5 selection of reported result | Some concerns | Some concerns | Supplementary Figures S12-S13 |
| Chen 2023 | Final RoB 2 assessment for a parallel clinical trial | Overall | Some concerns | Some concerns | Supplementary Figures S12-S13 |
| Wang 2020 | Final RoB 2 assessment for a parallel clinical trial | D1 randomization process | Some concerns | Some concerns | Supplementary Figures S12-S13 |
| Wang 2020 | Final RoB 2 assessment for a parallel clinical trial | D2 deviations from intended interventions | Some concerns | Some concerns | Supplementary Figures S12-S13 |
| Wang 2020 | Final RoB 2 assessment for a parallel clinical trial | D3 missing outcome data | Low risk | Some concerns | Supplementary Figures S12-S13 |
| Wang 2020 | Final RoB 2 assessment for a parallel clinical trial | D4 measurement of outcome | Some concerns | Some concerns | Supplementary Figures S12-S13 |
| Wang 2020 | Final RoB 2 assessment for a parallel clinical trial | D5 selection of reported result | Some concerns | Some concerns | Supplementary Figures S12-S13 |
| Wang 2020 | Final RoB 2 assessment for a parallel clinical trial | Overall | Some concerns | Some concerns | Supplementary Figures S12-S13 |
| Son 2017 | Final RoB 2 assessment for a parallel clinical trial | D1 randomization process | Some concerns | Some concerns | Supplementary Figures S12-S13 |
| Son 2017 | Final RoB 2 assessment for a parallel clinical trial | D2 deviations from intended interventions | Some concerns | Some concerns | Supplementary Figures S12-S13 |
| Son 2017 | Final RoB 2 assessment for a parallel clinical trial | D3 missing outcome data | Low risk | Some concerns | Supplementary Figures S12-S13 |
| Son 2017 | Final RoB 2 assessment for a parallel clinical trial | D4 measurement of outcome | Some concerns | Some concerns | Supplementary Figures S12-S13 |
| Son 2017 | Final RoB 2 assessment for a parallel clinical trial | D5 selection of reported result | Some concerns | Some concerns | Supplementary Figures S12-S13 |
| Son 2017 | Final RoB 2 assessment for a parallel clinical trial | Overall | Some concerns | Some concerns | Supplementary Figures S12-S13 |
| Bahador 2022 | Final RoB 2 assessment for a parallel clinical trial | D1 randomization process | Some concerns | High risk | Supplementary Figures S12-S13 |
| Bahador 2022 | Final RoB 2 assessment for a parallel clinical trial | D2 deviations from intended interventions | Some concerns | High risk | Supplementary Figures S12-S13 |
| Bahador 2022 | Final RoB 2 assessment for a parallel clinical trial | D3 missing outcome data | Low risk | High risk | Supplementary Figures S12-S13 |
| Bahador 2022 | Final RoB 2 assessment for a parallel clinical trial | D4 measurement of outcome | Some concerns | High risk | Supplementary Figures S12-S13 |
| Bahador 2022 | Final RoB 2 assessment for a parallel clinical trial | D5 selection of reported result | High risk | High risk | Supplementary Figures S12-S13 |
| Bahador 2022 | Final RoB 2 assessment for a parallel clinical trial | Overall | High risk | High risk | Supplementary Figures S12-S13 |
| Pavlidis 2026 | Final RoB 2 assessment for a parallel clinical trial | D1 randomization process | Some concerns | High risk | Supplementary Figures S12-S13 |
| Pavlidis 2026 | Final RoB 2 assessment for a parallel clinical trial | D2 deviations from intended interventions | High risk | High risk | Supplementary Figures S12-S13 |
| Pavlidis 2026 | Final RoB 2 assessment for a parallel clinical trial | D3 missing outcome data | Some concerns | High risk | Supplementary Figures S12-S13 |
| Pavlidis 2026 | Final RoB 2 assessment for a parallel clinical trial | D4 measurement of outcome | Some concerns | High risk | Supplementary Figures S12-S13 |
| Pavlidis 2026 | Final RoB 2 assessment for a parallel clinical trial | D5 selection of reported result | High risk | High risk | Supplementary Figures S12-S13 |
| Pavlidis 2026 | Final RoB 2 assessment for a parallel clinical trial | Overall | High risk | High risk | Supplementary Figures S12-S13 |

**Panel B. Support for judgment**

| **Study** | **Domain** | **Support for judgment** |
| --- | --- | --- |
| Yim 2026 | D1 randomization process | 1:1 randomization used a reproducible web-based program and third-party allocation management. |
| Yim 2026 | D2 deviations from intended interventions | Double-blind placebo-controlled design; control received normal saline on the same chemotherapy schedule. |
| Yim 2026 | D3 missing outcome data | Efficacy analyses used PP population while adverse events used ITT; primary endpoint denominator needs confirmation. |
| Yim 2026 | D4 measurement of outcome | CIPN was graded using combined WHO-CIPN and NCI-CTCAE criteria during scheduled visits. |
| Yim 2026 | D5 selection of reported result | Trial registration was reported; multiple CIPN endpoints and time points were analyzed. |
| Yim 2026 | Overall | Design is strong, but missing-data/denominator and outcome-definition issues remain. |
| Yang 2023 | D1 randomization process | Randomization used a computer-generated random-number table in a 1:1 double-blind trial. |
| Yang 2023 | D2 deviations from intended interventions | Patients and investigators were blinded; placebo was pharmacy prepared and CCRT schedule was common. |
| Yang 2023 | D3 missing outcome data | 124 were randomized and 104 analyzed; 20 patients were excluded or withdrew. |
| Yang 2023 | D4 measurement of outcome | Adverse events were graded with NCI Common Toxicity Criteria version 3.0 in a blinded trial. |
| Yang 2023 | D5 selection of reported result | Multiple toxicity and response outcomes were reported; exact registry identifier/SAP was not extracted. |
| Yang 2023 | Overall | Randomization and blinding are credible, but attrition and reporting-prespecification require review. |
| Laali 2020 | D1 randomization process | Blocked randomization was reported, but sequence generation and concealment details were limited. |
| Laali 2020 | D2 deviations from intended interventions | Double-blind placebo-controlled design; selenium and placebo were administered through RT. |
| Laali 2020 | D3 missing outcome data | 84 were recruited and 71 analyzed; dropouts after RT initiation were reported. |
| Laali 2020 | D4 measurement of outcome | OM was assessed weekly using WHO oral toxicity grading by blinded assessors. |
| Laali 2020 | D5 selection of reported result | Several OM measures and time points were reported; no protocol/SAP was extracted. |
| Laali 2020 | Overall | Blinding and outcome measurement are good, but randomization detail, attrition, and reporting require review. |
| Mix 2015 | D1 randomization process | Block randomization by site was reported in a multicenter double-blind placebo-controlled trial. |
| Mix 2015 | D2 deviations from intended interventions | One SLM patient received one dose and abandoned treatment; trial stopped after interim review. |
| Mix 2015 | D3 missing outcome data | Only 18 patients were randomized/analyzed after a planned 80-patient trial stopped early. |
| Mix 2015 | D4 measurement of outcome | Double-blinding and prespecified mucositis/toxicity grading reduce measurement concern. |
| Mix 2015 | D5 selection of reported result | The trial had planned interim analyses and was stopped early; multiple toxicity/QoL outcomes were reported. |
| Mix 2015 | Overall | Strong RCT design but very small stopped trial and adherence/attrition issues remain. |
| Vieira 2015 | D1 randomization process | Randomized double-blind crossover design was reported, but sequence generation/concealment was not clearly reported. |
| Vieira 2015 | D2 deviations, period effects, and carryover | Crossover periods were used and some chemotherapy protocols changed; no washout/carryover handling was extracted. |
| Vieira 2015 | D3 missing outcome data | Only 39 were included after 74 invited; protocol break, spontaneous dropout, or death were exclusion triggers. |
| Vieira 2015 | D4 measurement of outcome | QoL/symptom measures were patient-reported in a blinded crossover trial. |
| Vieira 2015 | D5 selection of reported result | Multiple symptoms, labs, periods, and follow-up time points were reported without extracted protocol/SAP. |
| Vieira 2015 | Overall | Crossover/carryover and changing chemotherapy-protocol context make causal synthesis high risk. |
| Jahangard-Rafsanjani 2013 | D1 randomization process | Blocked randomization was reported, but sequence generation and concealment details were not fully described. |
| Jahangard-Rafsanjani 2013 | D2 deviations from intended interventions | Double-blind placebo-controlled design; attending physician was blinded and supportive care was similar. |
| Jahangard-Rafsanjani 2013 | D3 missing outcome data | 77 randomized; 74 completed and tables report 37 per group. |
| Jahangard-Rafsanjani 2013 | D4 measurement of outcome | OM was assessed daily using the WHO oral toxicity scale by a blinded clinical team. |
| Jahangard-Rafsanjani 2013 | D5 selection of reported result | Multiple OM, renal/hepatic, selenium, and enzyme outcomes were reported; protocol/SAP not extracted. |
| Jahangard-Rafsanjani 2013 | Overall | Blinded RCT with explicit OM outcome, but randomization detail, attrition, and reporting need review. |
| Ghorbani 2013 | D1 randomization process | Random-number list and sealed opaque envelopes were reported. |
| Ghorbani 2013 | D2 deviations from intended interventions | Investigators, patients, and analyzers were blinded; placebo matched selenium in appearance/taste. |
| Ghorbani 2013 | D3 missing outcome data | 11 of 133 patients were lost to follow-up and excluded; 122 were analyzed. |
| Ghorbani 2013 | D4 measurement of outcome | Primary AKI endpoint used plasma creatinine and urine-flow criteria, with blinded analyzers. |
| Ghorbani 2013 | D5 selection of reported result | Primary endpoints were clear, but trial registration/SAP was not extracted and outcome measured at one day-5 window. |
| Ghorbani 2013 | Overall | Strong blinding/randomization and objective endpoint, but excluded losses and reporting prespecification need review. |
| Muecke 2010 | D1 randomization process | Randomization and data monitoring were handled independently, but sequence/concealment details were limited. |
| Muecke 2010 | D2 deviations from intended interventions | Comparator was observation/no selenium rather than placebo, so participants and clinicians were likely aware. |
| Muecke 2010 | D3 missing outcome data | 81 patients were randomized and 81 appear represented in the analysis tables. |
| Muecke 2010 | D4 measurement of outcome | Diarrhea was graded by CTC, but the no-placebo design makes outcome assessment vulnerable to awareness of intervention. |
| Muecke 2010 | D5 selection of reported result | Primary endpoint was selenium level; diarrhea was a secondary endpoint and companion reports exist. |
| Muecke 2010 | Overall | Observation control and unblinded toxicity measurement create high concern for subjective diarrhea outcomes. |
| Buentzel 2010 | D1 randomization process | Randomization was performed, but method and concealment were not described in detail. |
| Buentzel 2010 | D2 deviations from intended interventions | Control received radiotherapy without selenium; patients were informed about treatment arm. |
| Buentzel 2010 | D3 missing outcome data | 40 were randomized and 39 analyzed; pre-randomization withdrawal and one missing randomized patient require checking. |
| Buentzel 2010 | D4 measurement of outcome | RTOG toxicities were semi-quantitative; authors noted possible inconsistency between individual evaluations. |
| Buentzel 2010 | D5 selection of reported result | Multiple weekly and maximal RTOG toxicities were analyzed in an underpowered phase II study. |
| Buentzel 2010 | Overall | Open comparator and semi-quantitative toxicity assessment make bias concerns high. |
| Asfour 2006 | D1 randomization process | Patients were randomly divided into two groups, but sequence generation and concealment were not described. |
| Asfour 2006 | D2 deviations from intended interventions | Comparator was chemotherapy alone; no placebo or blinding evidence was extracted. |
| Asfour 2006 | D3 missing outcome data | Thirty patients were randomized with 15 per group, and no attrition was extracted. |
| Asfour 2006 | D4 measurement of outcome | Infection counts were figure/percentage based and inferred; no blinded assessment evidence was extracted. |
| Asfour 2006 | D5 selection of reported result | Mechanistic, infection, cardiac, and toxicity outcomes were reported without extracted protocol/SAP. |
| Asfour 2006 | Overall | Unclear randomization, open comparator, and uncertain outcome ascertainment create high concern. |
| Hu 1997 | D1 randomization process | Patients were randomized into group A/B for a crossover design, but sequence generation/concealment were not reported. |
| Hu 1997 | D2 deviations, period effects, and carryover | Crossover/cycle design was used around chemotherapy cycles without extracted washout or carryover handling. |
| Hu 1997 | D3 missing outcome data | Forty-one patients/cycles were reported, but within-patient correlation and cycle-level missingness were not extracted. |
| Hu 1997 | D4 measurement of outcome | Many endpoints were objective labs, but blinding and standardized assessor procedures were not extracted. |
| Hu 1997 | D5 selection of reported result | Numerous lab biomarkers and post-cisplatin time points were reported without extracted protocol/SAP. |
| Hu 1997 | Overall | Crossover period/carryover and unclear reporting prevent direct primary pooling. |
| Ameri 2016 | D1 randomization process | English abstract reports random division, but detailed sequence/concealment requires Persian-text audit. |
| Ameri 2016 | D2 deviations from intended interventions | Double-blind placebo design was reported, but intervention adherence and comparator details need translation confirmation. |
| Ameri 2016 | D3 missing outcome data | 35 patients were enrolled, but group denominators and attrition were not fully extractable from English text. |
| Ameri 2016 | D4 measurement of outcome | Weekly mucosal evaluation used NCI-CTC version 2 and Epstein scoring, but assessor blinding details are unresolved. |
| Ameri 2016 | D5 selection of reported result | Repeated-measures ANOVA and week-specific scores were reported; protocol/SAP and denominators unresolved. |
| Ameri 2016 | Overall | Translation-sensitive methods and unconfirmed denominators make current RoB support high concern. |
| Chen 2023 | D1 randomization process | The article states that 86 patients were randomized 43/43, but sequence generation and allocation concealment details were not extracted. |
| Chen 2023 | D2 deviations from intended interventions | Both groups received platinum-containing chemotherapy, but the control group had no selenium placebo and blinding was not reported. |
| Chen 2023 | D3 missing outcome data | The extracted report retains 43 participants in each arm for adverse reaction and pain tables, with no missing outcome data signal identified. |
| Chen 2023 | D4 measurement of outcome | Adverse reactions were graded using WHO chemotherapy adverse-reaction classification; pain was scored by attending/chief physicians, but assessor blinding was not reported and one adverse-reaction percentage is inconsistent. |
| Chen 2023 | D5 selection of reported result | No registry or SAP was extracted; several toxicity, appetite, weight, KPS, pain, and response outcomes were reported, and Table V contains an internal inconsistency. |
| Chen 2023 | Overall | The trial is randomized and has complete arm-level outcome tables, but randomization concealment, blinding, subjective outcome measurement, and table inconsistency remain unresolved. |
| Wang 2020 | D1 randomization process | Patients were assigned by random-number table to selenium yeast or routine treatment groups, but allocation concealment was not reported. |
| Wang 2020 | D2 deviations from intended interventions | Both groups received CCRT and Kangfuxin, while selenium was added only in the intervention arm; no placebo or participant/personnel blinding was reported. |
| Wang 2020 | D3 missing outcome data | The study randomized 60 patients into two groups of 30 and outcome tables report n=30 for both groups, with no attrition signal extracted. |
| Wang 2020 | D4 measurement of outcome | Oral mucositis used RTOG criteria and pain used a defined descriptive scale assessed daily by physicians, but assessor blinding was not reported. |
| Wang 2020 | D5 selection of reported result | No registry or SAP was extracted; multiple outcomes were reported, including OM onset, grade, pain, relief time, and short-term tumor response. |
| Wang 2020 | Overall | Randomization and complete outcome tables are clear, but concealment, placebo/blinding, and prespecification remain unresolved. |
| Son 2017 | D1 randomization process | The study is described as single-blind randomized prospective and allocated 8 patients per arm, but sequence generation and allocation concealment details were not extracted. |
| Son 2017 | D2 deviations from intended interventions | The selenium group received sodium selenite and the control group received oral placebo for the same duration, but single-blind wording leaves personnel/outcome-assessor blinding unclear. |
| Son 2017 | D3 missing outcome data | All 16 patients are summarized in baseline and follow-up tables; no attrition signal was extracted. |
| Son 2017 | D4 measurement of outcome | Serum amylase and scintigraphy are objective, and two nuclear medicine physicians performed quantitative analysis, but symptom questionnaire outcomes are subjective and assessor blinding is unclear. |
| Son 2017 | D5 selection of reported result | No registry or SAP was extracted; multiple salivary gland, biomarker, and questionnaire endpoints were reported with mostly within-arm testing. |
| Son 2017 | Overall | Placebo control and objective measures strengthen the study, but randomization/concealment, single-blind status, small sample, and reporting prespecification remain unclear. |
| Bahador 2022 | D1 randomization process | The study used block randomization with block size 4 and assigned 40 patients to each group, but allocation concealment was not reported and baseline sex imbalance is possible. |
| Bahador 2022 | D2 deviations from intended interventions | The control group received distilled water placebo, but blinding was not explicitly reported; treatment regimens varied by pelvic cancer type. |
| Bahador 2022 | D3 missing outcome data | No patients were lost to follow-up and all patients received the prescribed intervention. |
| Bahador 2022 | D4 measurement of outcome | Adverse effects were assessed clinically using the NCI scale, but blinding of patients/outcome assessors is unclear and GI symptoms are partly subjective. |
| Bahador 2022 | D5 selection of reported result | No registry/SAP was extracted; the article reports GEE ORs without raw arm-level counts and contains unclear table alignment/p-value reporting. |
| Bahador 2022 | Overall | Randomization and follow-up are reported, but heterogeneous regimens, unclear blinding, subjective toxicity assessment, and incomplete OR-only reporting create serious concerns. |
| Pavlidis 2026 | D1 randomization process | Fifty-one patients were randomly allocated into three groups of 17, but sequence generation and allocation concealment were not reported in extracted text. |
| Pavlidis 2026 | D2 deviations from intended interventions | The control group received no placebo while the discussion describes a double-blind randomized controlled design, creating an unresolved design contradiction. |
| Pavlidis 2026 | D3 missing outcome data | The abstract reports 17 per group, but extracted intervention text appears internally inconsistent for sex totals in zinc/selenium groups, and attrition/analyzed counts are not fully clear. |
| Pavlidis 2026 | D4 measurement of outcome | EGM uses an instrumented threshold method and CE morphology classifications, but outcome assessment was carried out by one clinician and blinding is unclear. |
| Pavlidis 2026 | D5 selection of reported result | No registry/SAP was extracted; the paper emphasizes severe OM but extractable arm-level OM event counts were not available, and model/table reporting was incomplete in text extraction. |
| Pavlidis 2026 | Overall | Randomized allocation is reported, but no-placebo control, conflicting blinding claims, uncertain analyzed n, and incomplete clinically poolable outcome reporting create serious concerns. |

Note. Judgments are final RoB 2-style assessments. The crossover-specific D2 domain was used for crossover trials. Visual summaries are Supplementary Figures S12-S13.

Supplementary Table S6. Effect extraction, processing, and direction harmonization

Panel A1. Raw effect data

| **Study** | **Outcome** | **Time point** | **Group or contrast** | **Reported metric** | **Raw values** |
| --- | --- | --- | --- | --- | --- |
| Yim 2026 | Grade 1 or more CIPN/paresthesia as reported (Grade >=1) | 3 months post-chemotherapy | Experimental selenium versus Control placebo | Arm-level event counts | Experimental selenium: 19/26; Control placebo: 24/30 |
| Yim 2026 | Grade 2 or more motor dysfunction (Grade >=2) | Before cycle 3 | Placebo versus Selenium | Arm-level event counts | Placebo: 7/30; Selenium: 1/30 |
| Yim 2026 | Grade 2 or more motor dysfunction (Grade >=2) | Before cycle 4 | Placebo versus Selenium | Arm-level event counts | Placebo: 6/30; Selenium: 1/30 |
| Yim 2026 | Any grade adverse event (Any grade) | During treatment | Placebo versus Selenium | Arm-level event counts | Placebo: 8/34; Selenium: 7/34 |
| Yim 2026 | CIPN20 sensory score (Continuous score) | 3 months after chemotherapy | Selenium versus Placebo | Continuous mean and SD | Selenium: mean 33.19, SD 24.10; Placebo: mean 27.41, SD 20.51 |
| Yang 2023 | Myelosuppression (Grade 3) | During CCRT | Experimental selenium versus Control placebo | Arm-level event counts | Experimental selenium: 24/50; Control placebo: 37/54 |
| Yang 2023 | Myelosuppression (Grade 4) | During CCRT | Experimental selenium versus Control placebo | Arm-level event counts | Experimental selenium: 4/50; Control placebo: 3/54 |
| Yang 2023 | Diarrhea (Any diarrhea as table row) | During CCRT | Experimental selenium versus Control placebo | Arm-level event counts | Experimental selenium: 5/50; Control placebo: 4/54 |
| Yang 2023 | Whole blood selenium level (continuous) | End of RT | Experimental selenium versus Control placebo | Mean and SD | Experimental selenium: mean 76.16, SD 24.47; Control placebo: mean 57.48, SD 14.92 |
| Laali 2020 | Cumulative OM incidence (Grade 1-4) | During and after RT | Selenium versus Placebo | Arm-level event counts | Selenium: 36/37; Placebo: 34/34 |
| Laali 2020 | Severe OM (Grade 3-4) | During RT | Selenium versus Placebo | Arm-level event counts | Selenium: 25/37; Placebo: 20/34 |
| Laali 2020 | Duration of OM (Grade 1-4 duration) | During RT | Selenium versus Placebo | Mean and SD | Selenium: mean 46.97, SD 20.26; Placebo: mean 50.44, SD 17.56 |
| Laali 2020 | Oral mucositis onset time (Continuous weeks; higher is favorable) | During radiotherapy | Selenium versus Placebo | Mean and SD | Selenium: mean 1.61, SD 0.83; Placebo: mean 1.70, SD 1.05 |
| Laali 2020 | Grade 3 neutropenia (Grade 3) | Concurrent chemoradiation subgroup | Selenium subgroup versus Placebo subgroup | Arm-level event counts | Selenium subgroup: 2/18; Placebo subgroup: 3/16 |
| Mix 2015 | Severe mucositis (Grade 3-4) | During CRT | Placebo versus SLM | Arm-level event counts | Placebo: 3/8; SLM: 2/10 |
| Mix 2015 | Leukopenia grade >=2 (Grade >=2) | During CRT | Placebo versus SLM | Arm-level event counts | Placebo: 2/8; SLM: 3/10 |
| Vieira 2015 | Solid tumor nausea score (Continuous score; median and quartiles) | Initial vs placebo vs selenium crossover periods | Placebo period versus Selenium period | Median and IQR | Placebo period: Median 0; Q25 0; Q75 25; Selenium period: Median 0; Q25 0; Q75 0 |
| Vieira 2015 | AST all patients (Continuous laboratory value) | Placebo vs selenium period | Placebo period versus Selenium period | Median and range | Placebo period: Median 31; min 17; max 63; Selenium period: Median 24; min 8; max 52 |
| Jahangard-Rafsanjani 2013 | Any oral mucositis (WHO grade 1-4) | Post-HDC/HSCT assessment window | Selenium versus Control | Arm-level event counts | Selenium: 31/37; Control: 30/37 |
| Jahangard-Rafsanjani 2013 | Severe oral mucositis (WHO grade 3-4) | Post-HDC/HSCT assessment window | Selenium versus Control | Arm-level event counts | Selenium: 4/37; Control: 13/37 |
| Jahangard-Rafsanjani 2013 | Duration of grade 2-4 oral mucositis (WHO grade 2-4 duration) | Post-HDC/HSCT assessment window | Selenium versus Control | Mean and SD | Selenium: mean 3.6, SD 1.84; Control: mean 5.3, SD 2.2 |
| Jahangard-Rafsanjani 2013 | Serum creatinine elevation (Trial-defined elevation) | During hospitalization | Selenium versus Control | Arm-level event counts | Selenium: 3/37; Control: 7/37 |
| Ghorbani 2013 | Acute kidney injury (Trial-defined AKI) | Day 5 after cisplatin | Selenium versus Placebo | Arm-level event counts | Selenium: 0/61; Placebo: 7/61 |
| Muecke 2010 | CTC grade 1-3 diarrhea (CTC grade 1-3) | Week 4 of RT | With selenium versus Without selenium | Arm-level event counts | With selenium: 12/39; Without selenium: 25/42 |
| Muecke 2010 | CTC grade 2 or higher diarrhea (CTC grade >=2) | Actuarial incidence during RT | With selenium versus Without selenium | Reported percentage | With selenium: 20.5% actuarial incidence; Without selenium: 44.5% actuarial incidence |
| Buentzel 2010 | Maximal stomatitis (RTOG grade >=3) | Maximal during RT | Selenium versus Control | Arm-level event counts | Selenium: 8/22; Control: 4/17 |
| Buentzel 2010 | Maximal dysphagia (RTOG grade >=3) | Maximal during RT | Selenium versus Control | Arm-level event counts | Selenium: 5/22; Control: 6/17 |
| Asfour 2006 | Infection rate (Infection yes/no) | Following chemotherapy | Chemotherapy control versus Chemotherapy plus sodium selenite | Event count inferred from reported percentages | Chemotherapy control: 10/15; Chemotherapy plus sodium selenite: 3/15 |
| Asfour 2006 | Cardiac ejection fraction (Continuous EF %) | Following sodium selenite administration | Before/initial selenium group versus After selenium administration | Mean and SD within group | Before/initial selenium group: mean 62, SD 4; After selenium administration: mean 69, SD 6 |
| Hu 1997 | Peripheral WBC count (Continuous WBC x10^9/L) | Day 14 after chemotherapy initiation | Se-supplementation cycle versus Control cycle | Mean and SD | Se-supplementation cycle: mean 3.35, SD 2.01; Control cycle: mean 2.31, SD 1.38 |
| Hu 1997 | GCSF consumption (Continuous IU) | After cisplatin-contained chemotherapy | Se-supplementation cycle versus Control cycle | Mean and SD | Se-supplementation cycle: mean 110, SD 82; Control cycle: mean 723, SD 192 |
| Hu 1997 | Urine NAG (Continuous urine enzyme units/L) | 2 hours after cisplatin | Se-supplementation cycle versus Control cycle | Mean and SD | Se-supplementation cycle: mean 9.56, SD 8.71; Control cycle: mean 16.24, SD 7.33 |
| Hu 1997 | Urine GGT (Continuous urine enzyme units/L) | 24 hours after cisplatin | Se-supplementation cycle versus Control cycle | Mean and SD | Se-supplementation cycle: mean 17.18, SD 8.42; Control cycle: mean 27.24, SD 12.72 |
| Ameri 2016 | Mean mucositis score (Continuous mucositis score) | Week 1 | Selenium versus Placebo | Mean and SD | Selenium: mean 0.51, SD 0.28; Placebo: mean 1.21, SD 0.81 |
| Ameri 2016 | Mean mucositis score (Continuous mucositis score) | Week 2 | Selenium versus Placebo | Mean and SD | Selenium: mean 1.35, SD 0.55; Placebo: mean 1.79, SD 0.43 |
| Ameri 2016 | Mean mucositis score (Continuous mucositis score) | Week 3 | Selenium versus Placebo | Mean and SD | Selenium: mean 1.75, SD 0.34; Placebo: mean 2.08, SD 0.46 |
| Chen 2023 | Any adverse reaction grade I-IV (Any grade I-IV) | After 1 month/treatment course | Observation group versus Control group | None reported | Observation group: 15/43; Control group: 25/43 |
| Chen 2023 | Grade III-IV adverse reactions (Grade III-IV) | After 1 month/treatment course | Observation group versus Control group | None reported | Observation group: 0/43; Control group: 3/43 |
| Chen 2023 | Gastrointestinal reactions (Any grade) | After 1 month/treatment course | Observation group versus Control group | None reported | Observation group: 13/43; Control group: 21/43 |
| Chen 2023 | Bone marrow suppression (Any grade) | After 1 month/treatment course | Observation group versus Control group | None reported | Observation group: 1/43; Control group: 4/43 |
| Chen 2023 | Pain grade >=2 (Grade >=2) | During treatment | Observation group versus Control group | None reported | Observation group: 31/43; Control group: 38/43 |
| Wang 2020 | Severe oral mucositis (Grade >=3) | During concurrent chemoradiotherapy | Selenium yeast group versus Routine treatment group | None reported | Selenium yeast group: 5/30; Routine treatment group: 12/30 |
| Wang 2020 | Oral mucositis onset time (Continuous days; higher is favorable) | During concurrent chemoradiotherapy | Selenium yeast group versus Routine treatment group | None reported | Selenium yeast group: mean 13.1, SD 3.2; Routine treatment group: mean 11.7, SD 3.1 |
| Wang 2020 | Pain grade >=2 (Grade >=2) | During concurrent chemoradiotherapy | Selenium yeast group versus Routine treatment group | None reported | Selenium yeast group: 10/30; Routine treatment group: 18/30 |
| Wang 2020 | Pain relief time (Continuous days; lower is favorable) | During concurrent chemoradiotherapy | Selenium yeast group versus Routine treatment group | None reported | Selenium yeast group: mean 8.4, SD 3.9; Routine treatment group: mean 10.3, SD 4.3 |
| Son 2017 | Serum amylase 2 days after I-131 (Higher is worse) | 2 days after I-131 | Selenium supplement group versus Control group | None reported | Selenium supplement group: Median 139 IU/L (range 100-178); Control group: Median 296 IU/L (range 126-371) |
| Son 2017 | Questionnaire score 6 months after I-131 (Higher is worse) | 6 months after I-131 | Selenium supplement group versus Control group | None reported | Selenium supplement group: Median 18.5 (range 14-24); Control group: Median 23 (range 15-28) |
| Son 2017 | Salivary gland scintigraphy parameters () | 6 months after I-131 | Selenium versus Control | Within-arm Wilcoxon comparisons; no direct between-arm effect size | Selenium: Selenium group had significant decreases only in MSP right submandibular and EF left submandibular; Control: Control group had significant decreases in bilateral parotid MUR/MSP and left submandibular MUR/MSP/EF |
| Bahador 2022 | Diarrhea grade II/III vs grade 0 (Grade II/III to grade 0) | During and after chemoradiotherapy | Control/no selenium versus Selenium | GEE OR | Control/no selenium: Not reported; Selenium: Not reported |
| Bahador 2022 | Diarrhea grade II/III vs grade I (Grade II/III to grade I) | During and after chemoradiotherapy | Control/no selenium versus Selenium | GEE OR | Control/no selenium: Not reported; Selenium: Not reported |
| Bahador 2022 | Diarrhea grade I vs grade 0 (Grade I to grade 0) | During and after chemoradiotherapy | Control/no selenium versus Selenium | GEE OR | Control/no selenium: Not reported; Selenium: Not reported |
| Bahador 2022 | Pain (Not clearly specified) | During and after chemoradiotherapy | Control/no selenium versus Selenium | GEE OR | Control/no selenium: Not reported; Selenium: Not reported |
| Pavlidis 2026 | Electrogustometry thresholds (Continuous dB; lower is favorable) | Serial assessments A-F | Selenium versus Control | LMM EMMs and Sidak-adjusted pairwise comparisons | Selenium: EMMs: A 30.706; B 28.941; C 24.847; D 25.718; Control: EMMs: A 32.071; B 29.859; C 25.388; D 26.094 |
| Pavlidis 2026 | fPap morphology vs EGM thresholds () | All serial measurements | Selenium versus Control | Kendall tau-b | Selenium: Kendall tau-b 0.495 right apex and 0.483 left apex for fPap morphology and EGM thresholds; Control: Not a control-arm comparison |

**Panel A2. Extracted effects and planned analysis role**

| **Study** | **Outcome** | **Extracted effect** | **SE or CI** | **Adjustment status** | **Model or covariates** | **Preferred effect for analysis** | **Extraction confidence** |
| --- | --- | --- | --- | --- | --- | --- | --- |
| Yim 2026 | Grade 1 or more CIPN/paresthesia as reported (Grade >=1) | Not separately reported; raw arm data were retained when a documented calculation was possible. | Not reported | no | Not applicable/not reported | RR; Not selected for quantitative synthesis | moderate |
| Yim 2026 | Grade 2 or more motor dysfunction (Grade >=2) | Not separately reported; raw arm data were retained when a documented calculation was possible. | Not reported | no | Not applicable/not reported | RR; Exploratory quantitative synthesis | high |
| Yim 2026 | Grade 2 or more motor dysfunction (Grade >=2) | Not separately reported; raw arm data were retained when a documented calculation was possible. | Not reported | no | Not applicable/not reported | RR; Exploratory quantitative synthesis | high |
| Yim 2026 | Any grade adverse event (Any grade) | Not separately reported; raw arm data were retained when a documented calculation was possible. | Not reported | no | Not applicable/not reported | RR; Not selected for quantitative synthesis | high |
| Yim 2026 | CIPN20 sensory score (Continuous score) | Not separately reported; raw arm data were retained when a documented calculation was possible. | Not reported | no | Not applicable/not reported | MD when arm-level mean/SD are compatible; Not selected for quantitative synthesis | high |
| Yang 2023 | Myelosuppression (Grade 3) | Not separately reported; raw arm data were retained when a documented calculation was possible. | Not reported | no | Not applicable/not reported | RR; Exploratory quantitative synthesis | high |
| Yang 2023 | Myelosuppression (Grade 4) | Not separately reported; raw arm data were retained when a documented calculation was possible. | Not reported | no | Not applicable/not reported | RR; Exploratory quantitative synthesis | high |
| Yang 2023 | Diarrhea (Any diarrhea as table row) | Not separately reported; raw arm data were retained when a documented calculation was possible. | Not reported | no | Not applicable/not reported | RR; Exploratory quantitative synthesis | high |
| Yang 2023 | Whole blood selenium level (continuous) | Not separately reported; raw arm data were retained when a documented calculation was possible. | Not reported | no | Not applicable/not reported | MD when arm-level mean/SD are compatible; Not selected for quantitative synthesis | high |
| Laali 2020 | Cumulative OM incidence (Grade 1-4) | Not separately reported; raw arm data were retained when a documented calculation was possible. | Not reported | no | Not applicable/not reported | RR; Exploratory quantitative synthesis | high |
| Laali 2020 | Severe OM (Grade 3-4) | Not separately reported; raw arm data were retained when a documented calculation was possible. | Not reported | no | Not applicable/not reported | RR; Primary quantitative synthesis | high |
| Laali 2020 | Duration of OM (Grade 1-4 duration) | Not separately reported; raw arm data were retained when a documented calculation was possible. | Not reported | no | Not applicable/not reported | MD when arm-level mean/SD are compatible; Exploratory quantitative synthesis | high |
| Laali 2020 | Oral mucositis onset time (Continuous weeks; higher is favorable) | Not separately reported; raw arm data were retained when a documented calculation was possible. | Not reported | no | Not applicable/not reported | MD when arm-level mean/SD are compatible; Exploratory quantitative synthesis | high |
| Laali 2020 | Grade 3 neutropenia (Grade 3) | Not separately reported; raw arm data were retained when a documented calculation was possible. | Not reported | no | Not applicable/not reported | RR; Exploratory quantitative synthesis | high |
| Mix 2015 | Severe mucositis (Grade 3-4) | Not separately reported; raw arm data were retained when a documented calculation was possible. | Not reported | no | Not applicable/not reported | RR; Primary quantitative synthesis | high |
| Mix 2015 | Leukopenia grade >=2 (Grade >=2) | Not separately reported; raw arm data were retained when a documented calculation was possible. | Not reported | no | Not applicable/not reported | RR; Exploratory quantitative synthesis | high |
| Vieira 2015 | Solid tumor nausea score (Continuous score; median and quartiles) | Not separately reported; raw arm data were retained when a documented calculation was possible. | Not reported | no | Not applicable/not reported | No conversion; narrative or single-study display; Narrative or single-study display | moderate |
| Vieira 2015 | AST all patients (Continuous laboratory value) | Not separately reported; raw arm data were retained when a documented calculation was possible. | Not reported | no | Not applicable/not reported | No conversion; narrative or single-study display; Narrative or single-study display | moderate |
| Jahangard-Rafsanjani 2013 | Any oral mucositis (WHO grade 1-4) | Not separately reported; raw arm data were retained when a documented calculation was possible. | Not reported | no | Not applicable/not reported | RR; Exploratory quantitative synthesis | high |
| Jahangard-Rafsanjani 2013 | Severe oral mucositis (WHO grade 3-4) | Not separately reported; raw arm data were retained when a documented calculation was possible. | Not reported | no | Not applicable/not reported | RR; Exploratory quantitative synthesis | high |
| Jahangard-Rafsanjani 2013 | Duration of grade 2-4 oral mucositis (WHO grade 2-4 duration) | Not separately reported; raw arm data were retained when a documented calculation was possible. | Not reported | no | Not applicable/not reported | MD when arm-level mean/SD are compatible; Exploratory quantitative synthesis | high |
| Jahangard-Rafsanjani 2013 | Serum creatinine elevation (Trial-defined elevation) | Not separately reported; raw arm data were retained when a documented calculation was possible. | Not reported | no | Not applicable/not reported | RR; Exploratory quantitative synthesis | high |
| Ghorbani 2013 | Acute kidney injury (Trial-defined AKI) | Not separately reported; raw arm data were retained when a documented calculation was possible. | Not reported | no | Not applicable/not reported | RR; Exploratory quantitative synthesis | high |
| Muecke 2010 | CTC grade 1-3 diarrhea (CTC grade 1-3) | Not separately reported; raw arm data were retained when a documented calculation was possible. | Not reported | no | Not applicable/not reported | RR; Exploratory quantitative synthesis | high |
| Muecke 2010 | CTC grade 2 or higher diarrhea (CTC grade >=2) | Not separately reported; raw arm data were retained when a documented calculation was possible. | Not reported | no | Not applicable/not reported | RR; Not selected for quantitative synthesis | high |
| Buentzel 2010 | Maximal stomatitis (RTOG grade >=3) | Not separately reported; raw arm data were retained when a documented calculation was possible. | Not reported | no | Not applicable/not reported | RR; Primary quantitative synthesis | high |
| Buentzel 2010 | Maximal dysphagia (RTOG grade >=3) | Not separately reported; raw arm data were retained when a documented calculation was possible. | Not reported | no | Not applicable/not reported | RR; Not selected for quantitative synthesis | high |
| Asfour 2006 | Infection rate (Infection yes/no) | Not separately reported; raw arm data were retained when a documented calculation was possible. | Not reported | no | Not applicable/not reported | RR; Exploratory quantitative synthesis | moderate |
| Asfour 2006 | Cardiac ejection fraction (Continuous EF %) | Not separately reported; raw arm data were retained when a documented calculation was possible. | Not reported | no | Not applicable/not reported | MD when arm-level mean/SD are compatible; Not selected for quantitative synthesis | moderate |
| Hu 1997 | Peripheral WBC count (Continuous WBC x10^9/L) | Not separately reported; raw arm data were retained when a documented calculation was possible. | Not reported | no | Not applicable/not reported | MD when arm-level mean/SD are compatible; Narrative or single-study display | moderate |
| Hu 1997 | GCSF consumption (Continuous IU) | Not separately reported; raw arm data were retained when a documented calculation was possible. | Not reported | no | Not applicable/not reported | MD when arm-level mean/SD are compatible; Narrative or single-study display | moderate |
| Hu 1997 | Urine NAG (Continuous urine enzyme units/L) | Not separately reported; raw arm data were retained when a documented calculation was possible. | Not reported | no | Not applicable/not reported | MD when arm-level mean/SD are compatible; Narrative or single-study display | moderate |
| Hu 1997 | Urine GGT (Continuous urine enzyme units/L) | Not separately reported; raw arm data were retained when a documented calculation was possible. | Not reported | no | Not applicable/not reported | MD when arm-level mean/SD are compatible; Narrative or single-study display | moderate |
| Ameri 2016 | Mean mucositis score (Continuous mucositis score) | Not separately reported; raw arm data were retained when a documented calculation was possible. | Not reported | no | Not applicable/not reported | MD when arm-level mean/SD are compatible; Narrative or single-study display | moderate |
| Ameri 2016 | Mean mucositis score (Continuous mucositis score) | Not separately reported; raw arm data were retained when a documented calculation was possible. | Not reported | no | Not applicable/not reported | MD when arm-level mean/SD are compatible; Narrative or single-study display | moderate |
| Ameri 2016 | Mean mucositis score (Continuous mucositis score) | Not separately reported; raw arm data were retained when a documented calculation was possible. | Not reported | no | Not applicable/not reported | MD when arm-level mean/SD are compatible; Narrative or single-study display | moderate |
| Chen 2023 | Any adverse reaction grade I-IV (Any grade I-IV) | Not separately reported; raw arm data were retained when a documented calculation was possible. | Not reported | no | Not applicable/not reported | RR; Exploratory quantitative synthesis | moderate |
| Chen 2023 | Grade III-IV adverse reactions (Grade III-IV) | Not separately reported; raw arm data were retained when a documented calculation was possible. | Not reported | no | Not applicable/not reported | RR; Exploratory quantitative synthesis | moderate |
| Chen 2023 | Gastrointestinal reactions (Any grade) | Not separately reported; raw arm data were retained when a documented calculation was possible. | Not reported | no | Not applicable/not reported | RR; Exploratory quantitative synthesis | low |
| Chen 2023 | Bone marrow suppression (Any grade) | Not separately reported; raw arm data were retained when a documented calculation was possible. | Not reported | no | Not applicable/not reported | RR; Exploratory quantitative synthesis | low |
| Chen 2023 | Pain grade >=2 (Grade >=2) | Not separately reported; raw arm data were retained when a documented calculation was possible. | Not reported | no | Not applicable/not reported | RR; Exploratory quantitative synthesis | moderate |
| Wang 2020 | Severe oral mucositis (Grade >=3) | Not separately reported; raw arm data were retained when a documented calculation was possible. | Not reported | no | Not applicable/not reported | RR; Primary quantitative synthesis | high |
| Wang 2020 | Oral mucositis onset time (Continuous days; higher is favorable) | Not separately reported; raw arm data were retained when a documented calculation was possible. | Not reported | no | Not applicable/not reported | MD when arm-level mean/SD are compatible; Exploratory quantitative synthesis | high |
| Wang 2020 | Pain grade >=2 (Grade >=2) | Not separately reported; raw arm data were retained when a documented calculation was possible. | Not reported | no | Not applicable/not reported | RR; Exploratory quantitative synthesis | high |
| Wang 2020 | Pain relief time (Continuous days; lower is favorable) | Not separately reported; raw arm data were retained when a documented calculation was possible. | Not reported | no | Not applicable/not reported | MD when arm-level mean/SD are compatible; Not selected for quantitative synthesis | high |
| Son 2017 | Serum amylase 2 days after I-131 (Higher is worse) | Not separately reported; raw arm data were retained when a documented calculation was possible. | Not reported | no | Not applicable/not reported | No conversion; narrative or single-study display; Narrative or single-study display | high |
| Son 2017 | Questionnaire score 6 months after I-131 (Higher is worse) | Not separately reported; raw arm data were retained when a documented calculation was possible. | Not reported | no | Not applicable/not reported | No conversion; narrative or single-study display; Narrative or single-study display | high |
| Son 2017 | Salivary gland scintigraphy parameters () | Not separately reported; raw arm data were retained when a documented calculation was possible. | Not reported | no | Model details as reported; no new calculation | No conversion; narrative or single-study display; Narrative or single-study display | high |
| Bahador 2022 | Diarrhea grade II/III vs grade 0 (Grade II/III to grade 0) | GEE OR 3.6 | Not reported | yes | GEE model parameters not fully specified | OR retained separately; OR-only display; not combined with RR | moderate |
| Bahador 2022 | Diarrhea grade II/III vs grade I (Grade II/III to grade I) | GEE OR 2.3 | Not reported | yes | GEE model parameters not fully specified | OR retained separately; OR-only display; not combined with RR | moderate |
| Bahador 2022 | Diarrhea grade I vs grade 0 (Grade I to grade 0) | GEE OR 1.7 | Not reported | yes | GEE model parameters not fully specified | OR retained separately; OR-only display; not combined with RR | moderate |
| Bahador 2022 | Pain (Not clearly specified) | GEE OR 0.56 | Not reported | yes | GEE model parameters not fully specified | OR retained separately; OR-only display; not combined with RR | low |
| Pavlidis 2026 | Electrogustometry thresholds (Continuous dB; lower is favorable) | Not separately reported; raw arm data were retained when a documented calculation was possible. | Not reported | yes | Linear mixed model as reported | No conversion; narrative or single-study display; Narrative or single-study display | high |
| Pavlidis 2026 | fPap morphology vs EGM thresholds () | Not separately reported; raw arm data were retained when a documented calculation was possible. | Not reported | yes | Model details as reported; no new calculation | No conversion; narrative or single-study display; Narrative or single-study display | moderate |

**Panel B1. Processing and direction harmonization**

| **Study** | **Outcome** | **Original metric** | **Processing step** | **Formula or rule** | **Converted metric** | **Direction harmonization** |
| --- | --- | --- | --- | --- | --- | --- |
| Yim 2026 | Grade 1 or more CIPN/paresthesia as reported | Event count | Compute study RR from harmonized selenium/control 2x2 counts on the log scale; add 0.5 to all four cells only when any cell is zero.; none | RR=(a/n_selenium)/(c/n_control); inverse-variance analysis on log(RR) | RR | For harmful toxicity outcomes, lower event risk favors selenium after aligning to selenium vs control. |
| Yim 2026 | Grade 2 or more motor dysfunction | Event count | Compute study RR from harmonized selenium/control 2x2 counts on the log scale; add 0.5 to all four cells only when any cell is zero.; none | RR=(a/n_selenium)/(c/n_control); inverse-variance analysis on log(RR) | RR | For harmful toxicity outcomes, lower event risk favors selenium after aligning to selenium vs control. |
| Yim 2026 | Grade 2 or more motor dysfunction | Event count | Compute study RR from harmonized selenium/control 2x2 counts on the log scale; add 0.5 to all four cells only when any cell is zero.; none | RR=(a/n_selenium)/(c/n_control); inverse-variance analysis on log(RR) | RR | For harmful toxicity outcomes, lower event risk favors selenium after aligning to selenium vs control. |
| Yim 2026 | Any grade adverse event | Event count | Compute study RR from harmonized selenium/control 2x2 counts on the log scale; add 0.5 to all four cells only when any cell is zero.; none | RR=(a/n_selenium)/(c/n_control); inverse-variance analysis on log(RR) | RR | For harmful toxicity outcomes, lower event risk favors selenium after aligning to selenium vs control. |
| Yim 2026 | CIPN20 sensory score | Continuous mean and SD | Compute MD as selenium mean minus control mean; SE from arm SDs and denominators when compatible.; MD calculated only for single-study display | MD=mean_selenium-mean_control; SE=sqrt(SD_selenium^2/n_selenium+SD_control^2/n_control) | MD when arm-level mean/SD are compatible | For harmful symptom scores, a lower mean after alignment favors selenium. |
| Yang 2023 | Myelosuppression | Event count | Compute study RR from harmonized selenium/control 2x2 counts on the log scale; add 0.5 to all four cells only when any cell is zero.; none | RR=(a/n_selenium)/(c/n_control); inverse-variance analysis on log(RR) | RR | For harmful toxicity outcomes, lower event risk favors selenium after aligning to selenium vs control. |
| Yang 2023 | Myelosuppression | Event count | Compute study RR from harmonized selenium/control 2x2 counts on the log scale; add 0.5 to all four cells only when any cell is zero.; none | RR=(a/n_selenium)/(c/n_control); inverse-variance analysis on log(RR) | RR | For harmful toxicity outcomes, lower event risk favors selenium after aligning to selenium vs control. |
| Yang 2023 | Diarrhea | Event count | Compute study RR from harmonized selenium/control 2x2 counts on the log scale; add 0.5 to all four cells only when any cell is zero.; none | RR=(a/n_selenium)/(c/n_control); inverse-variance analysis on log(RR) | RR | For harmful toxicity outcomes, lower event risk favors selenium after aligning to selenium vs control. |
| Yang 2023 | Whole blood selenium level | Mean and SD | Compute MD as selenium mean minus control mean; SE from arm SDs and denominators when compatible.; none | MD=mean_selenium-mean_control; SE=sqrt(SD_selenium^2/n_selenium+SD_control^2/n_control) | MD when arm-level mean/SD are compatible | For continuous toxicity scores/biomarkers, direction depends on outcome definition; preserve original before any MD/SMD. |
| Laali 2020 | Cumulative OM incidence | Event count | Compute study RR from harmonized selenium/control 2x2 counts on the log scale; add 0.5 to all four cells only when any cell is zero.; none | RR=(a/n_selenium)/(c/n_control); inverse-variance analysis on log(RR) | RR | For harmful toxicity outcomes, lower event risk favors selenium after aligning to selenium vs control. |
| Laali 2020 | Severe OM | Event count | Compute study RR from harmonized selenium/control 2x2 counts on the log scale; add 0.5 to all four cells only when any cell is zero.; none | RR=(a/n_selenium)/(c/n_control); inverse-variance analysis on log(RR) | RR | For harmful toxicity outcomes, lower event risk favors selenium after aligning to selenium vs control. |
| Laali 2020 | Duration of OM | Mean and SD | Compute MD as selenium mean minus control mean; SE from arm SDs and denominators when compatible.; none | MD=mean_selenium-mean_control; SE=sqrt(SD_selenium^2/n_selenium+SD_control^2/n_control) | MD when arm-level mean/SD are compatible | For continuous toxicity scores/biomarkers, direction depends on outcome definition; preserve original before any MD/SMD. |
| Laali 2020 | Oral mucositis onset time | Mean and SD | Compute MD as selenium mean minus control mean; SE from arm SDs and denominators when compatible.; Both arm means and SDs multiplied by 7 before MD calculation | MD=mean_selenium-mean_control; SE=sqrt(SD_selenium^2/n_selenium+SD_control^2/n_control) | MD when arm-level mean/SD are compatible | For oral-mucositis onset, a positive MD after alignment indicates later onset and favors selenium. |
| Laali 2020 | Grade 3 neutropenia | Event count | Compute study RR from harmonized selenium/control 2x2 counts on the log scale; add 0.5 to all four cells only when any cell is zero.; none | RR=(a/n_selenium)/(c/n_control); inverse-variance analysis on log(RR) | RR | For harmful toxicity outcomes, lower event risk favors selenium after aligning to selenium vs control. |
| Mix 2015 | Severe mucositis | Event count | Compute study RR from harmonized selenium/control 2x2 counts on the log scale; add 0.5 to all four cells only when any cell is zero.; none | RR=(a/n_selenium)/(c/n_control); inverse-variance analysis on log(RR) | RR | For harmful toxicity outcomes, lower event risk favors selenium after aligning to selenium vs control. |
| Mix 2015 | Leukopenia grade >=2 | Event count | Compute study RR from harmonized selenium/control 2x2 counts on the log scale; add 0.5 to all four cells only when any cell is zero.; none | RR=(a/n_selenium)/(c/n_control); inverse-variance analysis on log(RR) | RR | For harmful toxicity outcomes, lower event risk favors selenium after aligning to selenium vs control. |
| Vieira 2015 | Solid tumor nausea score | Median and IQR | No distributional conversion was applied; the reported summary was retained for narrative or single-study display.; none | Not applicable | No conversion; narrative or single-study display | For continuous toxicity scores/biomarkers, direction depends on outcome definition; preserve original before any MD/SMD. |
| Vieira 2015 | AST all patients | Median and range | No distributional conversion was applied; the reported summary was retained for narrative or single-study display.; none | Not applicable | No conversion; narrative or single-study display | For continuous toxicity scores/biomarkers, direction depends on outcome definition; preserve original before any MD/SMD. |
| Jahangard-Rafsanjani 2013 | Any oral mucositis | Event count | Compute study RR from harmonized selenium/control 2x2 counts on the log scale; add 0.5 to all four cells only when any cell is zero.; none | RR=(a/n_selenium)/(c/n_control); inverse-variance analysis on log(RR) | RR | For harmful toxicity outcomes, lower event risk favors selenium after aligning to selenium vs control. |
| Jahangard-Rafsanjani 2013 | Severe oral mucositis | Event count | Compute study RR from harmonized selenium/control 2x2 counts on the log scale; add 0.5 to all four cells only when any cell is zero.; none | RR=(a/n_selenium)/(c/n_control); inverse-variance analysis on log(RR) | RR | For harmful toxicity outcomes, lower event risk favors selenium after aligning to selenium vs control. |
| Jahangard-Rafsanjani 2013 | Duration of grade 2-4 oral mucositis | Mean and SD | Compute MD as selenium mean minus control mean; SE from arm SDs and denominators when compatible.; none | MD=mean_selenium-mean_control; SE=sqrt(SD_selenium^2/n_selenium+SD_control^2/n_control) | MD when arm-level mean/SD are compatible | For continuous toxicity scores/biomarkers, direction depends on outcome definition; preserve original before any MD/SMD. |
| Jahangard-Rafsanjani 2013 | Serum creatinine elevation | Event count | Compute study RR from harmonized selenium/control 2x2 counts on the log scale; add 0.5 to all four cells only when any cell is zero.; none | RR=(a/n_selenium)/(c/n_control); inverse-variance analysis on log(RR) | RR | For harmful toxicity outcomes, lower event risk favors selenium after aligning to selenium vs control. |
| Ghorbani 2013 | Acute kidney injury | Event count | Compute study RR from harmonized selenium/control 2x2 counts on the log scale; add 0.5 to all four cells only when any cell is zero.; none | RR=(a/n_selenium)/(c/n_control); inverse-variance analysis on log(RR) | RR | For harmful toxicity outcomes, lower event risk favors selenium after aligning to selenium vs control. |
| Muecke 2010 | CTC grade 1-3 diarrhea | Event count | Compute study RR from harmonized selenium/control 2x2 counts on the log scale; add 0.5 to all four cells only when any cell is zero.; none | RR=(a/n_selenium)/(c/n_control); inverse-variance analysis on log(RR) | RR | For harmful toxicity outcomes, lower event risk favors selenium after aligning to selenium vs control. |
| Muecke 2010 | CTC grade 2 or higher diarrhea | Reported percentage | No distributional conversion was applied; the reported summary was retained for narrative or single-study display.; none | RR=(a/n_selenium)/(c/n_control); inverse-variance analysis on log(RR) | RR | For harmful toxicity outcomes, lower event risk favors selenium after aligning to selenium vs control. |
| Buentzel 2010 | Maximal stomatitis | Event count | Compute study RR from harmonized selenium/control 2x2 counts on the log scale; add 0.5 to all four cells only when any cell is zero.; none | RR=(a/n_selenium)/(c/n_control); inverse-variance analysis on log(RR) | RR | For harmful toxicity outcomes, lower event risk favors selenium after aligning to selenium vs control. |
| Buentzel 2010 | Maximal dysphagia | Event count | Compute study RR from harmonized selenium/control 2x2 counts on the log scale; add 0.5 to all four cells only when any cell is zero.; none | RR=(a/n_selenium)/(c/n_control); inverse-variance analysis on log(RR) | RR | For harmful toxicity outcomes, lower event risk favors selenium after aligning to selenium vs control. |
| Asfour 2006 | Infection rate | Event count inferred from reported percentages | Compute study RR from harmonized selenium/control 2x2 counts on the log scale; add 0.5 to all four cells only when any cell is zero.; Events were inferred from reported percentages and n=15 per group; the result was retained only in the broad exploratory synthesis | RR=(a/n_selenium)/(c/n_control); inverse-variance analysis on log(RR) | RR | For harmful toxicity outcomes, lower event risk favors selenium after aligning to selenium vs control. |
| Asfour 2006 | Cardiac ejection fraction | Mean and SD within group | Compute MD as selenium mean minus control mean; SE from arm SDs and denominators when compatible.; none | MD=mean_selenium-mean_control; SE=sqrt(SD_selenium^2/n_selenium+SD_control^2/n_control) | MD when arm-level mean/SD are compatible | For continuous toxicity scores/biomarkers, direction depends on outcome definition; preserve original before any MD/SMD. |
| Hu 1997 | Peripheral WBC count | Mean and SD | Compute MD as selenium mean minus control mean; SE from arm SDs and denominators when compatible.; none | MD=mean_selenium-mean_control; SE=sqrt(SD_selenium^2/n_selenium+SD_control^2/n_control) | MD when arm-level mean/SD are compatible | For continuous toxicity scores/biomarkers, direction depends on outcome definition; preserve original before any MD/SMD. |
| Hu 1997 | GCSF consumption | Mean and SD | Compute MD as selenium mean minus control mean; SE from arm SDs and denominators when compatible.; none | MD=mean_selenium-mean_control; SE=sqrt(SD_selenium^2/n_selenium+SD_control^2/n_control) | MD when arm-level mean/SD are compatible | For continuous toxicity scores/biomarkers, direction depends on outcome definition; preserve original before any MD/SMD. |
| Hu 1997 | Urine NAG | Mean and SD | Compute MD as selenium mean minus control mean; SE from arm SDs and denominators when compatible.; none | MD=mean_selenium-mean_control; SE=sqrt(SD_selenium^2/n_selenium+SD_control^2/n_control) | MD when arm-level mean/SD are compatible | For continuous toxicity scores/biomarkers, direction depends on outcome definition; preserve original before any MD/SMD. |
| Hu 1997 | Urine GGT | Mean and SD | Compute MD as selenium mean minus control mean; SE from arm SDs and denominators when compatible.; none | MD=mean_selenium-mean_control; SE=sqrt(SD_selenium^2/n_selenium+SD_control^2/n_control) | MD when arm-level mean/SD are compatible | For continuous toxicity scores/biomarkers, direction depends on outcome definition; preserve original before any MD/SMD. |
| Ameri 2016 | Mean mucositis score | Mean and SD | Compute MD as selenium mean minus control mean; SE from arm SDs and denominators when compatible.; MD calculated for single-study display using verified n=15 per arm | MD=mean_selenium-mean_control; SE=sqrt(SD_selenium^2/n_selenium+SD_control^2/n_control) | MD when arm-level mean/SD are compatible | For continuous toxicity scores/biomarkers, direction depends on outcome definition; preserve original before any MD/SMD. |
| Ameri 2016 | Mean mucositis score | Mean and SD | Compute MD as selenium mean minus control mean; SE from arm SDs and denominators when compatible.; MD calculated for single-study display using verified n=15 per arm | MD=mean_selenium-mean_control; SE=sqrt(SD_selenium^2/n_selenium+SD_control^2/n_control) | MD when arm-level mean/SD are compatible | For continuous toxicity scores/biomarkers, direction depends on outcome definition; preserve original before any MD/SMD. |
| Ameri 2016 | Mean mucositis score | Mean and SD | Compute MD as selenium mean minus control mean; SE from arm SDs and denominators when compatible.; MD calculated for single-study display using verified n=15 per arm | MD=mean_selenium-mean_control; SE=sqrt(SD_selenium^2/n_selenium+SD_control^2/n_control) | MD when arm-level mean/SD are compatible | For continuous toxicity scores/biomarkers, direction depends on outcome definition; preserve original before any MD/SMD. |
| Chen 2023 | Any adverse reaction grade I-IV | Event count; None reported | Compute study RR from harmonized selenium/control 2x2 counts on the log scale; add 0.5 to all four cells only when any cell is zero.; No new effect size calculated; incidence from article text/table | RR=(a/n_selenium)/(c/n_control); inverse-variance analysis on log(RR) | RR | For harmful toxicity events, lower RR/event proportion favors selenium when group 1 is selenium. |
| Chen 2023 | Grade III-IV adverse reactions | Event count; None reported | Compute study RR from harmonized selenium/control 2x2 counts on the log scale; add 0.5 to all four cells only when any cell is zero.; Grade III + grade IV counts from Table V | RR=(a/n_selenium)/(c/n_control); inverse-variance analysis on log(RR) | RR | For harmful toxicity events, lower RR/event proportion favors selenium when group 1 is selenium. |
| Chen 2023 | Gastrointestinal reactions | Event count; None reported | Compute study RR from harmonized selenium/control 2x2 counts on the log scale; add 0.5 to all four cells only when any cell is zero.; Summed cisplatin/carboplatin/oxaliplatin/nedaplatin GI reaction counts in Table IV | RR=(a/n_selenium)/(c/n_control); inverse-variance analysis on log(RR) | RR | For harmful toxicity events, lower RR/event proportion favors selenium when group 1 is selenium. |
| Chen 2023 | Bone marrow suppression | Event count; None reported | Compute study RR from harmonized selenium/control 2x2 counts on the log scale; add 0.5 to all four cells only when any cell is zero.; Summed platinum subgroup bone marrow suppression counts in Table IV | RR=(a/n_selenium)/(c/n_control); inverse-variance analysis on log(RR) | RR | For harmful toxicity events, lower RR/event proportion favors selenium when group 1 is selenium. |
| Chen 2023 | Pain grade >=2 | Event count; None reported | Compute study RR from harmonized selenium/control 2x2 counts on the log scale; add 0.5 to all four cells only when any cell is zero.; Grade II + grade III counts from Table VI | RR=(a/n_selenium)/(c/n_control); inverse-variance analysis on log(RR) | RR | For harmful toxicity events, lower RR/event proportion favors selenium when group 1 is selenium. |
| Wang 2020 | Severe oral mucositis | Event count; None reported | Compute study RR from harmonized selenium/control 2x2 counts on the log scale; add 0.5 to all four cells only when any cell is zero.; No new effect size calculated; grade >=3 events reported in Table 1 | RR=(a/n_selenium)/(c/n_control); inverse-variance analysis on log(RR) | RR | For harmful toxicity events, lower RR/event proportion favors selenium when group 1 is selenium. |
| Wang 2020 | Oral mucositis onset time | Continuous mean and SD; None reported | Compute MD as selenium mean minus control mean; SE from arm SDs and denominators when compatible.; No new effect size calculated | MD=mean_selenium-mean_control; SE=sqrt(SD_selenium^2/n_selenium+SD_control^2/n_control) | MD when arm-level mean/SD are compatible | Higher mean indicates later onset and favors selenium; do not interpret as harmful-event direction. |
| Wang 2020 | Pain grade >=2 | Event count; None reported | Compute study RR from harmonized selenium/control 2x2 counts on the log scale; add 0.5 to all four cells only when any cell is zero.; No new effect size calculated; grade >=2 events reported in Table 2 | RR=(a/n_selenium)/(c/n_control); inverse-variance analysis on log(RR) | RR | For harmful toxicity events, lower RR/event proportion favors selenium when group 1 is selenium. |
| Wang 2020 | Pain relief time | Continuous mean and SD; None reported | Compute MD as selenium mean minus control mean; SE from arm SDs and denominators when compatible.; No new effect size calculated | MD=mean_selenium-mean_control; SE=sqrt(SD_selenium^2/n_selenium+SD_control^2/n_control) | MD when arm-level mean/SD are compatible | Lower mean indicates shorter pain relief time and favors selenium. |
| Son 2017 | Serum amylase 2 days after I-131 | Median and range; None reported | No distributional conversion was applied; the reported summary was retained for narrative or single-study display.; No new effect size calculated | Not applicable | No conversion; narrative or single-study display | Narrative/model/correlation result only; no harmonized pooled effect. |
| Son 2017 | Questionnaire score 6 months after I-131 | Median and range; None reported | No distributional conversion was applied; the reported summary was retained for narrative or single-study display.; No new effect size calculated | Not applicable | No conversion; narrative or single-study display | Narrative/model/correlation result only; no harmonized pooled effect. |
| Son 2017 | Salivary gland scintigraphy parameters | Median/range table; Within-arm Wilcoxon comparisons; no direct between-arm effect size | No distributional conversion was applied; the reported summary was retained for narrative or single-study display.; No new effect size calculated | Not applicable | No conversion; narrative or single-study display | Narrative/model/correlation result only; no harmonized pooled effect. |
| Bahador 2022 | Diarrhea grade II/III vs grade 0 | Reported OR; GEE OR | Retain the reported adjusted OR and CI without conversion to RR.; No new effect size calculated | Not applicable | OR retained separately | The reported OR direction was inferred as control/no selenium versus selenium; OR >1 therefore favors selenium for harmful toxicity, but the direction remains uncertain. |
| Bahador 2022 | Diarrhea grade II/III vs grade I | Reported OR; GEE OR | Retain the reported adjusted OR and CI without conversion to RR.; No new effect size calculated | Not applicable | OR retained separately | The reported OR direction was inferred as control/no selenium versus selenium; OR >1 therefore favors selenium for harmful toxicity, but the direction remains uncertain. |
| Bahador 2022 | Diarrhea grade I vs grade 0 | Reported OR; GEE OR | Retain the reported adjusted OR and CI without conversion to RR.; No new effect size calculated | Not applicable | OR retained separately | The reported OR direction was inferred as control/no selenium versus selenium; OR >1 therefore favors selenium for harmful toxicity, but the direction remains uncertain. |
| Bahador 2022 | Pain | Reported OR; GEE OR | Retain the reported adjusted OR and CI without conversion to RR.; No new effect size calculated | Not applicable | OR retained separately | The reported OR direction was inferred as control/no selenium versus selenium; OR >1 therefore favors selenium for harmful toxicity, but the direction remains uncertain. |
| Pavlidis 2026 | Electrogustometry thresholds | Reported model result; LMM EMMs and Sidak-adjusted pairwise comparisons | No distributional conversion was applied; the reported summary was retained for narrative or single-study display.; Control-minus-selenium adjusted differences: A 1.365; B 0.918; C 0.541; D 0.376; E 0.776; F 1.482 | Not applicable | No conversion; narrative or single-study display | Narrative/model/correlation result only; no harmonized pooled effect. |
| Pavlidis 2026 | fPap morphology vs EGM thresholds | correlation; Kendall tau-b | No distributional conversion was applied; the reported summary was retained for narrative or single-study display.; No new effect size calculated | Not applicable | No conversion; narrative or single-study display | Narrative/model/correlation result only; no harmonized pooled effect. |

**Panel B2. Assumptions, sensitivity requirements, and verification**

| **Study** | **Outcome** | **Reference group handling** | **Assumption** | **Sensitivity requirement** | **Verification status** |
| --- | --- | --- | --- | --- | --- |
| Yim 2026 | Grade 1 or more CIPN/paresthesia as reported | Original: Selenium vs placebo; target: Selenium supplementation vs control; reversed: no. | Independent comparison groups; original outcome definition, threshold, and time point retained; no OR/RR or incompatible outcome mixing. | yes | Verified against the study report; comparison direction and synthesis role checked |
| Yim 2026 | Grade 2 or more motor dysfunction | Original: Placebo vs selenium in table; target: Selenium supplementation vs control; reversal=yes. | Independent comparison groups; original outcome definition, threshold, and time point retained; no OR/RR or incompatible outcome mixing. | no | Verified against the study report; comparison direction and synthesis role checked |
| Yim 2026 | Grade 2 or more motor dysfunction | Original: Placebo vs selenium in table; target: Selenium supplementation vs control; reversal=yes. | Independent comparison groups; original outcome definition, threshold, and time point retained; no OR/RR or incompatible outcome mixing. | no | Verified against the study report; comparison direction and synthesis role checked |
| Yim 2026 | Any grade adverse event | Original: Placebo vs selenium in table; target: Selenium supplementation vs control; reversal=yes. | Independent comparison groups; original outcome definition, threshold, and time point retained; no OR/RR or incompatible outcome mixing. | no | Verified against the study report; comparison direction and synthesis role checked |
| Yim 2026 | CIPN20 sensory score | Original: Selenium vs placebo; target: Selenium supplementation vs control; reversed: no. | Independent comparison groups; original outcome definition, threshold, and time point retained; no OR/RR or incompatible outcome mixing. | no | Verified against the study report; comparison direction and synthesis role checked |
| Yang 2023 | Myelosuppression | Original: Selenium vs placebo; target: Selenium supplementation vs control; reversed: no. | Independent comparison groups; original outcome definition, threshold, and time point retained; no OR/RR or incompatible outcome mixing. | no | Verified against the study report; comparison direction and synthesis role checked |
| Yang 2023 | Myelosuppression | Original: Selenium vs placebo; target: Selenium supplementation vs control; reversed: no. | Independent comparison groups; original outcome definition, threshold, and time point retained; no OR/RR or incompatible outcome mixing. | no | Verified against the study report; comparison direction and synthesis role checked |
| Yang 2023 | Diarrhea | Original: Selenium vs placebo; target: Selenium supplementation vs control; reversed: no. | Independent comparison groups; original outcome definition, threshold, and time point retained; no OR/RR or incompatible outcome mixing. | no | Verified against the study report; comparison direction and synthesis role checked |
| Yang 2023 | Whole blood selenium level | Original: Selenium vs placebo; target: Selenium supplementation vs control; reversed: no. | Independent comparison groups; original outcome definition, threshold, and time point retained; no OR/RR or incompatible outcome mixing. | no | Verified against the study report; comparison direction and synthesis role checked |
| Laali 2020 | Cumulative OM incidence | Original: Selenium vs placebo; target: Selenium supplementation vs control; reversed: no. | Independent comparison groups; original outcome definition, threshold, and time point retained; no OR/RR or incompatible outcome mixing. | no | Verified against the study report; comparison direction and synthesis role checked |
| Laali 2020 | Severe OM | Original: Selenium vs placebo; target: Selenium supplementation vs control; reversed: no. | Independent comparison groups; original outcome definition, threshold, and time point retained; no OR/RR or incompatible outcome mixing. | no | Verified against the study report; comparison direction and synthesis role checked |
| Laali 2020 | Duration of OM | Original: Selenium vs placebo; target: Selenium supplementation vs control; reversed: no. | Independent comparison groups; original outcome definition, threshold, and time point retained; no OR/RR or incompatible outcome mixing. | no | Verified against the study report; comparison direction and synthesis role checked |
| Laali 2020 | Oral mucositis onset time | Original: Selenium vs placebo; target: Selenium supplementation vs control; reversed: no. | Independent comparison groups; original outcome definition, threshold, and time point retained; no OR/RR or incompatible outcome mixing. | no | Verified against the study report; comparison direction and synthesis role checked |
| Laali 2020 | Grade 3 neutropenia | Original: Selenium vs placebo; target: Selenium supplementation vs control; reversed: no. | Independent comparison groups; original outcome definition, threshold, and time point retained; no OR/RR or incompatible outcome mixing. | no | Verified against the study report; comparison direction and synthesis role checked |
| Mix 2015 | Severe mucositis | Original: Placebo vs SLM in table; target: Selenium supplementation vs control; reversal=yes. | Independent comparison groups; original outcome definition, threshold, and time point retained; no OR/RR or incompatible outcome mixing. | yes | Verified against the study report; comparison direction and synthesis role checked |
| Mix 2015 | Leukopenia grade >=2 | Original: Placebo vs SLM in table; target: Selenium supplementation vs control; reversal=yes. | Independent comparison groups; original outcome definition, threshold, and time point retained; no OR/RR or incompatible outcome mixing. | yes | Verified against the study report; comparison direction and synthesis role checked |
| Vieira 2015 | Solid tumor nausea score | Original: Crossover period comparison; target: Selenium supplementation vs control; reversed: no. | Independent comparison groups; original outcome definition, threshold, and time point retained; no OR/RR or incompatible outcome mixing. | yes | Verified against the study report; comparison direction and synthesis role checked |
| Vieira 2015 | AST all patients | Original: Crossover period comparison; target: Selenium supplementation vs control; reversed: no. | Independent comparison groups; original outcome definition, threshold, and time point retained; no OR/RR or incompatible outcome mixing. | yes | Verified against the study report; comparison direction and synthesis role checked |
| Jahangard-Rafsanjani 2013 | Any oral mucositis | Original: Selenium vs control; target: Selenium supplementation vs control; reversed: no. | Independent comparison groups; original outcome definition, threshold, and time point retained; no OR/RR or incompatible outcome mixing. | no | Verified against the study report; comparison direction and synthesis role checked |
| Jahangard-Rafsanjani 2013 | Severe oral mucositis | Original: Selenium vs control; target: Selenium supplementation vs control; reversed: no. | Independent comparison groups; original outcome definition, threshold, and time point retained; no OR/RR or incompatible outcome mixing. | no | Verified against the study report; comparison direction and synthesis role checked |
| Jahangard-Rafsanjani 2013 | Duration of grade 2-4 oral mucositis | Original: Selenium vs control; target: Selenium supplementation vs control; reversed: no. | Independent comparison groups; original outcome definition, threshold, and time point retained; no OR/RR or incompatible outcome mixing. | no | Verified against the study report; comparison direction and synthesis role checked |
| Jahangard-Rafsanjani 2013 | Serum creatinine elevation | Original: Selenium vs control; target: Selenium supplementation vs control; reversed: no. | Independent comparison groups; original outcome definition, threshold, and time point retained; no OR/RR or incompatible outcome mixing. | no | Verified against the study report; comparison direction and synthesis role checked |
| Ghorbani 2013 | Acute kidney injury | Original: Selenium vs placebo; target: Selenium supplementation vs control; reversed: no. | Independent comparison groups; original outcome definition, threshold, and time point retained; no OR/RR or incompatible outcome mixing. | no | Verified against the study report; comparison direction and synthesis role checked |
| Muecke 2010 | CTC grade 1-3 diarrhea | Original: Selenium vs no supplementation; target: Selenium supplementation vs control; reversed: no. | Independent comparison groups; original outcome definition, threshold, and time point retained; no OR/RR or incompatible outcome mixing. | yes | Verified against the study report; comparison direction and synthesis role checked |
| Muecke 2010 | CTC grade 2 or higher diarrhea | Original: Selenium vs no supplementation; target: Selenium supplementation vs control; reversed: no. | Independent comparison groups; original outcome definition, threshold, and time point retained; no OR/RR or incompatible outcome mixing. | yes | Verified against the study report; comparison direction and synthesis role checked |
| Buentzel 2010 | Maximal stomatitis | Original: Selenium vs control; target: Selenium supplementation vs control; reversed: no. | Independent comparison groups; original outcome definition, threshold, and time point retained; no OR/RR or incompatible outcome mixing. | yes | Verified against the study report; comparison direction and synthesis role checked |
| Buentzel 2010 | Maximal dysphagia | Original: Selenium vs control; target: Selenium supplementation vs control; reversed: no. | Independent comparison groups; original outcome definition, threshold, and time point retained; no OR/RR or incompatible outcome mixing. | yes | Verified against the study report; comparison direction and synthesis role checked |
| Asfour 2006 | Infection rate | Original: Control vs selenium in results text; target: Selenium supplementation vs control; reversal=yes. | Independent comparison groups; original outcome definition, threshold, and time point retained; no OR/RR or incompatible outcome mixing. | yes | Verified against the study report; comparison direction and synthesis role checked |
| Asfour 2006 | Cardiac ejection fraction | Original: Within selenium group before vs after as reported; target: Selenium supplementation vs control; reversed: no. | Independent comparison groups; original outcome definition, threshold, and time point retained; no OR/RR or incompatible outcome mixing. | yes | Verified against the study report; comparison direction and synthesis role checked |
| Hu 1997 | Peripheral WBC count | Original: Se cycle vs control cycle; target: Selenium supplementation vs control; reversed: no. | Independent comparison groups; original outcome definition, threshold, and time point retained; no OR/RR or incompatible outcome mixing. | yes | Verified against the study report; comparison direction and synthesis role checked |
| Hu 1997 | GCSF consumption | Original: Se cycle vs control cycle; target: Selenium supplementation vs control; reversed: no. | Independent comparison groups; original outcome definition, threshold, and time point retained; no OR/RR or incompatible outcome mixing. | yes | Verified against the study report; comparison direction and synthesis role checked |
| Hu 1997 | Urine NAG | Original: Se cycle vs control cycle; target: Selenium supplementation vs control; reversed: no. | Independent comparison groups; original outcome definition, threshold, and time point retained; no OR/RR or incompatible outcome mixing. | yes | Verified against the study report; comparison direction and synthesis role checked |
| Hu 1997 | Urine GGT | Original: Se cycle vs control cycle; target: Selenium supplementation vs control; reversed: no. | Independent comparison groups; original outcome definition, threshold, and time point retained; no OR/RR or incompatible outcome mixing. | yes | Verified against the study report; comparison direction and synthesis role checked |
| Ameri 2016 | Mean mucositis score | Original: Selenium vs placebo; target: Selenium supplementation vs control; reversed: no. | Independent comparison groups; original outcome definition, threshold, and time point retained; no OR/RR or incompatible outcome mixing. | no | Verified against the study report; comparison direction and synthesis role checked |
| Ameri 2016 | Mean mucositis score | Original: Selenium vs placebo; target: Selenium supplementation vs control; reversed: no. | Independent comparison groups; original outcome definition, threshold, and time point retained; no OR/RR or incompatible outcome mixing. | no | Verified against the study report; comparison direction and synthesis role checked |
| Ameri 2016 | Mean mucositis score | Original: Selenium vs placebo; target: Selenium supplementation vs control; reversed: no. | Independent comparison groups; original outcome definition, threshold, and time point retained; no OR/RR or incompatible outcome mixing. | no | Verified against the study report; comparison direction and synthesis role checked |
| Chen 2023 | Any adverse reaction grade I-IV | Original: Observation group vs Control group; target: Selenium supplementation vs no selenium/placebo/control; reversed: no. | Independent comparison groups; original outcome definition, threshold, and time point retained; no OR/RR or incompatible outcome mixing. | yes | Verified against the study report; comparison direction and synthesis role checked |
| Chen 2023 | Grade III-IV adverse reactions | Original: Observation group vs Control group; target: Selenium supplementation vs no selenium/placebo/control; reversed: no. | Independent comparison groups; original outcome definition, threshold, and time point retained; no OR/RR or incompatible outcome mixing. | yes | Verified against the study report; comparison direction and synthesis role checked |
| Chen 2023 | Gastrointestinal reactions | Original: Observation group vs Control group; target: Selenium supplementation vs no selenium/placebo/control; reversed: no. | Independent comparison groups; original outcome definition, threshold, and time point retained; no OR/RR or incompatible outcome mixing. | yes | Verified against the study report; comparison direction and synthesis role checked |
| Chen 2023 | Bone marrow suppression | Original: Observation group vs Control group; target: Selenium supplementation vs no selenium/placebo/control; reversed: no. | Independent comparison groups; original outcome definition, threshold, and time point retained; no OR/RR or incompatible outcome mixing. | yes | Verified against the study report; comparison direction and synthesis role checked |
| Chen 2023 | Pain grade >=2 | Original: Observation group vs Control group; target: Selenium supplementation vs no selenium/placebo/control; reversed: no. | Independent comparison groups; original outcome definition, threshold, and time point retained; no OR/RR or incompatible outcome mixing. | yes | Verified against the study report; comparison direction and synthesis role checked |
| Wang 2020 | Severe oral mucositis | Original: Selenium yeast group vs Routine treatment group; target: Selenium supplementation vs no selenium/placebo/control; reversed: no. | Independent comparison groups; original outcome definition, threshold, and time point retained; no OR/RR or incompatible outcome mixing. | no | Verified against the study report; comparison direction and synthesis role checked |
| Wang 2020 | Oral mucositis onset time | Original: Selenium yeast group vs Routine treatment group; target: Selenium supplementation vs no selenium/placebo/control; reversed: no. | Independent comparison groups; original outcome definition, threshold, and time point retained; no OR/RR or incompatible outcome mixing. | no | Verified against the study report; comparison direction and synthesis role checked |
| Wang 2020 | Pain grade >=2 | Original: Selenium yeast group vs Routine treatment group; target: Selenium supplementation vs no selenium/placebo/control; reversed: no. | Independent comparison groups; original outcome definition, threshold, and time point retained; no OR/RR or incompatible outcome mixing. | no | Verified against the study report; comparison direction and synthesis role checked |
| Wang 2020 | Pain relief time | Original: Selenium yeast group vs Routine treatment group; target: Selenium supplementation vs no selenium/placebo/control; reversed: no. | Independent comparison groups; original outcome definition, threshold, and time point retained; no OR/RR or incompatible outcome mixing. | no | Verified against the study report; comparison direction and synthesis role checked |
| Son 2017 | Serum amylase 2 days after I-131 | Original: Selenium supplement group vs Control group; target: Selenium supplementation vs no selenium/placebo/control; reversal: not applicable. | Independent comparison groups; original outcome definition, threshold, and time point retained; no OR/RR or incompatible outcome mixing. | no | Verified against the study report; comparison direction and synthesis role checked |
| Son 2017 | Questionnaire score 6 months after I-131 | Original: Selenium supplement group vs Control group; target: Selenium supplementation vs no selenium/placebo/control; reversal: not applicable. | Independent comparison groups; original outcome definition, threshold, and time point retained; no OR/RR or incompatible outcome mixing. | no | Verified against the study report; comparison direction and synthesis role checked |
| Son 2017 | Salivary gland scintigraphy parameters | Original: Selenium vs control; target: Selenium supplementation vs no selenium/placebo/control; reversal: not applicable. | Independent comparison groups; original outcome definition, threshold, and time point retained; no OR/RR or incompatible outcome mixing. | no | Verified against the study report; comparison direction and synthesis role checked |
| Bahador 2022 | Diarrhea grade II/III vs grade 0 | Original: Control/no selenium vs selenium, inferred from results wording; target: Selenium supplementation vs no selenium/placebo/control; reversed: no. | Independent comparison groups; original outcome definition, threshold, and time point retained; no OR/RR or incompatible outcome mixing. | yes | Verified against the study report; comparison direction and synthesis role checked |
| Bahador 2022 | Diarrhea grade II/III vs grade I | Original: Control/no selenium vs selenium, inferred from results wording; target: Selenium supplementation vs no selenium/placebo/control; reversed: no. | Independent comparison groups; original outcome definition, threshold, and time point retained; no OR/RR or incompatible outcome mixing. | yes | Verified against the study report; comparison direction and synthesis role checked |
| Bahador 2022 | Diarrhea grade I vs grade 0 | Original: Control/no selenium vs selenium, inferred from results wording; target: Selenium supplementation vs no selenium/placebo/control; reversed: no. | Independent comparison groups; original outcome definition, threshold, and time point retained; no OR/RR or incompatible outcome mixing. | yes | Verified against the study report; comparison direction and synthesis role checked |
| Bahador 2022 | Pain | Original: Control/no selenium vs selenium, inferred from results wording; target: Selenium supplementation vs no selenium/placebo/control; reversed: no. | Independent comparison groups; original outcome definition, threshold, and time point retained; no OR/RR or incompatible outcome mixing. | yes | Verified against the study report; comparison direction and synthesis role checked |
| Pavlidis 2026 | Electrogustometry thresholds | Original: Selenium vs control; target: Selenium supplementation vs no selenium/placebo/control; reversal: not applicable. | Independent comparison groups; original outcome definition, threshold, and time point retained; no OR/RR or incompatible outcome mixing. | no | Verified against the study report; comparison direction and synthesis role checked |
| Pavlidis 2026 | fPap morphology vs EGM thresholds | Original: Selenium vs control; target: Selenium supplementation vs no selenium/placebo/control; reversal: not applicable. | Independent comparison groups; original outcome definition, threshold, and time point retained; no OR/RR or incompatible outcome mixing. | yes | Verified against the study report; comparison direction and synthesis role checked |

Note. No OR was converted to RR. Continuity correction was applied only as specified in the statistical methods.

**Supplementary Table S7. Analysis-set membership and evidence roles**

**Panel A. Main, sensitivity, and subgroup roles**

| **Study** | **Outcome** | **Main analysis** | **Sensitivity analysis** | **Subgroup analysis** |
| --- | --- | --- | --- | --- |
| Laali 2020; Mix 2015; Buentzel 2010; Wang 2020 | Four independent head-and-neck RT/CRT severe OM/stomatitis event-count rows available after adding Wang 2020; random-effects DL by protocol with placebo/non-placebo sensitivity. | Yes - sole primary meta-analysis | RoB/comparator sensitivity analyses and robust model checks were conducted where feasible | No formal subgroup test; comparator/RoB subsets treated as sensitivity analyses where feasible. |
| Laali 2020; Mix 2015; Buentzel 2010; Wang 2020; Jahangard-Rafsanjani 2013 | Five independent severe OM rows available after adding Wang 2020 and HSCT context; exploratory random-effects DL because therapy contexts differ. | No | RoB/comparator sensitivity analyses and robust model checks were conducted where feasible | No formal subgroup test; comparator/RoB subsets treated as sensitivity analyses where feasible. |
| Laali 2020; Jahangard-Rafsanjani 2013 | Two any-OM rows available; exploratory fixed-effect only because event rates are saturated and contexts differ. | No | RoB/comparator sensitivity analyses and robust model checks were conducted where feasible | No formal subgroup test; comparator/RoB subsets treated as sensitivity analyses where feasible. |
| Laali 2020; Jahangard-Rafsanjani 2013 | Two duration-in-days rows available; arm sizes obtained from the study-level participant records; pooled only as scope-sensitive exploratory MD because severity thresholds and therapy contexts differ. | No | RoB/comparator sensitivity analyses and robust model checks were conducted where feasible | No formal subgroup test; comparator/RoB subsets treated as sensitivity analyses where feasible. |
| Laali 2020; Wang 2020 | Does selenium alter time to oral mucositis onset when compatible arm-level means and SDs are available? | No | REML/Hartung-Knapp robustness required | No formal subgroup test; comparator/RoB subsets treated as sensitivity analyses where feasible. |
| Yang 2023; Laali 2020 | Two grade 3 hematologic toxicity rows available; exploratory fixed-effect only. | No | RoB/comparator sensitivity analyses and robust model checks were conducted where feasible | No formal subgroup test; comparator/RoB subsets treated as sensitivity analyses where feasible. |
| Yang 2023; Laali 2020; Mix 2015; Chen 2023 | Four hematologic toxicity rows available after adding Chen 2023 and Mix 2015; exploratory random-effects DL only because severity thresholds differ. | No | RoB/comparator sensitivity analyses and robust model checks were conducted where feasible | No formal subgroup test; comparator/RoB subsets treated as sensitivity analyses where feasible. |
| Yang 2023; Muecke 2010 | Two diarrhea event rows available; broad exploratory fixed-effect only because severity/time contexts differ. | No | RoB/comparator sensitivity analyses and robust model checks were conducted where feasible | No formal subgroup test; comparator/RoB subsets treated as sensitivity analyses where feasible. |
| Yang 2023; Muecke 2010; Chen 2023 | Three GI/diarrhea rows available after adding Chen 2023; exploratory random-effects DL only because diarrhea and GI reactions are not identical. | No | RoB/comparator sensitivity analyses and robust model checks were conducted where feasible | No formal subgroup test; comparator/RoB subsets treated as sensitivity analyses where feasible. |
| Studies contributing to the corresponding analysis | Bahador 2022 and Muecke 2010 were assessed for a same-direction grade 2-3 versus grade 0 pelvic-diarrhea OR signal. | No | Yes; fixed-effect OR with REML/Hartung-Knapp robustness, kept separate from RR pools | No formal subgroup test; comparator/RoB subsets treated as sensitivity analyses where feasible. |
| Ghorbani 2013; Jahangard-Rafsanjani 2013 | Two renal event rows available; broad exploratory fixed-effect only because definitions and therapy contexts differ. | No | RoB/comparator sensitivity analyses and robust model checks were conducted where feasible | No formal subgroup test; comparator/RoB subsets treated as sensitivity analyses where feasible. |
| Chen 2023; Wang 2020 | Two grade >=2 pain rows available, but pain etiology differs; scope-sensitive fixed-effect exploratory pool only. | No | RoB/comparator sensitivity analyses and robust model checks were conducted where feasible | No formal subgroup test; comparator/RoB subsets treated as sensitivity analyses where feasible. |
| Yang 2023; Laali 2020; Mix 2015; Jahangard-Rafsanjani 2013; Buentzel 2010; Chen 2023; Wang 2020 | Seven severe or grade >=3/III-IV harmful-event rows available; exploratory random-effects DL only because toxicity domains differ. | No | RoB/comparator sensitivity analyses and robust model checks were conducted where feasible | No formal subgroup test; comparator/RoB subsets treated as sensitivity analyses where feasible. |
| Yim 2026; Yang 2023; Laali 2020; Mix 2015; Jahangard-Rafsanjani 2013; Ghorbani 2013; Muecke 2010; Buentzel 2010; Asfour 2006; Chen 2023; Wang 2020 | Eleven studies contribute one selected harmful event each after adding Chen 2023 and Wang 2020; broad exploratory random-effects DL only. | No | RoB/comparator sensitivity analyses and robust model checks were conducted where feasible | No formal subgroup test; comparator/RoB subsets treated as sensitivity analyses where feasible. |
| Studies contributing to the corresponding analysis | Yim 2026 provides event data and exact CIPN20 supplemental means/SDs, but no compatible second selenium trial exists. | No | Not applicable because no pooled estimate was calculated | No formal subgroup test; comparator/RoB subsets treated as sensitivity analyses where feasible. |
| Studies contributing to the corresponding analysis | Son 2017 reports salivary gland injury as median/range and scintigraphy summaries; no mean/SD or event-count pool. | No | Not applicable because no pooled estimate was calculated | No formal subgroup test; comparator/RoB subsets treated as sensitivity analyses where feasible. |
| Studies contributing to the corresponding analysis | Pavlidis 2026 provides model-predicted EMMs and adjusted pairwise EGM contrasts, but no compatible second EGM trial exists. | No | Not applicable because no pooled estimate was calculated | No formal subgroup test; comparator/RoB subsets treated as sensitivity analyses where feasible. |
| Studies contributing to the corresponding analysis | Severe oral mucositis with Orvain 2025 conference augmentation | No | Yes; baseline model plus REML/Hartung-Knapp robustness | No formal subgroup test; comparator/RoB subsets treated as sensitivity analyses where feasible. |
| Studies contributing to the corresponding analysis | Transplant-setting severe oral mucositis | No | Yes; baseline model plus REML/Hartung-Knapp robustness | No formal subgroup test; comparator/RoB subsets treated as sensitivity analyses where feasible. |
| Studies contributing to the corresponding analysis | Diarrhea with Orvain 2025 conference augmentation | No | Yes; baseline model plus REML/Hartung-Knapp robustness | No formal subgroup test; comparator/RoB subsets treated as sensitivity analyses where feasible. |
| Studies contributing to the corresponding analysis | Broad toxicity with Orvain 2025 conference augmentation | No | Yes; baseline model plus REML/Hartung-Knapp robustness | No formal subgroup test; comparator/RoB subsets treated as sensitivity analyses where feasible. |

**Panel B. Exploratory, narrative, and no-pooling decisions**

| **Exploratory or supplementary role** | **Narrative-only role** | **Exclusion from quantitative synthesis reason** | **Decision rationale** |
| --- | --- | --- | --- |
| No pooled exploratory estimate | No | Not applicable | controlled by narrow population, toxicity threshold, and RR definition |
| exploratory; add scope-sensitive where applicable | No | Not applicable | Five independent severe OM rows available after adding Wang 2020 and HSCT context; exploratory random-effects DL because therapy contexts differ. |
| exploratory; add scope-sensitive where applicable | No | Not applicable | Two any-OM rows available; exploratory fixed-effect only because event rates are saturated and contexts differ. |
| exploratory; add scope-sensitive where applicable | No | Not applicable | Two duration-in-days rows available; arm sizes obtained from the study-level participant records; pooled only as scope-sensitive exploratory MD because severity thresholds and therapy contexts differ. |
| No pooled exploratory estimate | No | Not applicable | Laali reports weeks and Wang reports days; multiplying both Laali means and SDs by seven preserves the construct and variance scale. |
| exploratory; add scope-sensitive where applicable | No | Not applicable | Two grade 3 hematologic toxicity rows available; exploratory fixed-effect only. |
| exploratory; add scope-sensitive where applicable | No | Not applicable | Four hematologic toxicity rows available after adding Chen 2023 and Mix 2015; exploratory random-effects DL only because severity thresholds differ. |
| exploratory; add scope-sensitive where applicable | No | Not applicable | Two diarrhea event rows available; broad exploratory fixed-effect only because severity/time contexts differ. |
| exploratory; add scope-sensitive where applicable | No | Not applicable | Three GI/diarrhea rows available after adding Chen 2023; exploratory random-effects DL only because diarrhea and GI reactions are not identical. |
| derived exploratory OR sensitivity; no-selenium versus selenium | Yes | Never combine this OR synthesis with event-count RR pools or present it as confirmatory. | Bahador supplies a reported OR and rounded P value; Muecke supplies exact week-4 grade counts for a reconstructed unadjusted OR. |
| exploratory; add scope-sensitive where applicable | No | Not applicable | Two renal event rows available; broad exploratory fixed-effect only because definitions and therapy contexts differ. |
| exploratory; add scope-sensitive where applicable | No | Not applicable | Two grade >=2 pain rows available, but pain etiology differs; scope-sensitive fixed-effect exploratory pool only. |
| exploratory; add scope-sensitive where applicable | No | Not applicable | Seven severe or grade >=3/III-IV harmful-event rows available; exploratory random-effects DL only because toxicity domains differ. |
| exploratory; add scope-sensitive where applicable | No | Not applicable | Eleven studies contribute one selected harmful event each after adding Chen 2023 and Wang 2020; broad exploratory random-effects DL only. |
| No pooled exploratory estimate | Yes | Do not convert OR to RR, median/range to mean/SD, model-only results to arm data, or a single study into a pooled estimate. | Observational Alhazmi data use a different design, scale, and time point and are not combined with the randomized Yim trial. |
| No pooled exploratory estimate | Yes | Do not convert OR to RR, median/range to mean/SD, model-only results to arm data, or a single study into a pooled estimate. | Son 2017 reports salivary gland injury as median/range and scintigraphy summaries; no mean/SD or event-count pool. |
| No pooled exploratory estimate | Yes | Do not combine EGM thresholds with mucositis scores or event risks. | Model-based serial EGM contrasts are not interchangeable with oral-mucositis event counts or unadjusted arm-level MDs. |
| conference-augmented supplementary sensitivity | No | Not applicable | expanded severe-OM full-report pool plus Orvain grade 3-4 events |
| conference-augmented supplementary sensitivity | No | Not applicable | Jahangard-Rafsanjani full report plus Orvain conference abstract |
| conference-augmented supplementary sensitivity | No | Not applicable | threshold-mixed diarrhea pool plus Orvain grade 3-4 events |
| conference-augmented supplementary sensitivity | No | Not applicable | accepted broad pool plus Orvain grade 3-4 all-adverse-event events |

*Note. The primary analysis was restricted to severe oral mucositis in head-and-neck RT/CRT. Broader pools were exploratory; OR-only, single-study, and incompatible outcomes were not forced into RR or MD pools.*

**Supplementary Table S8. Complete quantitative synthesis results**

**Panel A. Accepted-model estimates and heterogeneity**

| **Outcome** | **Analysis role** | **Studies** | **Participants or events** | **Effect measure** | **Model** | **Pooled estimate** | **95% CI or CrI** | **P value** | **Heterogeneity statistic** |
| --- | --- | --- | --- | --- | --- | --- | --- | --- | --- |
| severe oral mucositis, head-and-neck RT/CRT | Primary | 4 | N=188; events 40/99 (selenium) and 39/89 (control) | RR | DerSimonian-Laird random effects | 0.89 | 0.50 to 1.56 | 0.679 | I2=44.9%; Q(3)=5.45, p=0.142; tau2=0.149 |
| severe oral mucositis, expanded contexts | Exploratory | 5 | N=262; events 44/136 (selenium) and 52/126 (control) | RR | DerSimonian-Laird random effects | 0.71 | 0.38 to 1.33 | 0.287 | I2=60.9%; Q(4)=10.24, p=0.037; tau2=0.287 |
| any oral mucositis | Exploratory | 2 | N=145; events 67/74 (selenium) and 64/71 (control) | RR | fixed-effect inverse variance (two-study protocol-compatible) | 0.98 | 0.91 to 1.05 | 0.599 | I2=0.0%; Q(1)=0.26, p=0.607 |
| grade 3 hematologic toxicity | Exploratory | 2 | N=138; events 26/68 (selenium) and 40/70 (control) | RR | fixed-effect inverse variance (two-study protocol-compatible) | 0.70 | 0.50 to 0.97 | 0.033 | I2=0.0%; Q(1)=0.04, p=0.846 |
| expanded hematologic toxicity | Exploratory | 4 | N=242; events 30/121 (selenium) and 46/121 (control) | RR | DerSimonian-Laird random effects | 0.70 | 0.50 to 0.96 | 0.028 | I2=0.0%; Q(3)=1.40, p=0.706; tau2=0.000 |
| diarrhea event | Exploratory | 2 | N=185; events 17/89 (selenium) and 29/96 (control) | RR | fixed-effect inverse variance (two-study protocol-compatible) | 0.60 | 0.37 to 0.98 | 0.040 | I2=47.3%; Q(1)=1.90, p=0.168 |
| gastrointestinal toxicity or diarrhea | Exploratory | 3 | N=271; events 30/132 (selenium) and 50/139 (control) | RR | DerSimonian-Laird random effects | 0.61 | 0.42 to 0.88 | 0.007 | I2=0.0%; Q(2)=1.91, p=0.385; tau2=0.000 |
| renal toxicity event | Exploratory | 2 | N=196; events 3/98 (selenium) and 14/98 (control) | RR | fixed-effect inverse variance (two-study protocol-compatible) | 0.31 | 0.10 to 1.00 | 0.051 | I2=27.1%; Q(1)=1.37, p=0.241 |
| pain grade >=2 | Exploratory | 2 | N=146; events 41/73 (selenium) and 56/73 (control) | RR | fixed-effect inverse variance (two-study protocol-compatible) | 0.78 | 0.64 to 0.95 | 0.015 | I2=31.6%; Q(1)=1.46, p=0.227 |
| severe broad toxicity | Exploratory | 7 | N=452; events 68/229 (selenium) and 92/223 (control) | RR | DerSimonian-Laird random effects | 0.71 | 0.46 to 1.09 | 0.116 | I2=53.8%; Q(6)=12.98, p=0.043; tau2=0.138 |
| broad toxicity-prevention pool | Exploratory | 11 | N=745; events 99/374 (selenium) and 163/371 (control) | RR | DerSimonian-Laird random effects | 0.60 | 0.42 to 0.83 | 0.003 | I2=55.5%; Q(10)=22.48, p=0.013; tau2=0.140 |
| oral mucositis duration in days | Scope-sensitive exploratory | 2 | N=145 | MD | fixed-effect inverse variance (two-study protocol-compatible) | -1.72 | -2.64 to -0.80 | <0.001 | I2=0.0%; Q(1)=0.15, p=0.695 |
| oral mucositis onset in days | Exploratory | 2 | N=131 | MD | fixed-effect inverse variance (two-study protocol-compatible) | 0.98 | -0.44 to 2.39 | 0.178 | I2=23.2%; Q(1)=1.30, p=0.254 |

**Panel B. Prediction intervals, certainty, robustness, and figure links**

| **Outcome** | **Prediction interval if used** | **Certainty or caveat** | **Figure or supplement link** |
| --- | --- | --- | --- |
| severe oral mucositis, head-and-neck RT/CRT | REML prediction interval 0.18 to 4.30; interpret cautiously with small k | GRADE Very low; REML/Hartung-Knapp RR 0.88 (0.35 to 2.23). Primary estimate remains inconclusive. | Figure 2 |
| severe oral mucositis, expanded contexts | REML prediction interval 0.13 to 3.96; interpret cautiously with small k | GRADE Very low; REML/Hartung-Knapp RR 0.71 (0.30 to 1.67). Exploratory result; not a confirmatory efficacy conclusion. | Supplementary Figure S1 |
| any oral mucositis | Not calculated for the fixed-effect accepted model | GRADE Low; REML/Hartung-Knapp RR 0.98 (0.77 to 1.24). Exploratory result; not a confirmatory efficacy conclusion. | Supplementary Figure S2 |
| grade 3 hematologic toxicity | Not calculated for the fixed-effect accepted model | GRADE Low; REML/Hartung-Knapp RR 0.70 (0.46 to 1.06). Conventional null-exclusion was not retained under Hartung-Knapp; model-sensitive. | Supplementary Figure S3 |
| expanded hematologic toxicity | REML prediction interval 0.49 to 1.00; interpret cautiously with small k | GRADE Very low; REML/Hartung-Knapp RR 0.70 (0.49 to 1.00). Exploratory result; not a confirmatory efficacy conclusion. | Supplementary Figure S4 |
| diarrhea event | Not calculated for the fixed-effect accepted model | GRADE Very low; REML/Hartung-Knapp RR 0.70 (0.00 to 204.19). Conventional null-exclusion was not retained under Hartung-Knapp; model-sensitive. | Supplementary Figure S5 |
| gastrointestinal toxicity or diarrhea | REML prediction interval 0.28 to 1.33; interpret cautiously with small k | GRADE Very low; REML/Hartung-Knapp RR 0.61 (0.28 to 1.33). Conventional null-exclusion was not retained under Hartung-Knapp; model-sensitive. | Supplementary Figure S6 |
| renal toxicity event | Not calculated for the fixed-effect accepted model | GRADE Very low; REML/Hartung-Knapp RR 0.27 (8.59e-06 to 8202.54). Exploratory result; not a confirmatory efficacy conclusion. | Supplementary Figure S7 |
| pain grade >=2 | Not calculated for the fixed-effect accepted model | GRADE Low; REML/Hartung-Knapp RR 0.74 (0.09 to 5.98). Conventional null-exclusion was not retained under Hartung-Knapp; model-sensitive. | Supplementary Figure S8 |
| severe broad toxicity | REML prediction interval 0.22 to 2.21; interpret cautiously with small k | GRADE Very low; REML/Hartung-Knapp RR 0.70 (0.40 to 1.23). Exploratory result; not a confirmatory efficacy conclusion. | Supplementary Figure S9 |
| broad toxicity-prevention pool | REML prediction interval 0.24 to 1.50; interpret cautiously with small k | GRADE Very low; REML/Hartung-Knapp RR 0.60 (0.40 to 0.89). Exploratory result; not a confirmatory efficacy conclusion. | Supplementary Figure S10 |
| oral mucositis duration in days | Not calculated for the fixed-effect accepted model | GRADE Very low; REML/Hartung-Knapp MD -1.72 (-4.06 to 0.62). Conventional null-exclusion was not retained under Hartung-Knapp; model-sensitive. | Supplementary Figure S11 |
| oral mucositis onset in days | Not calculated for the fixed-effect accepted model | GRADE Very low; REML/Hartung-Knapp MD 0.84 (-10.70 to 12.38). Exploratory result; not a confirmatory efficacy conclusion. | Supplementary Figure S14 |

*Note. Accepted baseline models are reported as the primary numerical results. REML/Hartung-Knapp robustness results appear in the certainty/caveat column and Supplementary Table S9.*

**Supplementary Table S9. Sensitivity and model-robustness analyses**

**Panel A. Sensitivity and robustness estimates**

| **Outcome** | **Analysis type** | **Change from main analysis** | **Studies** | **Effect measure** | **Pooled estimate** | **95% CI or CrI** |
| --- | --- | --- | --- | --- | --- | --- |
| Primary: severe oral mucositis, head-and-neck RT/CRT | Comparator/RoB subset sensitivity | High-risk studies excluded | 3 | RR | 0.73 | 0.34 to 1.56 |
| Primary: severe oral mucositis, head-and-neck RT/CRT | Comparator/RoB subset sensitivity | Placebo-controlled studies only | 2 | RR | 1.10 | 0.78 to 1.57 |
| Exploratory: severe oral mucositis, expanded contexts | Comparator/RoB subset sensitivity | High-risk studies excluded | 4 | RR | 0.58 | 0.27 to 1.25 |
| Exploratory: severe oral mucositis, expanded contexts | Comparator/RoB subset sensitivity | Placebo-controlled studies only | 3 | RR | 0.64 | 0.25 to 1.64 |
| Exploratory: any oral mucositis | Comparator/RoB subset sensitivity | High-risk studies excluded | 2 | RR | 0.98 | 0.91 to 1.05 |
| Exploratory: any oral mucositis | Comparator/RoB subset sensitivity | Placebo-controlled studies only | 2 | RR | 0.98 | 0.91 to 1.05 |
| Exploratory: grade 3 hematologic toxicity | Comparator/RoB subset sensitivity | High-risk studies excluded | 2 | RR | 0.70 | 0.50 to 0.97 |
| Exploratory: grade 3 hematologic toxicity | Comparator/RoB subset sensitivity | Placebo-controlled studies only | 2 | RR | 0.70 | 0.50 to 0.97 |
| Exploratory: expanded hematologic toxicity | Comparator/RoB subset sensitivity | High-risk studies excluded | 4 | RR | 0.70 | 0.50 to 0.96 |
| Exploratory: expanded hematologic toxicity | Comparator/RoB subset sensitivity | Placebo-controlled studies only | 3 | RR | 0.71 | 0.51 to 0.99 |
| Exploratory: diarrhea event | Comparator/RoB subset sensitivity | High-risk studies excluded | 1 | RR | Not estimable | Not estimable |
| Exploratory: diarrhea event | Comparator/RoB subset sensitivity | Placebo-controlled studies only | 1 | RR | Not estimable | Not estimable |
| Exploratory: gastrointestinal toxicity or diarrhea | Comparator/RoB subset sensitivity | High-risk studies excluded | 2 | RR | 0.70 | 0.42 to 1.16 |
| Exploratory: gastrointestinal toxicity or diarrhea | Comparator/RoB subset sensitivity | Placebo-controlled studies only | 1 | RR | Not estimable | Not estimable |
| Exploratory: renal toxicity event | Comparator/RoB subset sensitivity | High-risk studies excluded | 2 | RR | 0.31 | 0.10 to 1.00 |
| Exploratory: renal toxicity event | Comparator/RoB subset sensitivity | Placebo-controlled studies only | 2 | RR | 0.31 | 0.10 to 1.00 |
| Exploratory: pain grade >=2 | Comparator/RoB subset sensitivity | High-risk studies excluded | 2 | RR | 0.78 | 0.64 to 0.95 |
| Exploratory: pain grade >=2 | Comparator/RoB subset sensitivity | Placebo-controlled studies only | 0 | RR | Not estimable | Not estimable |
| Exploratory: severe broad toxicity | Comparator/RoB subset sensitivity | High-risk studies excluded | 6 | RR | 0.64 | 0.41 to 1.01 |
| Exploratory: severe broad toxicity | Comparator/RoB subset sensitivity | Placebo-controlled studies only | 4 | RR | 0.73 | 0.45 to 1.18 |
| Exploratory: broad toxicity-prevention pool | Comparator/RoB subset sensitivity | High-risk studies excluded | 8 | RR | 0.59 | 0.40 to 0.88 |
| Exploratory: broad toxicity-prevention pool | Comparator/RoB subset sensitivity | Placebo-controlled studies only | 6 | RR | 0.58 | 0.34 to 1.02 |
| Primary: severe oral mucositis, head-and-neck RT/CRT | REML/Hartung-Knapp robustness analysis | REML heterogeneity estimator with Hartung-Knapp | 4 | RR | 0.88 | 0.35 to 2.23 |
| Exploratory: severe oral mucositis, expanded contexts | REML/Hartung-Knapp robustness analysis | REML heterogeneity estimator with Hartung-Knapp | 5 | RR | 0.71 | 0.30 to 1.67 |
| Exploratory: any oral mucositis | REML/Hartung-Knapp robustness analysis | REML heterogeneity estimator with Hartung-Knapp | 2 | RR | 0.98 | 0.77 to 1.24 |
| Exploratory: grade 3 hematologic toxicity | REML/Hartung-Knapp robustness analysis | REML heterogeneity estimator with Hartung-Knapp | 2 | RR | 0.70 | 0.46 to 1.06 |
| Exploratory: expanded hematologic toxicity | REML/Hartung-Knapp robustness analysis | REML heterogeneity estimator with Hartung-Knapp | 4 | RR | 0.70 | 0.49 to 1.00 |
| Exploratory: diarrhea event | REML/Hartung-Knapp robustness analysis | REML heterogeneity estimator with Hartung-Knapp | 2 | RR | 0.70 | 0.00 to 204.19 |
| Exploratory: gastrointestinal toxicity or diarrhea | REML/Hartung-Knapp robustness analysis | REML heterogeneity estimator with Hartung-Knapp | 3 | RR | 0.61 | 0.28 to 1.33 |
| Exploratory: renal toxicity event | REML/Hartung-Knapp robustness analysis | REML heterogeneity estimator with Hartung-Knapp | 2 | RR | 0.27 | 8.59e-06 to 8202.54 |
| Exploratory: pain grade >=2 | REML/Hartung-Knapp robustness analysis | REML heterogeneity estimator with Hartung-Knapp | 2 | RR | 0.74 | 0.09 to 5.98 |
| Exploratory: severe broad toxicity | REML/Hartung-Knapp robustness analysis | REML heterogeneity estimator with Hartung-Knapp | 7 | RR | 0.70 | 0.40 to 1.23 |
| Exploratory: broad toxicity-prevention pool | REML/Hartung-Knapp robustness analysis | REML heterogeneity estimator with Hartung-Knapp | 11 | RR | 0.60 | 0.40 to 0.89 |
| Exploratory: oral mucositis duration in days | REML/Hartung-Knapp robustness analysis | REML heterogeneity estimator with Hartung-Knapp | 2 | MD | -1.72 | -4.06 to 0.62 |
| Exploratory: oral mucositis onset in days | REML/Hartung-Knapp robustness analysis | REML heterogeneity estimator with Hartung-Knapp | 2 | MD | 0.84 | -10.70 to 12.38 |
| Severe oral mucositis with conference-abstract augmentation | Conference-augmented sensitivity analysis | Adds Orvain 2025 conference-abstract arm-level data | 6 | RR | 0.83 | 0.56 to 1.23 |
| Severe oral mucositis with conference-abstract augmentation | Conference-augmented sensitivity analysis | Adds Orvain 2025 conference-abstract arm-level data | 6 | RR | 0.80 | 0.44 to 1.48 |
| Severe oral mucositis in transplant settings | Conference-augmented sensitivity analysis | Adds Orvain 2025 conference-abstract arm-level data | 2 | RR | 0.90 | 0.66 to 1.22 |
| Severe oral mucositis in transplant settings | Conference-augmented sensitivity analysis | Adds Orvain 2025 conference-abstract arm-level data | 2 | RR | 0.62 | 3.88e-04 to 977.21 |
| Diarrhea with conference-abstract augmentation | Conference-augmented sensitivity analysis | Adds Orvain 2025 conference-abstract arm-level data | 3 | RR | 0.60 | 0.39 to 0.94 |
| Diarrhea with conference-abstract augmentation | Conference-augmented sensitivity analysis | Adds Orvain 2025 conference-abstract arm-level data | 3 | RR | 0.60 | 0.23 to 1.56 |
| Broad toxicity-prevention pool with conference-abstract augmentation | Conference-augmented sensitivity analysis | Adds Orvain 2025 conference-abstract arm-level data | 12 | RR | 0.66 | 0.48 to 0.90 |
| Broad toxicity-prevention pool with conference-abstract augmentation | Conference-augmented sensitivity analysis | Adds Orvain 2025 conference-abstract arm-level data | 12 | RR | 0.65 | 0.44 to 0.96 |
| Pelvic chemoradiotherapy grade 2-3 diarrhea | Derived OR sensitivity analysis | Combines reported Bahador OR with reconstructed Muecke category-contrast OR | 2 | OR | 3.76 | 1.78 to 7.94 |
| Pelvic chemoradiotherapy grade 2-3 diarrhea | Derived OR sensitivity analysis | Combines reported Bahador OR with reconstructed Muecke category-contrast OR | 2 | OR | 3.76 | 1.51 to 9.33 |

**Panel B. Heterogeneity, rationale, and conclusion impact**

| **Outcome** | **Analysis type** | **Heterogeneity** | **Reason performed** | **Conclusion impact** | **Role label** |
| --- | --- | --- | --- | --- | --- |
| Primary: severe oral mucositis, head-and-neck RT/CRT | Comparator/RoB subset sensitivity | I2=57.8% | Assess sensitivity to final RoB judgment and comparator type. | Sensitivity result only; it does not change the primary inference. | Primary sensitivity |
| Primary: severe oral mucositis, head-and-neck RT/CRT | Comparator/RoB subset sensitivity | I2=0.0% | Assess sensitivity to final RoB judgment and comparator type. | Sensitivity result only; it does not change the primary inference. | Primary sensitivity |
| Exploratory: severe oral mucositis, expanded contexts | Comparator/RoB subset sensitivity | I2=67.2% | Assess sensitivity to final RoB judgment and comparator type. | Exploratory sensitivity result; no promotion to a confirmatory conclusion. | Exploratory sensitivity |
| Exploratory: severe oral mucositis, expanded contexts | Comparator/RoB subset sensitivity | I2=68.1% | Assess sensitivity to final RoB judgment and comparator type. | Exploratory sensitivity result; no promotion to a confirmatory conclusion. | Exploratory sensitivity |
| Exploratory: any oral mucositis | Comparator/RoB subset sensitivity | I2=0.0% | Assess sensitivity to final RoB judgment and comparator type. | Exploratory sensitivity result; no promotion to a confirmatory conclusion. | Exploratory sensitivity |
| Exploratory: any oral mucositis | Comparator/RoB subset sensitivity | I2=0.0% | Assess sensitivity to final RoB judgment and comparator type. | Exploratory sensitivity result; no promotion to a confirmatory conclusion. | Exploratory sensitivity |
| Exploratory: grade 3 hematologic toxicity | Comparator/RoB subset sensitivity | I2=0.0% | Assess sensitivity to final RoB judgment and comparator type. | Exploratory sensitivity result; no promotion to a confirmatory conclusion. | Exploratory sensitivity |
| Exploratory: grade 3 hematologic toxicity | Comparator/RoB subset sensitivity | I2=0.0% | Assess sensitivity to final RoB judgment and comparator type. | Exploratory sensitivity result; no promotion to a confirmatory conclusion. | Exploratory sensitivity |
| Exploratory: expanded hematologic toxicity | Comparator/RoB subset sensitivity | I2=0.0% | Assess sensitivity to final RoB judgment and comparator type. | Exploratory sensitivity result; no promotion to a confirmatory conclusion. | Exploratory sensitivity |
| Exploratory: expanded hematologic toxicity | Comparator/RoB subset sensitivity | I2=0.0% | Assess sensitivity to final RoB judgment and comparator type. | Exploratory sensitivity result; no promotion to a confirmatory conclusion. | Exploratory sensitivity |
| Exploratory: diarrhea event | Comparator/RoB subset sensitivity | Not estimable | Assess sensitivity to final RoB judgment and comparator type. | Exploratory sensitivity result; no promotion to a confirmatory conclusion. | Exploratory sensitivity |
| Exploratory: diarrhea event | Comparator/RoB subset sensitivity | Not estimable | Assess sensitivity to final RoB judgment and comparator type. | Exploratory sensitivity result; no promotion to a confirmatory conclusion. | Exploratory sensitivity |
| Exploratory: gastrointestinal toxicity or diarrhea | Comparator/RoB subset sensitivity | I2=19.5% | Assess sensitivity to final RoB judgment and comparator type. | Exploratory sensitivity result; no promotion to a confirmatory conclusion. | Exploratory sensitivity |
| Exploratory: gastrointestinal toxicity or diarrhea | Comparator/RoB subset sensitivity | Not estimable | Assess sensitivity to final RoB judgment and comparator type. | Exploratory sensitivity result; no promotion to a confirmatory conclusion. | Exploratory sensitivity |
| Exploratory: renal toxicity event | Comparator/RoB subset sensitivity | I2=27.1% | Assess sensitivity to final RoB judgment and comparator type. | Exploratory sensitivity result; no promotion to a confirmatory conclusion. | Exploratory sensitivity |
| Exploratory: renal toxicity event | Comparator/RoB subset sensitivity | I2=27.1% | Assess sensitivity to final RoB judgment and comparator type. | Exploratory sensitivity result; no promotion to a confirmatory conclusion. | Exploratory sensitivity |
| Exploratory: pain grade >=2 | Comparator/RoB subset sensitivity | I2=31.6% | Assess sensitivity to final RoB judgment and comparator type. | Exploratory sensitivity result; no promotion to a confirmatory conclusion. | Exploratory sensitivity |
| Exploratory: pain grade >=2 | Comparator/RoB subset sensitivity | Not estimable | Assess sensitivity to final RoB judgment and comparator type. | Exploratory sensitivity result; no promotion to a confirmatory conclusion. | Exploratory sensitivity |
| Exploratory: severe broad toxicity | Comparator/RoB subset sensitivity | I2=56.0% | Assess sensitivity to final RoB judgment and comparator type. | Exploratory sensitivity result; no promotion to a confirmatory conclusion. | Exploratory sensitivity |
| Exploratory: severe broad toxicity | Comparator/RoB subset sensitivity | I2=62.6% | Assess sensitivity to final RoB judgment and comparator type. | Exploratory sensitivity result; no promotion to a confirmatory conclusion. | Exploratory sensitivity |
| Exploratory: broad toxicity-prevention pool | Comparator/RoB subset sensitivity | I2=57.2% | Assess sensitivity to final RoB judgment and comparator type. | Exploratory sensitivity result; no promotion to a confirmatory conclusion. | Exploratory sensitivity |
| Exploratory: broad toxicity-prevention pool | Comparator/RoB subset sensitivity | I2=63.7% | Assess sensitivity to final RoB judgment and comparator type. | Exploratory sensitivity result; no promotion to a confirmatory conclusion. | Exploratory sensitivity |
| Primary: severe oral mucositis, head-and-neck RT/CRT | REML/Hartung-Knapp robustness analysis | I2=47.2%; tau2=0.163 | Assess small-study and heterogeneity-estimator sensitivity without replacing the accepted baseline model. | Null-exclusion classification unchanged; uncertainty remains governed by the wider robust interval. | Primary robustness |
| Exploratory: severe oral mucositis, expanded contexts | REML/Hartung-Knapp robustness analysis | I2=61.1%; tau2=0.288 | Assess small-study and heterogeneity-estimator sensitivity without replacing the accepted baseline model. | Null-exclusion classification unchanged; uncertainty remains governed by the wider robust interval. | Exploratory robustness |
| Exploratory: any oral mucositis | REML/Hartung-Knapp robustness analysis | I2=0.0%; tau2=0.000 | Assess small-study and heterogeneity-estimator sensitivity without replacing the accepted baseline model. | Null-exclusion classification unchanged; uncertainty remains governed by the wider robust interval. | Exploratory robustness |
| Exploratory: grade 3 hematologic toxicity | REML/Hartung-Knapp robustness analysis | I2=0.0%; tau2=0.000 | Assess small-study and heterogeneity-estimator sensitivity without replacing the accepted baseline model. | Conventional null-exclusion was not retained; model-sensitive. | Exploratory robustness |
| Exploratory: expanded hematologic toxicity | REML/Hartung-Knapp robustness analysis | I2=0.0%; tau2=0.000 | Assess small-study and heterogeneity-estimator sensitivity without replacing the accepted baseline model. | Null-exclusion classification unchanged; uncertainty remains governed by the wider robust interval. | Exploratory robustness |
| Exploratory: diarrhea event | REML/Hartung-Knapp robustness analysis | I2=47.3%; tau2=0.218 | Assess small-study and heterogeneity-estimator sensitivity without replacing the accepted baseline model. | Conventional null-exclusion was not retained; model-sensitive. | Exploratory robustness |
| Exploratory: gastrointestinal toxicity or diarrhea | REML/Hartung-Knapp robustness analysis | I2=1.41e-04%; tau2=1.70e-07 | Assess small-study and heterogeneity-estimator sensitivity without replacing the accepted baseline model. | Conventional null-exclusion was not retained; model-sensitive. | Exploratory robustness |
| Exploratory: renal toxicity event | REML/Hartung-Knapp robustness analysis | I2=27.1%; tau2=0.470 | Assess small-study and heterogeneity-estimator sensitivity without replacing the accepted baseline model. | Null-exclusion classification unchanged; uncertainty remains governed by the wider robust interval. | Exploratory robustness |
| Exploratory: pain grade >=2 | REML/Hartung-Knapp robustness analysis | I2=31.6%; tau2=0.023 | Assess small-study and heterogeneity-estimator sensitivity without replacing the accepted baseline model. | Conventional null-exclusion was not retained; model-sensitive. | Exploratory robustness |
| Exploratory: severe broad toxicity | REML/Hartung-Knapp robustness analysis | I2=58.3%; tau2=0.166 | Assess small-study and heterogeneity-estimator sensitivity without replacing the accepted baseline model. | Null-exclusion classification unchanged; uncertainty remains governed by the wider robust interval. | Exploratory robustness |
| Exploratory: broad toxicity-prevention pool | REML/Hartung-Knapp robustness analysis | I2=55.1%; tau2=0.138 | Assess small-study and heterogeneity-estimator sensitivity without replacing the accepted baseline model. | Null-exclusion classification unchanged; uncertainty remains governed by the wider robust interval. | Exploratory robustness |
| Exploratory: oral mucositis duration in days | REML/Hartung-Knapp robustness analysis | I2=0.0%; tau2=0.000 | Assess small-study and heterogeneity-estimator sensitivity without replacing the accepted baseline model. | Conventional null-exclusion was not retained; model-sensitive. | Exploratory robustness |
| Exploratory: oral mucositis onset in days | REML/Hartung-Knapp robustness analysis | I2=23.2%; tau2=0.479 | Assess small-study and heterogeneity-estimator sensitivity without replacing the accepted baseline model. | Null-exclusion classification unchanged; uncertainty remains governed by the wider robust interval. | Exploratory robustness |
| Severe oral mucositis with conference-abstract augmentation | Conference-augmented sensitivity analysis | I2=51.9%; tau2=0.104 | Stress-test the accepted full-report analysis without promoting conference-only evidence. | Supplementary sensitivity only; accepted pooled outcomes and the primary inference are unchanged. | Conference-augmented exploratory sensitivity |
| Severe oral mucositis with conference-abstract augmentation | Conference-augmented sensitivity analysis | I2=62.8%; tau2=0.163 | Stress-test the accepted full-report analysis without promoting conference-only evidence. | Supplementary sensitivity only; accepted pooled outcomes and the primary inference are unchanged. | Conference-augmented exploratory sensitivity |
| Severe oral mucositis in transplant settings | Conference-augmented sensitivity analysis | I2=78.4%; tau2=0.000 | Stress-test the accepted full-report analysis without promoting conference-only evidence. | Supplementary sensitivity only; accepted pooled outcomes and the primary inference are unchanged. | Conference-augmented exploratory sensitivity |
| Severe oral mucositis in transplant settings | Conference-augmented sensitivity analysis | I2=78.4%; tau2=0.545 | Stress-test the accepted full-report analysis without promoting conference-only evidence. | Supplementary sensitivity only; accepted pooled outcomes and the primary inference are unchanged. | Conference-augmented exploratory sensitivity |
| Diarrhea with conference-abstract augmentation | Conference-augmented sensitivity analysis | I2=0.0%; tau2=0.000 | Stress-test the accepted full-report analysis without promoting conference-only evidence. | Supplementary sensitivity only; accepted pooled outcomes and the primary inference are unchanged. | Conference-augmented exploratory sensitivity |
| Diarrhea with conference-abstract augmentation | Conference-augmented sensitivity analysis | I2=6.00e-04%; tau2=1.31e-06 | Stress-test the accepted full-report analysis without promoting conference-only evidence. | Supplementary sensitivity only; accepted pooled outcomes and the primary inference are unchanged. | Conference-augmented exploratory sensitivity |
| Broad toxicity-prevention pool with conference-abstract augmentation | Conference-augmented sensitivity analysis | I2=73.2%; tau2=0.156 | Stress-test the accepted full-report analysis without promoting conference-only evidence. | Supplementary sensitivity only; accepted pooled outcomes and the primary inference are unchanged. | Conference-augmented exploratory sensitivity |
| Broad toxicity-prevention pool with conference-abstract augmentation | Conference-augmented sensitivity analysis | I2=74.8%; tau2=0.169 | Stress-test the accepted full-report analysis without promoting conference-only evidence. | Supplementary sensitivity only; accepted pooled outcomes and the primary inference are unchanged. | Conference-augmented exploratory sensitivity |
| Pelvic chemoradiotherapy grade 2-3 diarrhea | Derived OR sensitivity analysis | I2=0.0%; tau2=0.000 | Assess whether a same-direction pelvic-diarrhea signal is visible while preserving OR/RR separation. | Supplementary sensitivity only; accepted pooled outcomes and the primary inference are unchanged. | Derived exploratory OR sensitivity |
| Pelvic chemoradiotherapy grade 2-3 diarrhea | Derived OR sensitivity analysis | I2=0.0%; tau2=0.000 | Assess whether a same-direction pelvic-diarrhea signal is visible while preserving OR/RR separation. | Supplementary sensitivity only; accepted pooled outcomes and the primary inference are unchanged. | Derived exploratory OR sensitivity |

*Note. Five conventional exploratory signals were model-sensitive under Hartung-Knapp: grade 3 hematologic toxicity, diarrhea, gastrointestinal toxicity, pain grade >=2, and oral-mucositis duration. No result was promoted because of statistical significance.*

**Supplementary Table S10. Publication-bias and small-study-effect assessment**

**Panel A. Assessment decision and assumptions**

| **Outcome** | **Studies** | **Assessment method** | **Test statistic** | **P value** | **Assumptions met** |
| --- | --- | --- | --- | --- | --- |
| Primary: severe oral mucositis, head-and-neck RT/CRT | 4 | Not performed because fewer than 10 studies contributed. | Not applicable | Not applicable | No |
| Exploratory: severe oral mucositis, expanded contexts | 5 | Not performed because fewer than 10 studies contributed. | Not applicable | Not applicable | No |
| Exploratory: any oral mucositis | 2 | Not performed because fewer than 10 studies contributed. | Not applicable | Not applicable | No |
| Exploratory: grade 3 hematologic toxicity | 2 | Not performed because fewer than 10 studies contributed. | Not applicable | Not applicable | No |
| Exploratory: expanded hematologic toxicity | 4 | Not performed because fewer than 10 studies contributed. | Not applicable | Not applicable | No |
| Exploratory: diarrhea event | 2 | Not performed because fewer than 10 studies contributed. | Not applicable | Not applicable | No |
| Exploratory: gastrointestinal toxicity or diarrhea | 3 | Not performed because fewer than 10 studies contributed. | Not applicable | Not applicable | No |
| Exploratory: renal toxicity event | 2 | Not performed because fewer than 10 studies contributed. | Not applicable | Not applicable | No |
| Exploratory: pain grade >=2 | 2 | Not performed because fewer than 10 studies contributed. | Not applicable | Not applicable | No |
| Exploratory: severe broad toxicity | 7 | Not performed because fewer than 10 studies contributed. | Not applicable | Not applicable | No |
| Exploratory: broad toxicity-prevention pool | 11 | Not performed because the broad pool combines clinically distinct toxicity domains and does not support a stable common funnel-plot interpretation. | Not applicable | Not applicable | No |
| Exploratory: oral mucositis duration in days | 2 | Not performed because fewer than 10 studies contributed. | Not applicable | Not applicable | No |
| Exploratory: oral mucositis onset in days | 2 | Not performed because fewer than 10 studies contributed. | Not applicable | Not applicable | No |

**Panel B. Interpretation and limitations**

| **Outcome** | **Funnel plot link** | **Interpretation** | **Limitations** |
| --- | --- | --- | --- |
| Primary: severe oral mucositis, head-and-neck RT/CRT | Not applicable | Publication bias and small-study effects could not be reliably assessed. | Low power and unstable asymmetry tests with small k. |
| Exploratory: severe oral mucositis, expanded contexts | Not applicable | Publication bias and small-study effects could not be reliably assessed. | Low power and unstable asymmetry tests with small k. |
| Exploratory: any oral mucositis | Not applicable | Publication bias and small-study effects could not be reliably assessed. | Low power and unstable asymmetry tests with small k. |
| Exploratory: grade 3 hematologic toxicity | Not applicable | Publication bias and small-study effects could not be reliably assessed. | Low power and unstable asymmetry tests with small k. |
| Exploratory: expanded hematologic toxicity | Not applicable | Publication bias and small-study effects could not be reliably assessed. | Low power and unstable asymmetry tests with small k. |
| Exploratory: diarrhea event | Not applicable | Publication bias and small-study effects could not be reliably assessed. | Low power and unstable asymmetry tests with small k. |
| Exploratory: gastrointestinal toxicity or diarrhea | Not applicable | Publication bias and small-study effects could not be reliably assessed. | Low power and unstable asymmetry tests with small k. |
| Exploratory: renal toxicity event | Not applicable | Publication bias and small-study effects could not be reliably assessed. | Low power and unstable asymmetry tests with small k. |
| Exploratory: pain grade >=2 | Not applicable | Publication bias and small-study effects could not be reliably assessed. | Low power and unstable asymmetry tests with small k. |
| Exploratory: severe broad toxicity | Not applicable | Publication bias and small-study effects could not be reliably assessed. | Low power and unstable asymmetry tests with small k. |
| Exploratory: broad toxicity-prevention pool | Not applicable | Publication bias and small-study effects could not be reliably assessed. | Clinical heterogeneity would make asymmetry difficult to distinguish from outcome-domain differences. |
| Exploratory: oral mucositis duration in days | Not applicable | Publication bias and small-study effects could not be reliably assessed. | Low power and unstable asymmetry tests with small k. |
| Exploratory: oral mucositis onset in days | Not applicable | Publication bias and small-study effects could not be reliably assessed. | Low power and unstable asymmetry tests with small k. |

*Note. Formal tests were not used when k was small. The only pool with at least 10 studies was deliberately broad and clinically heterogeneous, so an asymmetry test would not have a stable interpretation.*

**Supplementary Table S11. Summary of findings and GRADE certainty**

**Panel A. Effect estimates, participants, and certainty**

| **Outcome** | **Anticipated absolute effect or baseline risk** | **Relative effect** | **Participants** | **Studies** | **Certainty** |
| --- | --- | --- | --- | --- | --- |
| severe oral mucositis, head-and-neck RT/CRT | Observed control risk 438/1000; estimated selenium risk 389/1000 (220 to 686); difference -49/1000 (-218 to +248). | RR 0.89 (0.50 to 1.56) | 188 | 4 | Very low |
| severe oral mucositis, expanded contexts | Observed control risk 413/1000; estimated selenium risk 294/1000 (157 to 549); difference -119/1000 (-256 to +136). | RR 0.71 (0.38 to 1.33) | 262 | 5 | Very low |
| any oral mucositis | Observed control risk 901/1000; estimated selenium risk 884/1000 (823 to 950); difference -17/1000 (-78 to +49). | RR 0.98 (0.91 to 1.05) | 145 | 2 | Low |
| grade 3 hematologic toxicity | Observed control risk 571/1000; estimated selenium risk 398/1000 (285 to 555); difference -173/1000 (-286 to -16). | RR 0.70 (0.50 to 0.97) | 138 | 2 | Low |
| expanded hematologic toxicity | Observed control risk 380/1000; estimated selenium risk 265/1000 (192 to 366); difference -115/1000 (-188 to -14). | RR 0.70 (0.50 to 0.96) | 242 | 4 | Very low |
| diarrhea event | Observed control risk 302/1000; estimated selenium risk 181/1000 (111 to 295); difference -121/1000 (-191 to -7). | RR 0.60 (0.37 to 0.98) | 185 | 2 | Very low |
| gastrointestinal toxicity or diarrhea | Observed control risk 360/1000; estimated selenium risk 218/1000 (152 to 315); difference -142/1000 (-208 to -45). | RR 0.61 (0.42 to 0.88) | 271 | 3 | Very low |
| renal toxicity event | Observed control risk 143/1000; estimated selenium risk 45/1000 (14 to 143); difference -98/1000 (-129 to +0). | RR 0.31 (0.10 to 1.00) | 196 | 2 | Very low |
| pain grade >=2 | Observed control risk 767/1000; estimated selenium risk 598/1000 (488 to 732); difference -169/1000 (-279 to -35). | RR 0.78 (0.64 to 0.95) | 146 | 2 | Low |
| severe broad toxicity | Not summarized because this deliberately broad pool combines distinct toxicity domains and baseline risks. | RR 0.71 (0.46 to 1.09) | 452 | 7 | Very low |
| broad toxicity-prevention pool | Not summarized because this deliberately broad pool combines distinct toxicity domains and baseline risks. | RR 0.60 (0.42 to 0.83) | 745 | 11 | Very low |
| oral mucositis duration in days | Not calculated for a continuous outcome | Not applicable for MD | 145 | 2 | Very low |
| oral mucositis onset in days | Not calculated for a continuous outcome | Not applicable for MD | 131 | 2 | Very low |

**Panel B. Certainty rationale and interpretation**

| **Outcome** | **Certainty** | **Reasons for certainty rating** | **Interpretation** |
| --- | --- | --- | --- |
| severe oral mucositis, head-and-neck RT/CRT | Very low | Risk-of-bias concerns, moderate heterogeneity, non-placebo sensitivity differences, and confidence interval compatible with benefit or harm. | The effect remains uncertain and does not establish a preventive benefit. |
| severe oral mucositis, expanded contexts | Very low | Expanded contexts increase indirectness; heterogeneity is substantial and the interval remains inconclusive. | Exploratory evidence only; the estimate should not be interpreted as confirmatory. |
| any oral mucositis | Low | Two-study exploratory estimate with limited outcome scope and RoB. | Exploratory evidence only; the estimate should not be interpreted as confirmatory. |
| grade 3 hematologic toxicity | Low | Signal favors selenium, but only two studies and RoB prevent a firm conclusion. Hartung-Knapp sensitivity crossed the null, confirming model sensitivity. | The conventional estimate is model-sensitive and should not be treated as a robust benefit. |
| expanded hematologic toxicity | Very low | Expanded definitions and Chen 2023 extraction uncertainty require cautious interpretation despite low statistical heterogeneity. | Exploratory evidence only; the estimate should not be interpreted as confirmatory. |
| diarrhea event | Very low | Two-study exploratory estimate with moderate heterogeneity and RoB. Hartung-Knapp sensitivity crossed the null, confirming model sensitivity. | The conventional estimate is model-sensitive and should not be treated as a robust benefit. |
| gastrointestinal toxicity or diarrhea | Very low | Combined GI toxicity and diarrhea definitions are indirect, and Chen 2023 contains an inconsistency in reported percentages. Hartung-Knapp sensitivity crossed the null, confirming model sensitivity. | The conventional estimate is model-sensitive and should not be treated as a robust benefit. |
| renal toxicity event | Very low | Very small two-study evidence base with boundary-touching CI and RoB. | Exploratory evidence only; the estimate should not be interpreted as confirmatory. |
| pain grade >=2 | Low | The two-study pain outcome has a consistent signal but remains exploratory, context-sensitive, and affected by reporting uncertainty. Hartung-Knapp sensitivity crossed the null, confirming model sensitivity. | The conventional estimate is model-sensitive and should not be treated as a robust benefit. |
| severe broad toxicity | Very low | Broad severe-toxicity grouping crosses domains and remains statistically inconclusive. | Exploratory evidence only; the estimate should not be interpreted as confirmatory. |
| broad toxicity-prevention pool | Very low | This is a deliberately broad exploratory pool across toxicity domains and should not drive the primary conclusion. | Exploratory evidence only; the estimate should not be interpreted as confirmatory. |
| oral mucositis duration in days | Very low | Continuous duration outcome is compatible with shorter duration but is scope-sensitive and based on two studies. Hartung-Knapp sensitivity crossed the null, confirming model sensitivity. | The conventional estimate is model-sensitive and should not be treated as a robust benefit. |
| oral mucositis onset in days | Very low | Two small trials used different radiotherapy contexts; the confidence interval crosses no effect and the REML/Hartung-Knapp interval is very wide. Laali weeks were transparently converted to days. | Exploratory evidence only; the estimate should not be interpreted as confirmatory. |

*Note. Certainty was rated for 13 pooled outcomes: 3 Low and 10 Very low. The primary severe oral-mucositis outcome was Very low certainty. Conference-augmented and derived-OR sensitivities were not counted as accepted pooled outcomes. Absolute effects use the observed aggregate control risk only where a clinically coherent RR outcome allowed a descriptive calculation.*

Supplementary Table S12. Supplementary safety and contextual evidence

**Panel A. Study design and comparison**

| **Study** | **Evidence role** | **Design** | **Setting** | **Selenium intervention** | **Comparator or design** |
| --- | --- | --- | --- | --- | --- |
| Zakharia 2025 | Supplementary safety/context | Phase I open-label single-arm dose escalation/expansion | Metastatic clear-cell renal cell carcinoma treated with axitinib | High-dose oral seleno-L-methionine with axitinib | No comparator; single-arm phase I |
| Evans 2020 | Supplementary safety/context | Clinical pharmacodynamic selenium compound comparison | Cancer-related selenium compound pharmacodynamic context | Selenium compound comparison | Pharmacodynamic comparison |
| Knox 2019 | Supplementary safety/context | Phase I dose-escalation trial | Cancer phase I selenium safety context | Selenium-containing investigational regimen | No comparative prevention control |
| Han 2019 | Supplementary safety/context | Randomized placebo-controlled trial | Prostate/localized cancer or surveillance context | Selenium supplementation | Placebo |
| Song 2018 | Supplementary safety/context | Phase I 3+3 dose escalation | Gynecologic cancers receiving selenium plus chemotherapy | Selenium plus chemotherapy | Single-arm dose escalation |
| Brodin 2015 | Supplementary safety/context | Phase I trial | Cancer radiotherapy/systemic treatment safety context | Selenium intervention | Phase I design |
| Fakih 2008 | Supplementary safety/context | Phase I pharmacokinetic study | Solid tumor selenium pharmacokinetic context | Selenium-containing regimen | Phase I/PK |
| Fakih 2006 | Supplementary safety/context | Phase I pharmacokinetic study | Solid tumor selenium pharmacokinetic context | Selenium-containing regimen | Phase I/PK |
| Zimmermann 2005 | Supplementary safety/context | Double-blind randomized prospective study | Lymphedema or scope-sensitive supportive setting | Selenium supplementation | Controlled prospective design |
| Micke 2003 | Supplementary safety/context | Clinical treatment study | Lymphedema/supportive clinical context | Selenium treatment | Clinical treatment context |
| Buentzel 1999 | Supplementary safety/context | German clinical experience/report; controlled status unclear | Head-and-neck RT/radiochemotherapy supportive context | Sodium selenite context | Unclear controlled status |
| Hehr 1997 | Supplementary safety/context | German preliminary clinical report | Radiotherapy supportive context | Sodium selenite context | Preliminary report |
| Corcoran 2010 | Supplementary safety/context | Open-label phase I dose-escalation study | Cancer selenium PK/safety context | Selenium compound | Open-label phase I |
| Rajan 2013 | Supplementary safety/context | Single-arm phase II multicenter conference abstract | Cancer supportive/treatment context | Selenium compound | Conference abstract; single arm |
| Orvain 2025 | Supplementary safety/context | Double-blind randomized conference abstract | Lymphoma/myeloma undergoing high-dose therapy/HSCT context | Intravenous selenium | Conference abstract randomized design |
| Sieja 2004 | Supplementary safety/context | Randomized double-blind placebo-controlled trial | Pomeranian Academy of Medicine, Szczecin, Poland | Protecton Zellactiv, two capsules four times daily for 3 months; multicomponent product containing selenium yeast with Se 50 ug per two capsules | Placebo containing all remaining components without selenium |
| Mix/Rajan 2015 | Supplementary safety/context | Prospective single-arm phase II study | Roswell Park Cancer Institute and Waikato Hospital | Oral L-selenomethionine: 4800 ug twice daily loading for 1 week before CCRT, then 4800 ug daily during CCRT | No concurrent control; compared descriptively to protocol expected historical toxicity rates |
| Alhazmi 2025 | Background Only | Retrospective pilot study | Seoul St. Mary's Hospital, Korea | Sodium selenite pentahydrate 2000 ug/40 mL IV over 2 hours before each XELOX cycle | Non-selenium retrospective comparison group |

**Panel B. Safety context, findings, and source**

| **Study** | **Cancer-therapy context** | **Safety or background focus** | **Minimal safety finding** | **Reason not in main effect table** | **Source** |
| --- | --- | --- | --- | --- | --- |
| Zakharia 2025 | Previously treated metastatic clear-cell renal cell carcinoma | Treatment-emergent adverse events and dose-limiting toxicity | Safety/PK context only; detailed AE table not included in the main effect table | Phase I single-arm anticancer combination; not toxicity prevention comparative trial | Zakharia 2025 full-text report |
| Evans 2020 | Not primary cancer therapy toxicity prevention | Pharmacodynamic/background safety | Supplementary context only | Not a primary comparative prevention trial for therapy-related toxicity | Evans 2020 full-text report |
| Knox 2019 | Phase I oncology treatment context | Dose escalation, tolerability, DLT | Supplementary safety context only | Phase I safety-only design | Knox 2019 full-text report |
| Han 2019 | Not cancer therapy-related toxicity prevention outside the prespecified scope | Supplementary safety/background | Not included in the main effect table | Scope-sensitive; not primary therapy-related toxicity prevention | Han 2019 full-text report |
| Song 2018 | Gynecologic cancer chemotherapy | Dose escalation, DLT, treatment-emergent AEs | Safety-only context; no comparative prevention effect extracted | Phase I single-arm design | Song 2018 full-text report |
| Brodin 2015 | Cancer treatment context | Tolerability and dose finding | Supplementary safety only | Phase I design | Brodin 2015 full-text report |
| Fakih 2008 | Solid tumor treatment context | PK, tolerability, dose escalation | Supplementary safety/PK only | Phase I/PK, not prevention comparative trial | Fakih 2008 full-text report |
| Fakih 2006 | Solid tumor treatment context | PK, tolerability, dose escalation | Supplementary safety/PK only | Phase I/PK, not prevention comparative trial | Fakih 2006 full-text report |
| Zimmermann 2005 | Established lymphedema treatment/alleviation context | Lymphedema treatment context | Supplementary context only | Established lymphedema treatment/alleviation, not primary prevention/reduction of cancer therapy toxicity | Zimmermann 2005 full-text report |
| Micke 2003 | Established lymphedema treatment/alleviation context | Treatment/alleviation and safety context | Supplementary only | Lymphedema treatment scope-sensitive, not primary prevention synthesis | Micke 2003 full-text report |
| Buentzel 1999 | Head-and-neck cancer treatment toxicity context | Historical toxicity prevention/supportive care signal | German report not included in the main effect table | Language/controlled-status uncertainty | Buentzel 1999 full-text report |
| Hehr 1997 | Radiotherapy toxicity context | Historical background | German preliminary report not included in the main effect table | Language/preliminary report uncertainty | Hehr 1997 full-text report |
| Corcoran 2010 | Oncology treatment context | Dose escalation, PK, tolerability | Supplementary safety/PK only | Phase I open-label design | Corcoran 2010 full-text report |
| Rajan 2013 | Oncology treatment context | Conference-only safety/feasibility signal | Conference abstract only; not included in the main effect table | Conference-only and single-arm | Rajan 2013 full-text report |
| Orvain 2025 | Lymphoma or myeloma high-dose therapy context | Oral mucositis prevention signal and safety context | Potentially relevant but conference-only; not included in the main comparative effect table | Conference abstract; not eligible for quantitative synthesis | Orvain 2025 full-text report |
| Sieja 2004 | Ovarian cancer surgery followed by cisplatin plus cyclophosphamide chemotherapy | Oxidative stress, selenium status, hematology, and chemotherapy side effects | Reported significant decreases over time in hair loss, flatulence, abdominal pain, weakness, malaise, and appetite loss; WBC increased after 3 months; no GCSF used. | Intervention is multicomponent antioxidant/vitamin product, so selenium-specific attribution is unclear. | Sieja 2004 full-text report |
| Mix/Rajan 2015 | Inoperable stage III NSCLC concurrent thoracic radiation plus weekly paclitaxel/carboplatin | Acute toxicity and selenium tolerability | No selenium-related toxicity; grade 3-4 esophagitis 3/16, pneumonitis 0/16, anemia 1/16, leukopenia 2/16, neutropenia 0/16. | Single-arm non-comparative safety study; not eligible for selenium-vs-control effect pooling. | Mix/Rajan 2015 full-text report |
| Alhazmi 2025 | Stage III gastric adenocarcinoma after gastrectomy receiving adjuvant XELOX | Chemotherapy-induced peripheral neuropathy and high-dose IV selenium safety | No adverse events related to high-dose selenium; no significant differences in nausea/vomiting, diarrhea, hand-foot syndrome, or appetite loss; no severe CIPN in either group. | Retrospective observational design; retained as background/context only. | Alhazmi 2025 full-text report |

*Note. These reports inform safety, feasibility, or context but were not entered into the main comparative effect table when attribution, design, or outcome structure was incompatible.*

# 2 Supplementary Figures

**Supplementary Figure S1.** Expanded severe oral mucositis. Exploratory forest plot across head-and-neck radiotherapy/chemoradiotherapy and transplantation settings.

**Supplementary Figure S2.** Any oral mucositis. Exploratory forest plot for any-grade oral mucositis.

**Supplementary Figure S3.** Grade 3 hematologic toxicity. Exploratory forest plot; the conventional null-excluding estimate was not retained under Hartung-Knapp analysis.

**Supplementary Figure S4.** Expanded hematologic toxicity. Exploratory forest plot using the wider hematologic outcome definition.

**Supplementary Figure S5.** Diarrhea. Exploratory forest plot; the conventional null-excluding estimate was not retained under Hartung-Knapp analysis.

**Supplementary Figure S6.** Gastrointestinal toxicity or diarrhea. Exploratory forest plot using the wider gastrointestinal definition; the conventional null-excluding estimate was not retained under Hartung-Knapp analysis.

**Supplementary Figure S7.** Renal toxicity. Exploratory forest plot for renal toxicity events.

**Supplementary Figure S8.** Pain grade 2 or higher. Exploratory forest plot; the conventional null-excluding estimate was not retained under Hartung-Knapp analysis.

**Supplementary Figure S9.** Severe broad toxicity. Exploratory forest plot across severe toxicity domains.

**Supplementary Figure S10.** Broad toxicity-prevention pool. Cross-domain exploratory forest plot. This analysis evaluates directional consistency and is not a domain-specific efficacy estimate.

**Supplementary Figure S11.** Oral mucositis duration. Scope-sensitive exploratory forest plot in days; the conventional null-excluding estimate was not retained under Hartung-Knapp analysis.

Supplementary Figure S12. Risk-of-bias traffic-light matrix. Final domain-level and overall judgments for the 17 main-analysis studies.

**Supplementary Figure S13.** Risk-of-bias summary. Percentage distribution of Low risk, Some concerns, and High risk judgments by domain and overall.

**Supplementary Figure S14.** Oral mucositis onset. Two-study exploratory mean-difference forest plot in days. Positive values indicate later onset with selenium. Laali 2020 means and standard deviations were multiplied by seven to convert weeks to days. The fixed-effect estimate is accompanied by REML/Hartung-Knapp robustness results.

**Supplementary Figure S15.** Severe oral mucositis with conference-abstract augmentation. Supplementary stress test adding Orvain 2025 grade 3-4 oral mucositis events to the expanded full-report pool. Orvain 2025 was available only as a conference abstract, so this analysis does not replace an accepted pooled outcome.

**Supplementary Figure S16.** Diarrhea with conference-abstract augmentation. Threshold-mixed stress test adding Orvain 2025 grade 3-4 diarrhea events to studies reporting any diarrhea or grade 1-3 diarrhea. The conventional interval excluded the null, whereas the Hartung-Knapp interval did not.

**Supplementary Figure S17.** Broad toxicity with conference-abstract augmentation. Cross-domain stress test adding Orvain 2025 grade 3-4 all-adverse-event data to the broad toxicity-prevention pool. The analysis is exploratory because toxicity constructs and treatment settings differ substantially.

**Supplementary Figure S18.** Derived pelvic-diarrhea odds-ratio sensitivity. Fixed-effect synthesis of the Bahador 2022 reported grade 2-3 versus grade 0 diarrhea OR and the Muecke 2010 OR reconstructed from week-4 category counts. Direction is no selenium versus selenium. The Bahador standard error was approximated from a rounded P value, grade 1 was excluded from the Muecke contrast, and the result was not combined with RR pools.
